# Charge Ordering and Incommensurate Modulations in the Metamagnetic Layered Manganese Oxysulfide Sr_2_MnO_2_Cu_3.5_S_3_

**DOI:** 10.1021/jacs.5c21494

**Published:** 2026-01-16

**Authors:** Lemuel E. Crentsil, Oliver J. Rutt, David G. Free, Murray J. David, Robert D. Smyth, Catherine F. Smura, David A. Keen, Andrew N. Fitch, Joke Hadermann, Simon J. Clarke

**Affiliations:** † Department of Chemistry, 6396 University of Oxford, Inorganic Chemistry Laboratory, South Parks Road, Oxford OX1 3QR, U.K.; ‡ ISIS Facility, Rutherford Appleton Laboratory, Harwell Campus, Didcot, Oxfordshire OX11 0QX, U.K.; § European Synchrotron Radiation Facility-71, Avenue des Martyrs, CS 40220, 38043 Grenoble, Cedex 9, France; ∥ Electron Microscopy for Materials Science (EMAT), University of Antwerp, Groenenborgerlaan 171, B-2020 Antwerp, Belgium

## Abstract

Sr_2_MnO_2_Cu_3.5_S_3_ contains mixed-valent Mn ions Mn^2+/3+^ in axially elongated MnO_4_S_2_ octahedra connected via apical sulfide anions to copper-deficient antifluorite-type Cu_4‑δ_S_3_ layers where δ ∼ 0.5. Copper deficiency is charge-compensated by oxidation of Mn 3d states resulting in mixed-valency. The compound is tetragonal in *P*4/*mmm* at ambient temperatures (*a* = 4.016345(1) Å, *c* = 11.40708(5) Å). Below 190 K, superlattice reflections in diffraction data and an increase in resistivity, signal checkerboard charge-ordering of Mn^2+^ and Mn^3+^. The superstructure approximates to a √2*a* × √2*a* × 2*c* expansion of the room temperature cell in space group *P*4_2_/*nmc*. However, satellite reflections signal a (3 + 2)­D incommensurate modulation of Cu site occupancies in the Cu-deficient sulfide layers coupled with displacements of the sulfur positions; overall the superstructure below 190 K requires description in superspace group *P*4_2_/*nmc*(*a*,0,0)­0000­(0,*a*,0)­00s0. Analysis of total scattering measurements along with pair distribution functions supports the charge-ordered low temperature model and reveals local order of distinct Mn sites within the higher-temperature charge-disordered regime. Below *T*_N_ = 27 K, long-range magnetic ordering is A-type antiferromagnetic with distinct moments for Mn^2+^ and Mn^3+^ ions directed perpendicular to the MnO_2_ planes and ordered ferromagnetically. Long-range antiferromagnetic order results from interlayer antiferromagnetic coupling. A metamagnetic transition at 1.1 T corresponds to a change to long-range interlayer ferromagnetic ordering via a spin-reorientation of magnetic moments and is associated with a slight decrease in the charge separation between the Mn sublattices, consistent with observations on mixed-valent perovskite and Ruddlesden–Popper-type oxide manganites.

## Graphical abstract

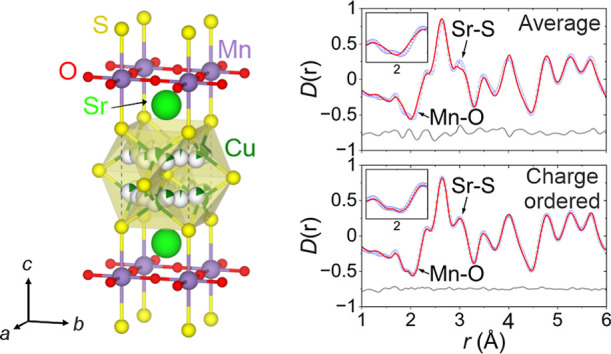

## Introduction

Transition metal compounds containing both oxide and anions of less electronegative elements such as a chalcogen (*Ch* = S, Se, Te) are a class of materials from which new physical and chemical properties, which are inaccessible in the traditional single-anion analogues, may emerge. Ordering of anions on account of their differing polarizabilities and chemistries can be harnessed to promote low-dimensional structures with anions coordinating to different metals in distinct layers in accordance with hard–soft acid–base theory. The dimensional reduction afforded by layered structures results in exotic magnetic and electronic properties such as high *T*_C_ superconductivity^1,2^ and efficient thermoelectric performance.^3–5^

A common crystal structure adopted by quinary oxide chalcogenides is the *A*_2_ *M*O_2_Cu_2_ *Ch*_2_ structure (*A* = Sr, Ba; M = mid-to-late 3*d* transition metal), initially reported for quinary compounds by Zhu and Hor.^6^ In this structure, *A*_2_ *M*O_2_ layers containing *M*O_2_ square planar sheets and representing fragments of the perovskite structure are separated by anti-PbO-type Cu_2_ *Ch*_2_ layers containing copper ions (Cu­(I)) in edge-sharing Cu*Ch*_4_ tetrahedra. The stoichiometric compounds contain transition metals of formal oxidation state +2 within highly anisotropic ligand fields due to the elongated octahedral *M*O_4_S_2_ environments imposed by the ordering of oxide and chalcogenide ions and shown for the case of the title compound in Figure 1. The compositional flexibility allows for variable 3d^*n*^ configurations to be hosted within the square planar *M*O_2_ sheets, leading to a wide range of magnetic, electronic and thermal properties.^7–12^

**Figure 1:**
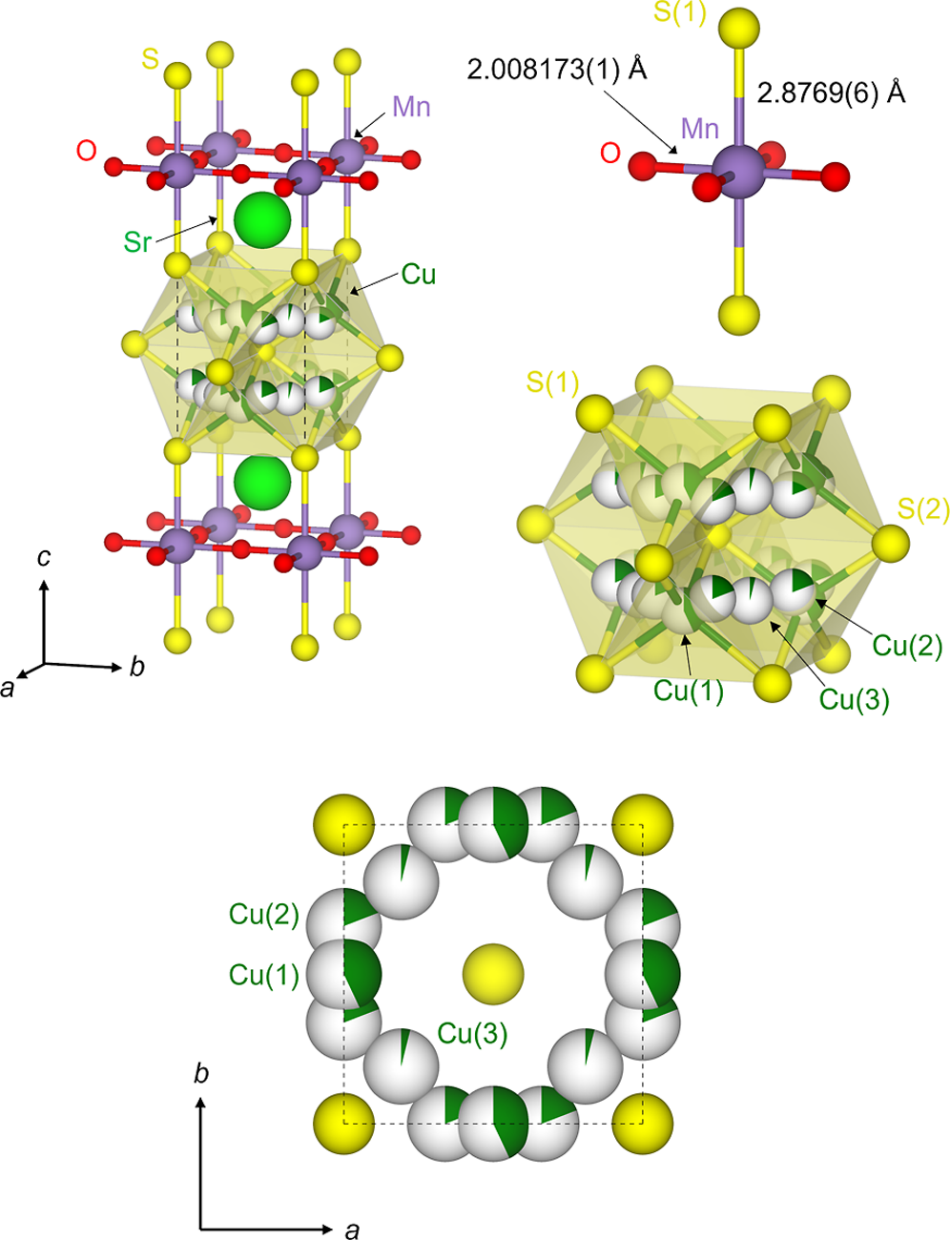
Crystal structure of Sr_2_MnO_2_Cu_3.5_S_3_ (the *n* = 1, *m* = 2 member of the series Sr_*n*+1_Mn_*n*_O_3*n*–1_Cu_2*m*–δ_S_*m*+1_ (*n* = 1–3; *m* = 1–3)) derived from the combined refinement against ambient temperature SXRPD data collected on ID22 and NPD data collected on GEM highlighting the local coordination around manganese and copper. The partial filling of the spheres representing the copper atoms reflect the copper site occupancy factors.

The homologous series of compounds with general formula Sr_*n*+1_Mn_*n*_O_3*n*–1_Cu_2*m*–δ_S_*m*+1_ (*n* = 1–3; *m* = 1–3) may be derived from the *A*_2_ *M*O_2_Cu_2_ *Ch*_2_ structure by either increasing the thickness of the oxide layer or the copper sulfide layer. The *n* = 1, *m* = 2 member, Sr_2_MnO_2_Cu_3.5_S_3_ (Figure 1), which is the subject of this paper, and the *n* = 1, *m* = 3 member Sr_2_MnO_2_Cu_5.5_S_4_ were initially synthesized and characterized by Gál et al.^13^ Intergrowths of these structures are also possible and the compound with formula Sr_4_Mn_2_O_4_Cu_5_S_5_ consists of single sheets of Sr_2_MnO_2_ separated by alternating “single” and “double” thickness layers of copper sulfide.^14^ All the compounds in this series were found to be semiconducting with the copper deficiencies (δ ∼ 0.5) being charge-compensated by the oxidation of Mn d states resulting in a mean Mn-oxidation state of +2.5, complementing the better-studied mixed-valent oxides containing Mn in oxidation states between +3 and +4.^15–18^ A second consequence of the copper deficiency is considerable copper disorder within the CuS_4_ tetrahedra, particularly in the *m* = 2 and 3 members. The disorder may be modeled using partial occupancies of copper sites at the ideal tetrahedral site and at the triangular faces of the tetrahedra as indicated for the title compound in Figure 1. The high mobility of the copper ions means that the copper sulfide layers are susceptible to deintercalation of copper ions via soft chemistry or electrochemical techniques allowing for control of the electron count on the manganese ions.^19–21^ The disorder within the copper sulfide slabs gives way to long-range copper/vacancy order below room temperature in both the parent phase of the *m* = 1 compound Sr_2_MnO_2_Cu_1.5–*x*_S_2_ (*x* = 0)^8^ and its copper deintercalated derivative, where at *x* ≈ 0.20, the structure exhibits an incommensurate modulation of copper occupancies as well as other structural parameters.^22^ The long-range magnetic ordering is also shown to be sensitive to the Mn electron count^22^ and the chalcogen.^8^

Here we show that the very high degree of disorder inherent to the copper sulfide layers of Sr_2_MnO_2_Cu_3.5_S_3_ leads to long-range copper/vacancy order that cannot be described simply in three-dimensional space, but instead features incommensurate displacements of the copper electron density toward the triangular faces of the CuS_4_ tetrahedra. The behavior of the mixed-valent (Mn^2+/3+^) MnO_2_ square planes complements that of the Mn^3+/4+^ perovskite and Ruddlesden–Popper oxide manganites.

## Experimental Section

### Synthesis

Sr_2_MnO_2_Cu_3.5_S_3_ is air stable, however air-sensitive reactants necessitated the use of an argon-filled glovebox (Glovebox Technology Ltd., with O_2_ and H_2_O contents typically below 1 ppm) for all manipulations of solids. The characterization was performed on two 8 g polycrystalline samples of Sr_2_MnO_2_Cu_3.5_S_3_ (referred to as sample 1 and sample 2) with additional measurements performed on a 5 g polycrystalline sample (sample 3). Sample 1 was synthesized from a stoichiometric mixture of SrS, MnO_2_ (Aldrich 99.999%), Cu_2_S (Alfa Aesar 99.95%), and Cu powder (Alfa Aesar 99.995%). Samples 2 and 3 were synthesized from stoichiometric mixtures of SrS, MnO_2_ (Alfa Aesar 99.999%), Mn powder (Aldrich 99.99%), Cu_2_S and CuO (Alfa Aesar 99.99%). SrS was synthesized by the reaction at 800 °C between SrCO_3_ (Alfa Aesar 99.994%) and CS_2_ (Aldrich 99%) vapor carried by an argon gas flow in a tube furnace (Caution: this must be performed in a fumehood as CS_2_ is toxic and highly flammable; the CS_2_ in the exhaust stream was decomposed by bubbling through hydroxide bleach and KMnO_4_). Cu_2_S was prepared by the solid-state reaction between a stoichiometric mixture of Cu powder (Alfa Aesar 99.995%) and S pieces (Thermo Fisher 99.9995%). The mixture was sealed in an evacuated silica ampule and heated inside a furnace to 400 °C at a rate of 0.1 °C min^–1^ for 24 h before being heated to 700 °C at a rate of 5 °C min^–1^, remaining at this temperature for 4 days before being cooled in the furnace. For the synthesis of the oxysulfide, the reactants were ground together inside an argon-filled dry glovebox using an agate pestle and mortar. The ground powder was pressed into a pellet, placed inside an alumina crucible, and sealed inside an evacuated silica ampule. The entire assembly was then heated in a resistance furnace. The product was found to form in high purity by heating the pelletized reagents to 1000 °C at 10 °C min^–1^, maintaining this temperature for 18 h, followed by furnace cooling.^13^ High-quality single crystals suitable for single crystal X-ray diffraction could be obtained by annealing a portion of sample 1 at 1000 °C for a further 2 weeks, followed by cooling at 1 °C min^–1^.

### Powder Diffraction Measurements

X-ray powder diffraction (XRPD) measurements for detailed structural characterization were made for samples 1 and 3 on beamline I11 at the Diamond Light Source synchrotron (Harwell, UK)^23^ and beamlines ID22 or ID31 at the ESRF synchrotron (Grenoble, France).^24^ (Note that the powder diffractometer at ESRF was relocated from the ID31 beamline to the ID22 beamline in 2014). In both cases the X-ray wavelengths were refined using a Si standard. For the measurements on I11 using 0.82 Å X-rays, samples were ground with amorphous glass to limit X-ray absorption and minimize preferred orientation and were sealed inside 0.5 mm diameter borosilicate glass capillaries. The temperature dependence of the lattice parameters of Sr_2_MnO_2_Cu_3.5_S_3_ (sample 1) was measured using the high-count rate Mythen position sensitive detector PSD of the I11 beamline to continuously collect patterns on cooling the sample from 300 to 100 K at a rate of 6 K min^–1^, with a pattern collected every 1.5 K on average. On ID22/ID31 samples were undiluted in 0.5 mm borosilicate capillaries because more penetrating X-rays (0.35 Å on ID22, 0.4 Å on ID31) were used than on I11. On ID22 and ID31 a bespoke helium flow cryostat was used to access temperatures down to 4 K with the sample exposed to helium exchange gas to avoid beam heating effects; a multianalyzer crystal detector was used to record the patterns. Neutron powder diffraction (NPD) measurements were performed on warming sample 2 from 6 K using the GEM instrument at the ISIS Pulsed Neutron and Muon Facility, UK.^25^ NPD patterns at 5 K at various magnetic fields sweeping from 0 to 5 T and back to 0 T were also obtained on GEM for sample 2. Powder diffraction data were analyzed using TOPAS Academic or Jana2020.^26,27^

### Single Crystal X-ray Diffraction

Suitable crystals extracted from the bulk powder sample 1 were selected under Paratone-N oil, mounted on a MiTeGen loop using the oil drop method and cooled at 1 K min^–1^ using an Oxford Cryosystems CryoStream 700 Plus. Single crystal X-ray diffraction (SCXRD) data were collected using a (Rigaku) Oxford Diffraction XtaLAB Synergy DW diffractometer equipped with a HyPix-Arc 150 detector using Mo Kα (λ = 0.71073 Å) radiation. Data collection and reduction were performed using the CrysAlis PRO software.^28^ Equivalent reflections were merged and diffraction patterns processed using the Crysalis PRO suite. The structure was solved using SUPERFLIP^29^ and refined on *F* ^2^ using Jana2020.^27^

### Electron Diffraction Measurements

Measurements were carried out on a Phillips CM20 transmission electron microscope with a LaB_6_ filament. Cooling was performed in situ using a cooling stage with liquid nitrogen, allowing a base temperature of 110 K to be reached.

### Total Scattering Measurements

Neutron total scattering data for both Rietveld and PDF analysis were also collected using the GEM diffractometer at ISIS.^25^ The polycrystalline sample 1 was weighed and loaded into an 8 mm diameter vanadium can to a height of 6.9 cm and this was placed inside a closed-cycle refrigerator (CCR). Total scattering data were collected up to a *Q*_max_ = 30 Å^–1^ for 8 h at each temperature. The data were corrected and placed on an absolute scale using the GudrunN software.^30^ Background scattering, absorption, multiple scattering and Placzek inelasticity corrections were taken into account during this process. The corrected total scattering data were transformed to the pair distribution function, *D*(*r*), as defined by Keen.^31^ X-ray total scattering data were measured using the I15-1 beamline at the Diamond Light Source, UK.^32^ The samples were loaded in 1 mm diameter borosilicate glass capillaries and measured for 600 s at each temperature. The data were collected using monochromatic 0.161669 Å X-rays (76.69 keV) up to *Q*_max_ = 19 Å^–1^. Similar data corrections and transformations, as discussed above, were applied using the GudrunX software.^30^ Fitting of the PDF using small-box models was performed using the TOPAS Academic software.^26^

### Magnetometry

Magnetic susceptibilities were measured using a Quantum Design MPMS-3 SQUID magnetometer. Approximately 30 mg batches of sample 1 were accurately weighed and contained in gelatin capsules. Measurements of the susceptibility were made on warming after cooling in zero applied field (zero-field-cooled; ZFC) and after cooling in the measuring field (field-cooled; FC). Hysteresis measurements were made after cooling in the field and then measuring a full magnetization isotherm in the range ±7 T.

### Electrical Resistivity Measurements

Resistivity measurements were performed in the standard four-probe configuration using a homemade apparatus capable of measuring resistances in the range 3 × 10^–3^ to 3 × 10^7^ Ω and over the temperature ranges accessible with liquid nitrogen. Samples were prepared by sintering pellets of sample 2 at the synthesis temperature (1000 °C). Rectangular bars of dimensions 5 × 2 × 1 mm^3^ were cut from the sintered pellets using a diamond saw. Four copper contact wires (0.1 mm diameter; Alfa Aesar 99.9985%) were attached along the rectangular bar using electrically conducting silver-laden epoxy adhesive. Measurements were performed in the temperature range 70–300 K using a DC-force-current-measure-voltage method by forcing a constant current to flow through the bar via the outer two contacts while the potential difference across the inner two contacts was measured.

## Results and Discussion

### Room Temperature Crystal Structure

A combined Rietveld refinement against synchrotron X-ray (ID22) and neutron powder diffraction (GEM) data measured at ambient temperature (sample 1) was carried out using the published structure of Sr_2_MnO_2_Cu_3.5_S_3_ in space group *P*4/*mmm* as the starting model.^13^ Positional and anisotropic displacement parameters were refined, as well as copper site occupancies. In addition to the main oxysulfide phase, Bragg peaks arising from a small SrS impurity (1% by mass) and elemental vanadium from the sample can in the neutron data (most intense reflection at ∼2.1 Å), were present in the pattern. Repeated grinding and reheating were not successful in reducing the amount of the SrS impurity. The refined structure is shown in Figure 1 and the Rietveld fits to the synchrotron XRPD data and NPD data are shown in Figure 2 (see also Figure S3). Refined structural parameters are given in Table 1. The refined copper site occupancies were consistent with the deficiency (relative to full occupancy of tetrahedral sites in the sulfide layers) of ∼0.5 Cu ions in the sulfide layer per Mn ion in the formula unit (i.e Sr_2_MnO_2_Cu_4–0.5_S_3_). SEM imaging of several crystallites confirmed an even distribution of the elements and semiquantitative EDX analysis was consistent with the refinement, giving an elemental ratio of Sr/Mn/Cu/S = 2:1.2:3.4:3.1 (Figure S2). As previously reported,^13^ the copper ions were found to be highly delocalized across several sites within the antifluorite-type copper sulfide layers as shown in Figure 1. In addition to copper density located at the ideal tetrahedral Cu(1) site (4i), copper density was also observed at the less highly coordinated Cu(2) triangular sites (8s) and at a site displaced toward the vacant octahedral site (Cu(3)) within the copper sulfide layer.

**Figure 2:**
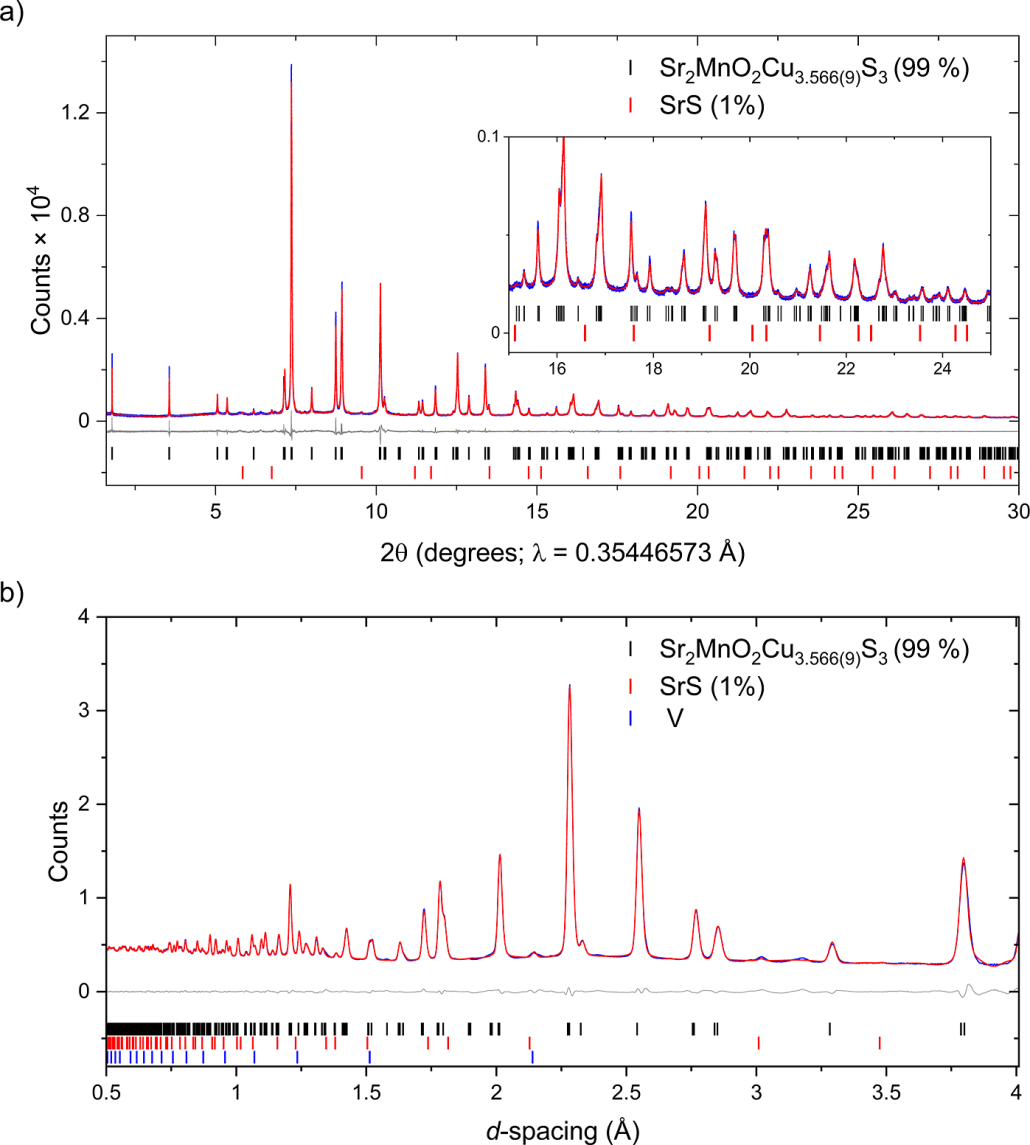
Rietveld refinement of the nuclear structure of Sr_2_MnO_2_Cu_3.5_S_3_ (sample 1) against (a) synchrotron XRPD data (ID22) and (b) NPD data from bank 4 (63.62°) of the GEM instrument collected at room temperature (see also Figure S3 for refinements against the remaining banks). The data (blue), calculated pattern (red), and difference (gray) are shown. *R*_wp_ = 3.926%. The weak Bragg scattering from the vanadium container has been included as an additional phase (not included in the weight percentages given).

**Table 1:** Refined Structural Parameters for Sr_2_MnO_2_Cu_3.566(9)_S_3_ (Sample 1) at Room Temperature from Combined Refinement against I11 Synchrotron XRPD and GEM NPD Data (See Also Figures 1, S3 and Table S2)

atom	site	*x*	*y*	*z*	100 × *U*_iso,eq_ (Å^2^)	occupancy
Sr(1)	2h	0.5	0.5	0.1512(1)	0.43(4)	1^a^
Mn(1)	1a	0	0	0	0.20(2)	1^a^
S(1)	2g	0	0	0.2522(1)	0.66(3)	1^a^
S(2)	1d	0.5	0.5	0.5	1.27(4)	1^a^
O(1)	2f	0	0.5	0	0.87(5)	1^a^
Cu(1)	4i	0	0.5	0.3810(2)	3.36(3)^b^	0.429(1)
Cu(2)	8s	0.351(3)	0	0.4071(2)	3.36(3)^b^	0.1924(7)
Cu(3)	8r	0.192(1)	0.192(1)	0.39567(6)	3.36(3)^b^	0.0386(5)

a Not refined.

b The displacement parameters were constrained to be equal *P*4/*mmm*. *a* = 4.016345(1) Å, *c* = 11.40708(5) Å. χ = 1.887; *R*_wp_ = 3.936%.

In this compound, the coordination environment about Mn at room temperature is a highly elongated MnO_4_S_2_ octahedron containing four short equatorial Mn–O bonds in a square net with Mn–O distances of 2.008173(1) Å and two elongated axial Mn–S distances of 2.8769(6) Å. (Note that the uncertainty in the Mn–O distance is very small as it is equal to half the *a* lattice parameter which may be determined very precisely from a powder diffraction measurement). The difference between the equatorial and axial bond distances is larger than can be ascribed solely to the 0.44 Å difference in radii between the oxide and sulfide anions. This extreme anisotropy in the ligand field may be attributed to the inherent ordering of oxide and sulfide into distinct layers and the need to satisfy the coordination requirements of the intervening Sr^2+^ cations. The presence of Mn^3+^ ions with the high spin d^4^ configuration which would be Jahn–Teller active in an octahedral environment would also favor axial elongation, but comparison with structurally related compounds^6,7,10^ suggests that the anion ordering plays the dominant role in determining the elongated transition metal coordination environment in the oxide layers with the electronic configuration having a secondary role as is evident below.^11^ This point is discussed further in the Supporting Information (Figure S1 and Table S1).

### Low Temperature Crystal Structure

Variable temperature synchrotron XRPD measurements of sample 1 reveal highly anisotropic thermal contraction on cooling. From ∼190 to 150 K the *a* lattice parameter undergoes an expansion with a corresponding decrease in the *c* lattice parameter (Figure 3). The expected thermal contraction of unit cell volume is observed, though there is a slight inflection of the trend in this temperature range. The onset of this behavior is accompanied by the appearance of several additional weak reflections, some of which are unusually broad compared with the reflections present at ambient temperature (Figure S5).

**Figure 3:**
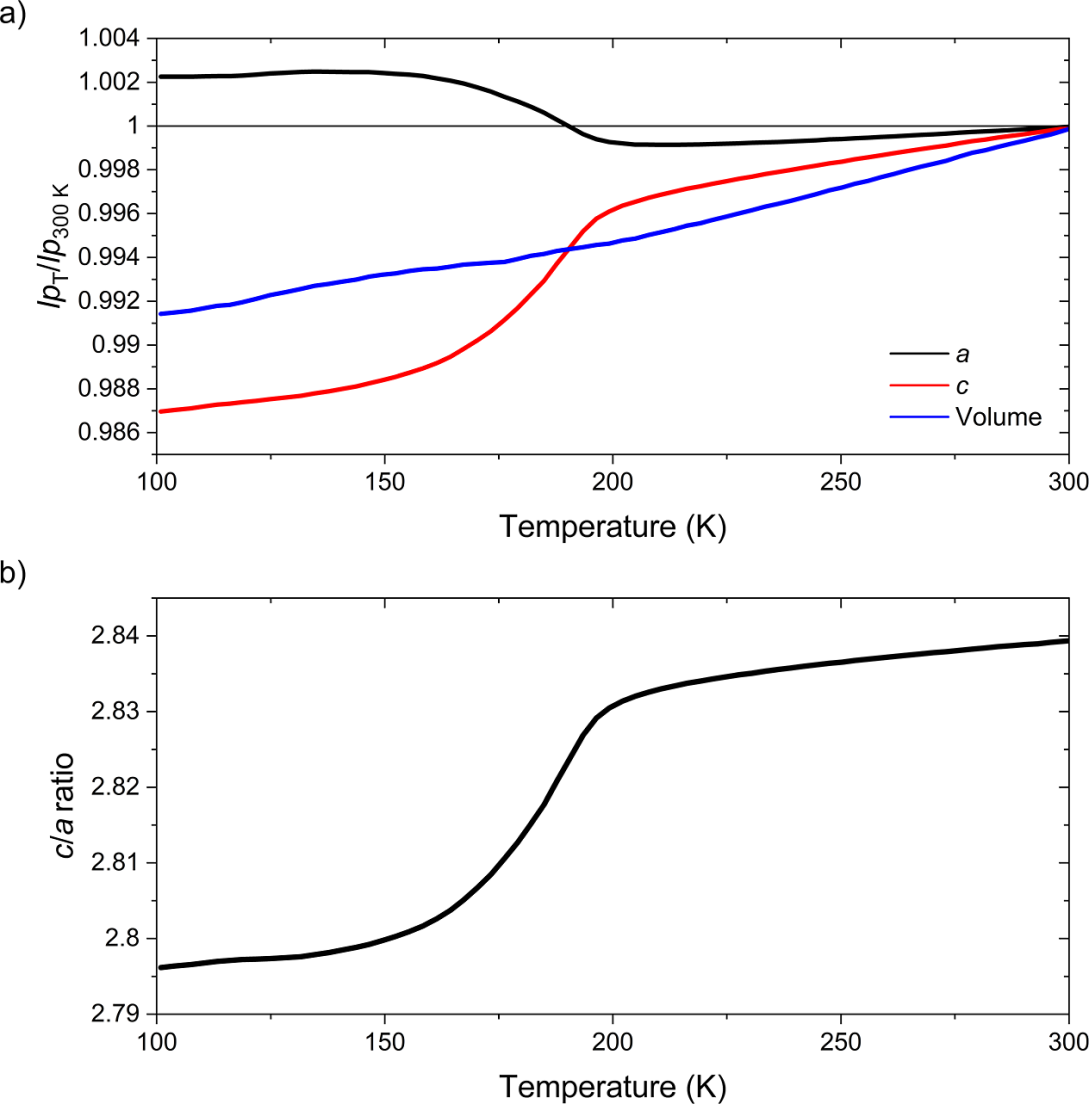
Temperature dependence of the lattice parameters (abbreviated as *lp* in (a)). The parameters were obtained by Rietveld refinements against diffraction patterns collected using the PSD detector of the I11 diffractometer (Diamond Light Source) while cooling the sample from 300 to 100 K. The parameters in (a) are normalized with respect to their values at 300 K. Error bars are within the plotted points.

Single crystal X-ray diffraction measurements of Sr_2_MnO_2_Cu_3.5_S_3_ (sample 1) performed at 100 K after rapid cooling revealed intense superstructure peaks which could be indexed on a √2*a* × √2*a* × 2*c* expansion of the room temperature unit cell (Figure 4) with lattice parameters *a* = *b* = 5.69216(15) Å, *c* = 22.4854(6) Å. The metric symmetry of the unit cell is tetragonal and the similarity in the internal agreement factor (*R*_int_) between the Laue classes 4/*mmm* (0.119) and *mmm* (0.118) as well as the lack of peak splitting in the high-resolution XRPD data does not support an orthorhombic distortion.

**Figure 4:**
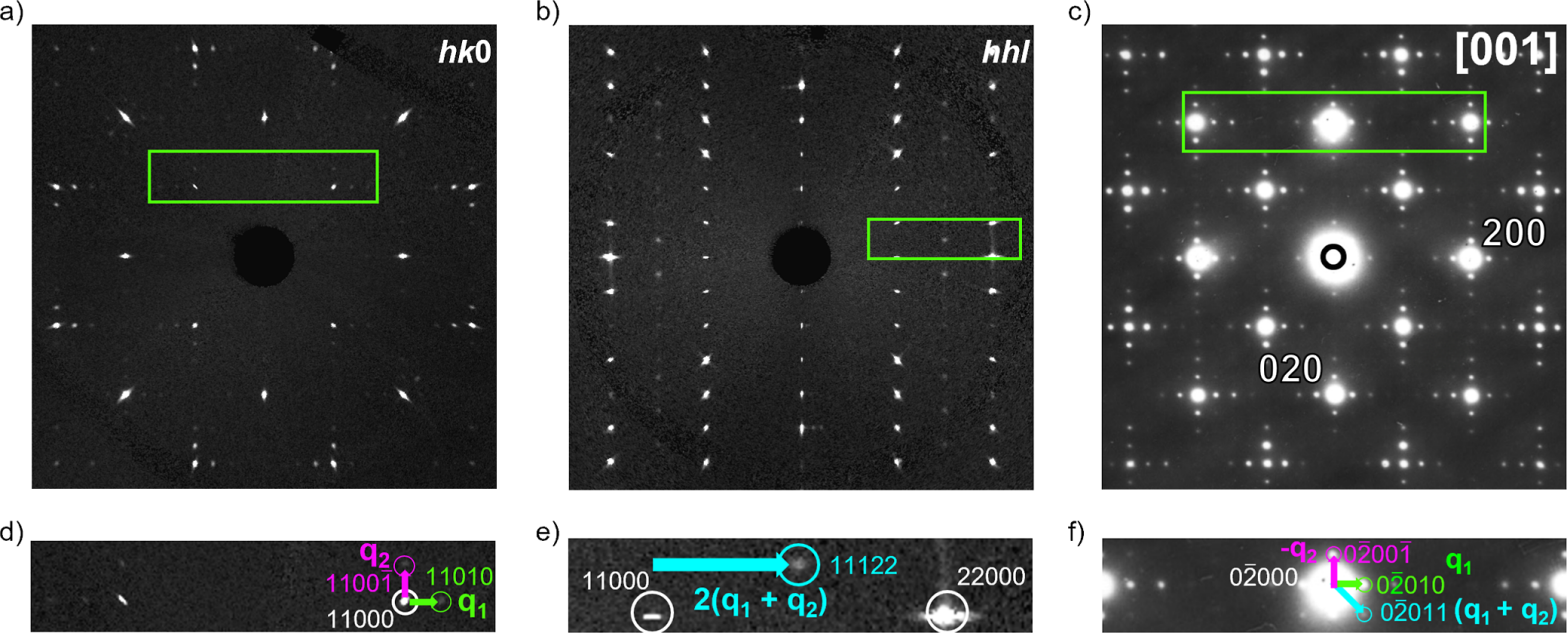
Reconstructed precession images in the (a) *hk*0 and (b) *hhl* layers from single crystal X-ray diffraction patterns collected at 100 K and (c) electron diffraction pattern of the [001] zone measured at 110 K after rapid cooling of sample 2. In each of (a–c), the green box indicates the region of the pattern which is detailed in (d–f). Here the modulation vectors are indicated by arrows. Mixed satellites are indicated by blue arrows in the *hhl* layer (e) and in the [001] zone (f) (although in the latter case, they could result from multiple diffraction, and are indeed not evident in the *hk*0 reconstructed precession image in (a)&(d)). Mixed satellites visible in the *hhl* layer can also be observed in the *hk*1 precession layer (Figure S7).

Reconstruction of the *hk*0 and *hhl* precession layers revealed two orthogonal sets of first order satellite reflections accompanying the main reflections which could be indexed with modulation vectors **q**_**1**_ = 0.2582(5)a* and **q**_**2**_ = 0.2582(5)b*. A recent report by Zhou et al. identifies an incommensurate structure for a phase with similar composition.^33^ The selection of two modulation vectors implies that the incommensurate modulation should be described by a (3 + 2)­D tetragonal superspace group. An alternative interpretation is a (3 + 1)­D superspace description, whereby the observed precession images can be explained by a single modulation vector **q**_**1**_ = 0.2582(5)a*. In that case, the orthogonal satellites are then only present due to an exchange of the a* and b* directions by a 90° twinning operation about the c* axis. A (3 + 1)­D description of the modulation would necessitate an orthorhombic distortion as tetragonal symmetry is violated by a 1D modulation along the a* or b* directions.^34^ ‘Mixed’ satellite reflections (*h*, *k*, 0, ±1, ±1) are observed in the [001] zone electron diffraction patterns collected at 110 K after rapid cooling in the original report by Gál et al.,^13^ implying the modulation has (3 + 2)­D dimensionality (Figure 4c). While it is possible that these mixed satellites in the electron diffraction pattern are observed as a result of multiple diffraction, the presence of satellite reflections in the single crystal X-ray diffraction experiment that can only be indexed by the summation of the **q**_**1**_ and **q**_**2**_ vectors in the reconstructed *hhl* layer unambiguously signals a 2D modulation and together with the metric symmetry indicate that a tetragonal (3 + 2)­D superspace group is the appropriate choice.

The reflection conditions for the main superstructure reflections derived from the reconstructed precession images are *hk*0: *h* + *k* = 2*n*, *h*00: *h* = 2*n*, *hhl*: *l* = 2n, 00*l*: *l* = 2*n*, giving the extinction symbol *Pn*—*c* and therefore *P*4_2_/*nmc* (no. 137) is the only possible tetragonal space group. The orthorhombic subgroup *Pmmn* (no. 59) was also considered in structural refinement of the average structure. Both models successfully converged and provided fits of similar statistical quality, with the tetragonal model requiring fewer parameters, so we concluded that the tetragonal model is a suitable description of the low temperature structure (Table S3 & S4). Additional reflection conditions were observed for the satellite reflections, *h*0*lm*0: *m* = 2*p*, 0*kl*0*n*: *n* = 2*p*, *hhlmn*: *m*,*n* = 2*p* corresponding to the superspace group *P*4_2_/*nmc*(*a*,0,0)­0000­(0,*a*,0)­00s0 (no. 137.2.63.4)^35–37^ (Figure S6 & S7).

The low temperature average structure was initially analyzed neglecting the modulation. The structure was solved using the charge-flipping algorithm implemented in SUPERFLIP^29^ and subsequent least-squares refinements were performed against the single crystal X-ray diffraction data in which the atomic parameters and anisotropic displacement parameters (ADPs) of all atoms were refined, as well as the occupancies of the Cu atoms. Two crystallographically inequivalent Mn sites were observed, which are arranged in a checkerboard pattern within the MnO_2_ sheets (Figure 5).

**Figure 5:**
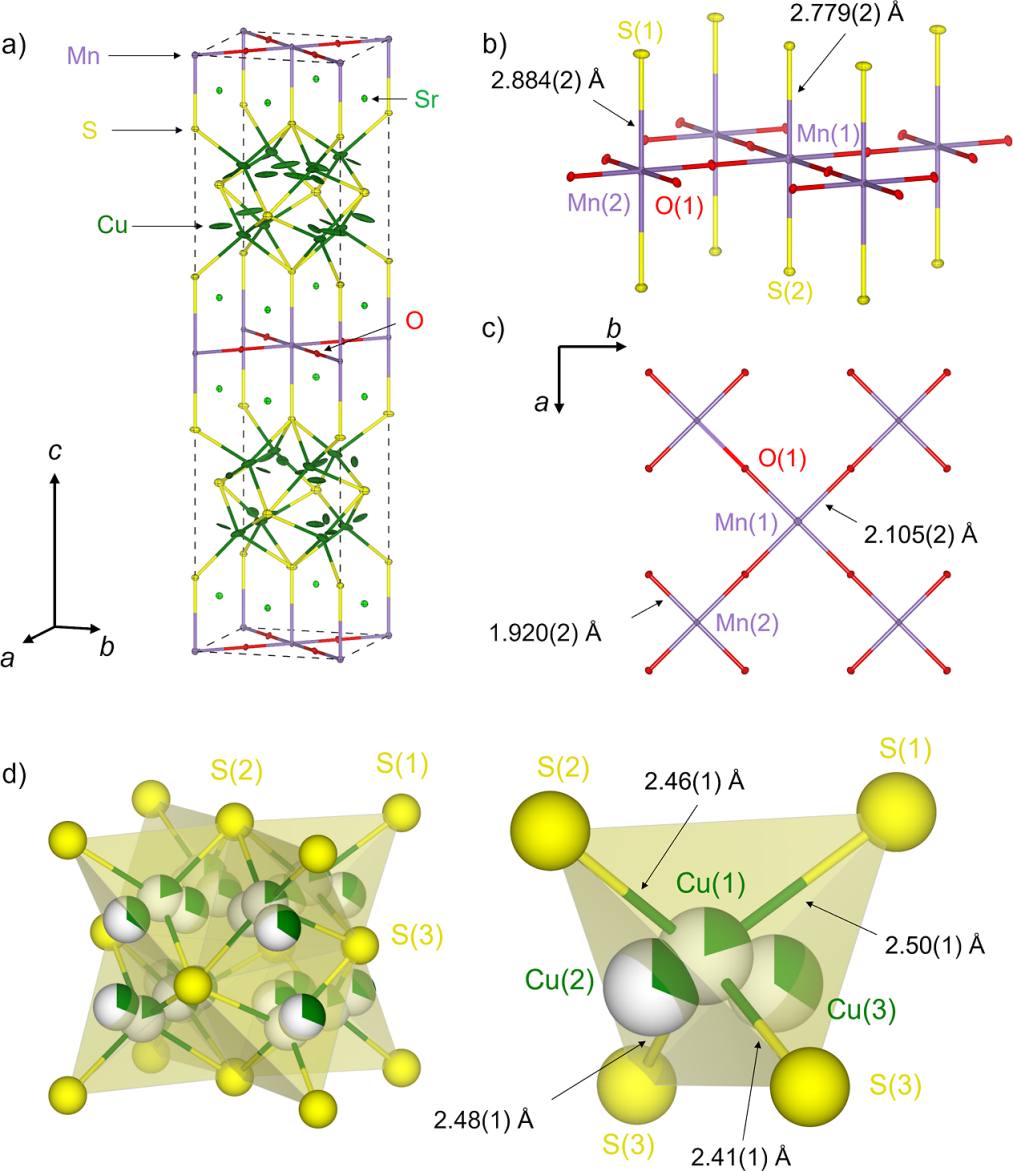
(a) Average crystal structure of Sr_2_MnO_2_Cu_3.5_S_3_ at 100 K determined by refinement against single crystal X-ray diffraction data (Table 2). The Mn coordination environment is shown in (b) and the in-plane bonding in the MnO_2_ sheets are shown in detail from the [001] direction (c). The Cu_3.5_S_3_ layer is highly disordered with much of the electron density due to copper displaced toward the triangular faces of the CuS_4_ tetrahedra. Bonds from the “ideal” tetrahedral site are shown in (d). Anisotropic displacement ellipsoids in (a–c) are shown at the 50% level. The partial filling of the spheres representing the copper atoms reflect the copper site occupancy factors.

Structural parameters and selected bond distances at low temperature are listed in Tables 2 & 3. Compared with the room-temperature structure, the Mn–S distances relax about each Mn site along the *c* direction. The oxygen atoms move toward the Mn(2) site within the MnO_2_ planes and away from the Mn(1) site in a “breathing mode” distortion which results in a 9% difference between the two Mn–O distances. This behavior is highly reminiscent of a charge order (CO) transition whereby distinct Mn^2+^ and Mn^3+^ sites become crystallographically ordered.^15^ A significant degree of disproportionation is supported by the calculated bond valence sums of +2.11 for Mn(1) and +2.93 for Mn(2) at 100 K,^38^ suggesting near complete charge order of Mn^2+^ and Mn^3+^ ions. There is no clear evidence of long-range charge-ordering in the related *m* = 1 compound, Sr_2_MnO_2_Cu_1.5_S_2_, where there is unambiguous long-range Cu/vacancy ordering below about 250 K,^8^ but only a single Mn site is present in the average structure. Electron diffraction measurements of Sr_2_MnO_2_Cu_1.5_S_2_ revealed additional weak reflections similar to those related to the charge/orbital order in La_0.5_Sr_1.5_MnO_4_ below 220 K,^39,40^ and the thermal behavior of the oxide ion displacement ellipsoids in Sr_2_MnO_2_Cu_1.5_S_2_ also suggested the possibility of CO. However, here, in Sr_2_MnO_2_Cu_3.5_S_3_, the charge ordering is much clearer.

**Table 2:** Refined Structural Parameters for Sr_2_MnO_2_Cu_3.5_S_3_ at 100 K from SCXRD (3D Average Model)

atom	site	*x*	*y*	*z*	100 × *U*_iso,eq_ (Å^2^)	occupancy
Sr(1)	4d	0.5	0	0.42057(3)	0.61(4)	1^a^
Sr(2)	4d	0.5	0	0.57323(3)	0.68(4)	1^a^
Mn(1)	2a	0.5	0.5	0.5	0.65(5)	1^a^
Mn(2)	2b	0	0	0.5	0.51(5)	1^a^
O(1)	8f	0.2385(3)	0.7615(3)	0.5	0.6(1)	1^a^
Cu(1)	16h	0.761(2)	0.276(2)	0.3100(5)	2.2(2)	0.2041(12)
Cu(2)	16h	0.697(2)	0.376(3)	0.2958(6)	2.2(2)	0.3109(14)
Cu(3)	16h	0.9197(9)	0.2041(6)	0.2929(2)	4.6(1)	0.3428(2)
S(1)	4c	0.0	0.0	0.37174(7)	0.75(7)	1^a^
S(2)	4c	0.5	0.5	0.37639(7)	1.12(8)	1^a^
S(3)	4d	0	0.5	0.23709(8)	1.39(8)	1^a^

a Not refined. *P*4_2_/*nmc* (origin choice 1 at 4̅*m*2) *a* = 5.69216(15) Å, *c* = 22.4854(6) Å

**Table 3:** Selected Bond Lengths and Interatomic Distances in Sr_2_MnO_2_Cu_3.5_S_3_ at 100 K Derived from SCXRD

atoms	distance (Å)	atoms	angle (°)
Mn(1)–O(1) [4]^a^	2.105(2)	S(1)–Cu(1)–S(2)	108.5(5)
Mn(1)–S(2) [2]	2.779(2)	S(3)–Cu(1)–S(3)	112.5(5)
Mn(2)–O(1) [4]	1.920(2)	S(3)–Cu(1)–S(1)	113.0(5)
Mn(2)–S(1) [2]	2.884(2)		
		S(3)–Cu(2)–S(2)	138.3(8)
Cu(1)–S(1) [1]	2.50(1)	S(3)–Cu(2)–S(2)	106.3(6)
Cu(1)–S(2) [1]	2.46(1)	S(3)–Cu(2)–S(3)	115.3(6)
Cu(1)–S(3) [1]	2.41(1)		
Cu(1)–S(3) [1]	2.48(1)	S(3)–Cu(3)–S(1)	99.1(2)
		S(3)–Cu(3)–S(1)	148.5(3)
S(1)–S(2) [4]	4.02632(6)	S(3)–Cu(3)–S(3)	112.0(2)
S(1)–S(3) [2]	3.754(2)		
S(1)–S(3) [2]	4.155(2)		
S(2)–S(3) [1]	3.822(2)		
S(2)–S(3) [1]	4.232(2)		
S(3)–S(3) [2]	4.0666(6)		

a Numbers in square brackets indicate the number of bonds/distances of a particular type.

The bond length distribution about Mn(2) indicates that the coordination environment becomes more distorted on cooling, with an increase of the ratio of axial (Mn–S) and equatorial (Mn–O) bond distances from 1.4326(9) at room temperature to 1.502(2) at 100 K. This is primarily driven by the movement of oxide ions between nearest neighbor Mn anions toward the higher-valence Mn^3+^ ion. Compression of the equatorial Mn(2)-O bonds and slight elongation of the axial Mn(2)-S bond distances is consistent with this site becoming purely Mn^3+^, and the Mn(2)-O distances are comparable with the average equatorial Mn–O distances of 1.9116(4) Å in Sr_2_Mn_2_O_4_Se^41^ and 1.9075(3) Å in LaMnO_3_ at ambient temperature,^42,43^ which both contain Mn^3+^ ions. The unequal occupancy of the σ-antibonding *d* states in the d^4^ ion with the high-spin (d_*xz*_)^1^(d_*yz*_)^1^(d_*xy*_)^1^(d_*z*_^2^)^1^(d_*x*_^2^_–y_^2^)^0^ configuration means that strong axial elongation for this site may be tolerated (as in a Jahn–Teller distortion for Mn^3+^). The elongation of the axial Mn–S bonds for this Mn(2) site accounts for <1% of the distortion compared with the ambient temperature structure i.e., the dominant effect is shortening of the Mn(2)-O distances, suggesting that lengthening of the Mn(2)–S(1) bond distances is restricted by the bonding requirements of the intervening Sr^2+^ ions as discussed above. In contrast, the axial bonds about Mn(1) are compressed compared with the ambient temperature structure, and this, with an increase in the Mn(1)–O bond lengths, is entirely consistent with equal occupancy of the σ-antibonding d orbitals for high-spin d^5^ Mn^2+^ cations i.e. these structural changes allow the octahedral environment of Mn(1) to become less distorted and the ratio of axial to equatorial bond distances about Mn(1) decreases to 1.320(2) at 100 K. The radius of S^2–^ is greater than O^2–^ by 0.44 Å and therefore the ideal ratio is (*d*_Mn–O_ + 0.44/*d*_Mn–O_) = 1.1973 using ionic radii for Mn^2+^, S^2–^, and O^2–^ of 0.83, 1.84, and 1.40 Å, respectively,^44^ so the site is still axially elongated due to the nature of the crystal structure, but this result suggests that the electron configuration also plays a role as is evident in structurally related compounds.^7,10,11^

The increase in the Mn(1)–O distances is compensated by the decrease in the Mn(2)–O bond distance and therefore the changes in the *d*_*x* ^2^–*y* ^2^_ energy levels from room temperature are equal and opposite. The Mn(1)–S(2) bond distance changes by a greater magnitude than the Mn(2)–S(1) bond distance and thus the decrease in the Mn(2) d_*z*_^2^ orbital energy via the slight axial elongation is much smaller than the corresponding increase for Mn(1) due to axial compression and the situation is as in the cartoon in Figure 6.

**Figure 6:**
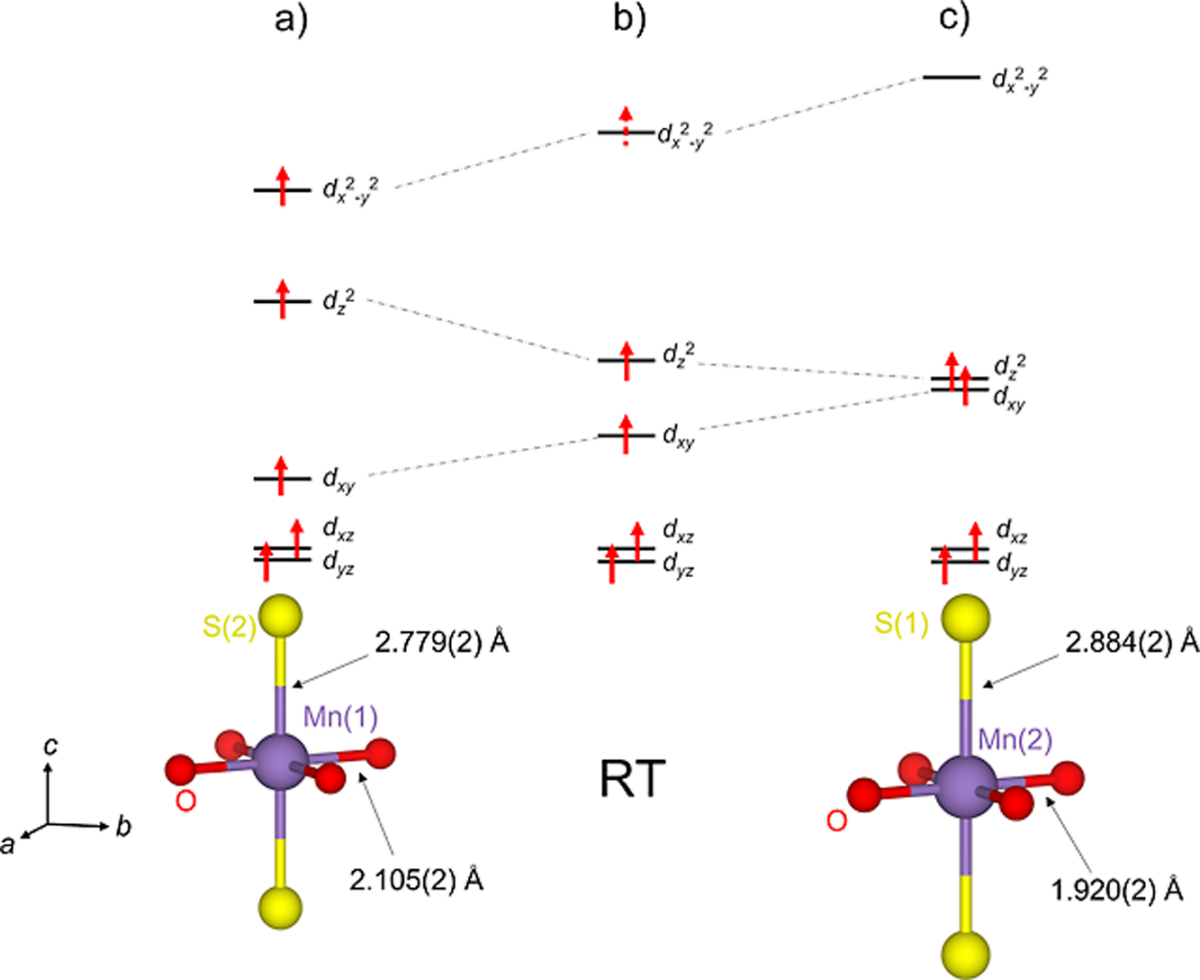
Cartoons showing the d-orbital splitting for the high-spin Mn environments in Sr_2_MnO_2_Cu_3.5_S_3_ ranging from the less axially elongated Mn^2+^ Mn(1) octahedron (a) to the highly elongated Mn^3+^ Mn(2) octahedron (c) via the mixed-valent room temperature octahedron (b). The dotted arrow in (b) reflects the mixed-valency of the Mn site at room temperature.

Temperature dependent measurements on sample 1 were made on cooling the sample using the ID22 beamline to probe the thermal evolution of the Mn coordination environments. Figure 7a shows the refined Mn–O and Mn–S bond distances derived from Rietveld refinements. The bond valence sums at low temperatures reach +2.13 and +2.95 for Mn(1) and Mn(2), respectively, at 4 K suggesting that the charge order nears completion. Similar analysis for sample 2 which was measured on warming from 6 K using the GEM instrument yielded comparable values (Figure S8a and Table S5).

**Figure 7:**
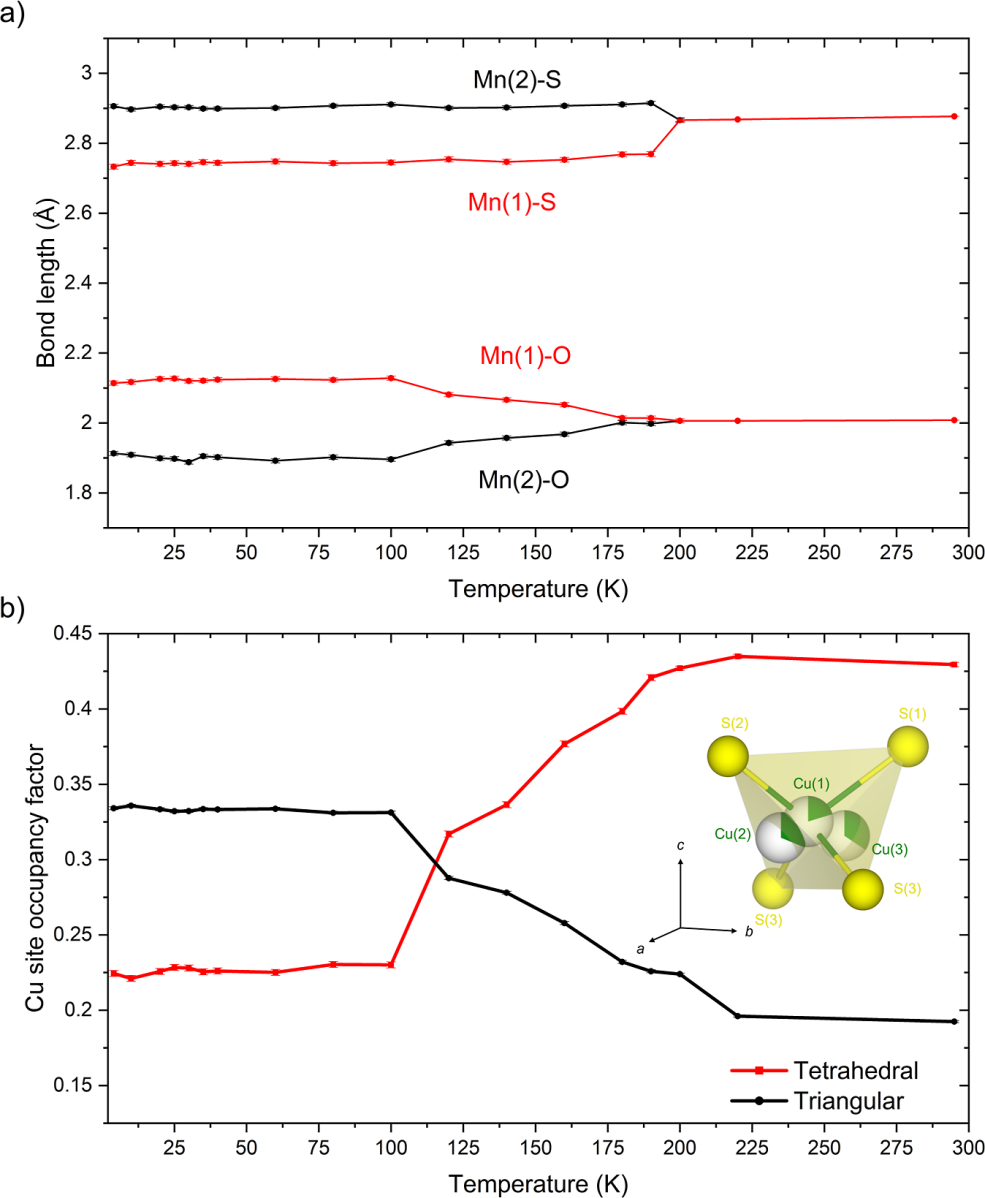
Thermal evolution of (a) Mn bond distances and (b) fractional occupancy of the tetrahedral and triangular copper sites in Sr_2_MnO_2_Cu_3.5_S_3_ (sample 1) from refinement against ID22 data using the CO model in space group *P*4_2_/*nmc* below 200 K and the disordered *P*4/*mmm* model above 200 K. The partial filling of the spheres representing the copper atoms reflects the copper site occupancy factors at the lowest temperature.

Significant tilting distortions of the CuS_4_ tetrahedra accompany the changes within the MnO_2_ planes, indicated by the four differing Cu–S distances between the ideal site at the center of the tetrahedron and the vertices of the tetrahedron and S–Cu–S bond angles which diverge further from the ideal value of 109.5° relative to ambient temperature. The sulfur atoms of the MnO_4_S_2_ octahedra, also form vertices of the CuS_4_ tetrahedra and the relaxation of the Mn–S distances accompanied by the charge ordering directly influences the bonding within the antifluorite-type sulfide layers. The competition for the sulfur atoms from the Mn sites within the MnO_2_ planes results in the lengthening of the vertical height of the tetrahedra as the Mn(1)–S(2) distance contracts by 3.45% while the increase in the Mn(2)–S(1) distance, which counteracts this, is only 0.17%. The S–S distance which defines the base of the CuS_4_ tetrahedron is equal to *a*_CO_/√2 and lengthens by 0.3% on cooling between room temperature and 100 K. The displacement of the S(3) atom, which is the origin of the tilting, offsets these effects resulting in a net decrease of the separation between the MnO_2_ planes and the sulfur atoms at the center of the Cu_3.5_S_3_ slab by 2.27%. The net effect of these structural changes is an increase in the volume of CuS_4_ tetrahedra. Accordingly, the average Cu–S distance increases by 0.3% in spite of the volume of the unit cell decreasing across the measured temperature range. Consistent with our previous report, the response of the copper ions to the increased size of the tetrahedra is to move toward the less highly coordinated triangular sites at the faces of the tetrahedra.^13^ The site occupancies of the triangular sites are found to increase at the expense of the tetrahedral site. The thermal evolution of the copper site preference with temperature was determined by Rietveld refinement against data collected for sample 1 using the ID22 beamline (Figure 7b). Similar results were obtained for sample 2 measured on the GEM instrument (Figure S8b). In these refinements, suitable constraints were initially applied to ensure equal occupancy of the crystallographically distinct but chemically similar Cu(2) and Cu(3) triangular sites and a further constraint ensured that the correct stoichiometry was preserved. The site occupancy factor for these sites at the faces of the CuS_4_ tetrahedra increases significantly across the temperature range at the expense of the fractional occupancy at the center of the tetrahedral site on further cooling, consistent with an increase in the distortions within the MnO_2_ plane (Figure 7b). Occupancy of the copper site at the center of the tetrahedra (Cu(1)) reaches a minimum value approaching 20% at 4 K, indicating that the displacements of the copper ions toward the triangular faces of the tetrahedra is not complete even at the lowest temperatures. Subsequent refinements without the occupancy constraints on the two triangular sites did not improve the visual or statistical quality of the fit and revealed approximately equal occupation of the Cu(2) and Cu(3) sites suggesting that the assumption to equate their occupancy factors in the initial stages of the refinement was reasonable.

Within the context of mixed-valent manganites, the segregation of differently charged ions to different sublattices disfavors the double exchange mechanism, which is the origin of the metallicity in these systems, via localization of electrons in σ-antibonding orbitals. Therefore, in many cases the onset of CO is associated with an increase in the electrical resistivity.^17^ Comparable behavior is observed in Sr_2_MnO_2_Cu_3.5_S_3_ below 190 K, suggesting that charge transport is influenced by the structural transition supporting our conjecture above (Figure 8). Several reports indicate the highly hysteretic behavior of the electrical resistivity in response to the CO transition in perovskite manganites and qualitatively similar results are obtained on measurement of the electrical resistivity of pressed pellets of Sr_2_MnO_2_Cu_3.5_S_3_ (Figure S9).

**Figure 8:**
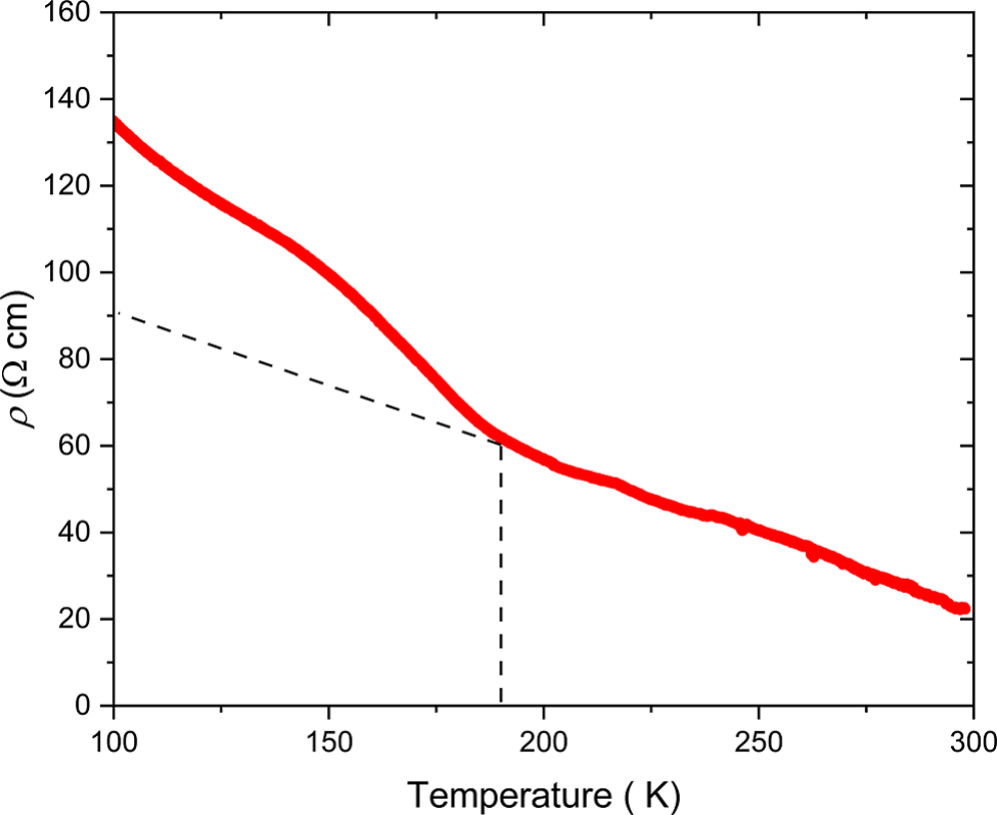
Temperature variation of electrical resistivity of a pressed pellet of Sr_2_MnO_2_Cu_3.5_S_3_ showing a marked increase at the CO transition evident in the structural measurements. Note that the possibility of preferred orientation of the grains was not assessed in this experiment. Transport measurements on sufficiently large aligned single crystals are a target of future research.

### Incommensurate Modulation of the Crystal Structure

The low-temperature structural model discussed above neglected the satellite reflections in the single crystal X-ray measurements arising from the incommensurate modulation of the structure, and thus represents the 3D average structure. The model revealed significantly elongated anisotropic displacement ellipsoids of the copper ions suggesting a disorder of these sites. It is plausible that periodic modulations of the copper positions are incommensurate with the parent superstructure and that this is the source of the satellite reflections. The structure therefore requires the use of the superspace formalism to be fully accounted for.^45^ In incommensurately modulated structures, the structural parameters of atoms (displacement, site occupancy factor, anisotropic displacement parameter etc.) deviate from their values in the basic unit cell in 3D physical space as a function of the periodic modulation function *u̅*(*x̅*_4_,*x̅*_5_). The fact that the modulation function is periodic ensures that the translational symmetry which is not present in physical 3D space, is recovered in superspace. Initially two atomic displacive waves were added for each atom using a model in superspace group *P*4_2_/*nmc*(a,0,0)­0000­(0,a,0)­00s0. Additional site occupancy modulations of the Cu ions were introduced until no further improvement to the statistical quality factor for the fit was observed. In total, four additional harmonic waves describing the occupancy modulation of each Cu site were added to the refinement. The addition of a modulation wave for the anisotropic displacement parameters of all atoms further improved the statistical quality of the fit. All modulation parameters for this incommensurately modulated (3 + 2)­D space model are given in Tables S6–S8 and the modulations are shown in Figure 9. A schematic representation of the copper sulfide layer within a 4 × *a* approximant of the modulated structure is shown in Figure 10 and the refined parameters from SCXRD analysis are shown in Table 4.

**Figure 9:**
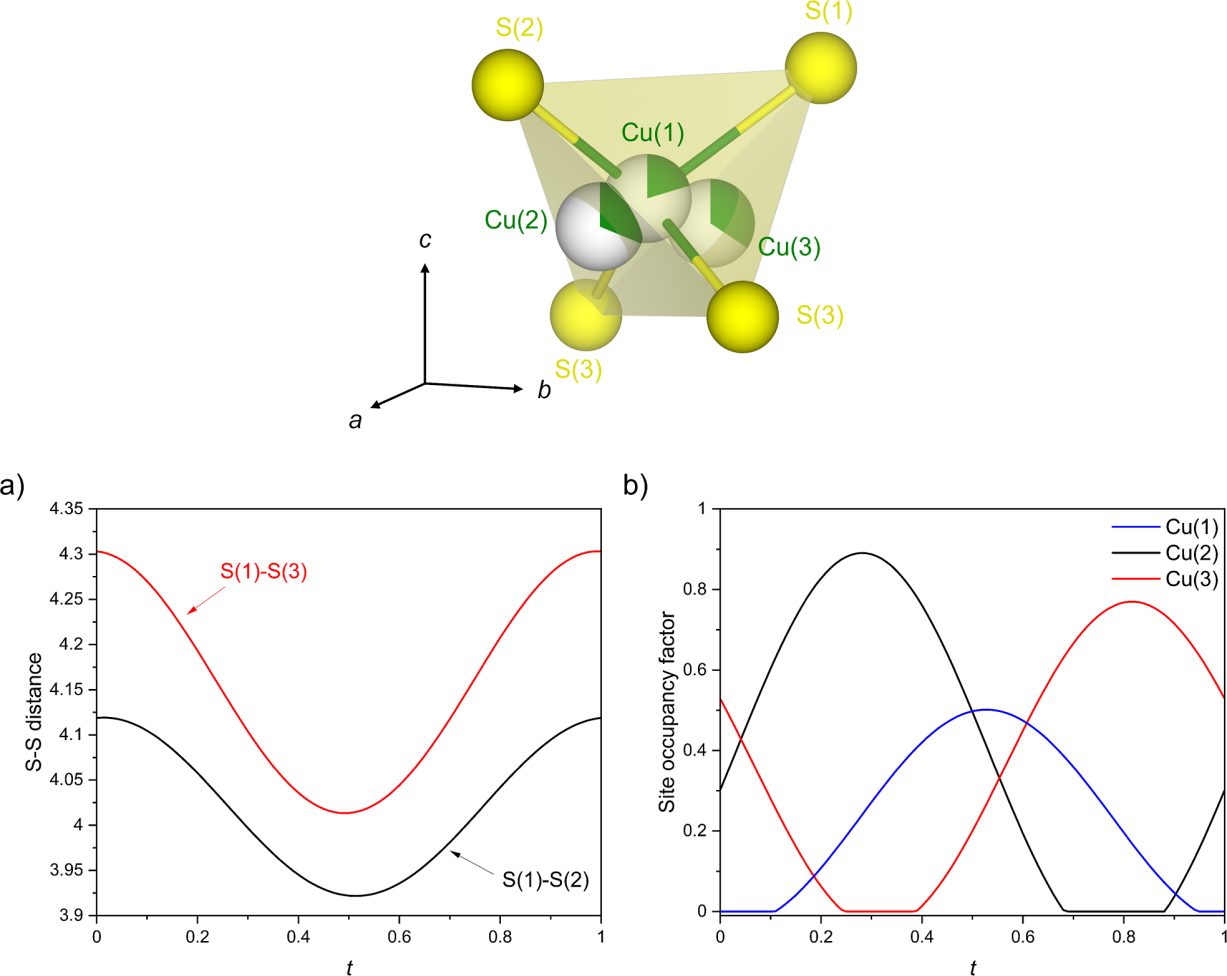
*t*-plots of the (a) S–S distances and (b) copper site occupancy factors indicating that the site occupancy factor of the Cu(1) site (center of the tetrahedron) is highest when the volume of the CuS_4_ tetrahedron reaches a minimum. A CuS_4_ tetrahedron with atom labels indicated is shown above. See also Figures S11–S15.

**Figure 10:**
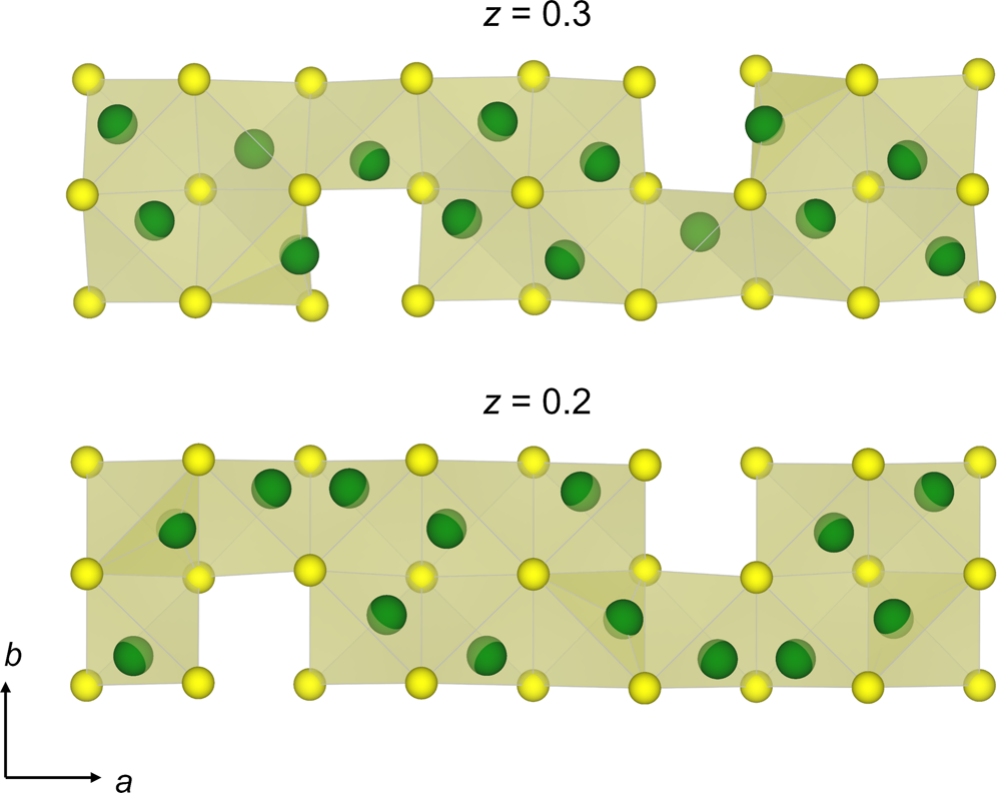
A schematic representation of the copper sulfide layer centered at *z* = 0.25 within a 4 × *a* approximant of the modulated crystal structure of Sr_2_MnO_2_Cu_3.5_S_3_ through five-dimensional space for the *t* = 0, *u* = 0 section, projected along the [001] direction. An occupancy threshold of 50% is applied to the copper positions. Copper atoms in green, sulfur atoms in yellow.

**Table 4:** Single Crystal Data Collection and Refinement Details at 100 K

crystal data	
chemical formula	Sr_2_MnO_2_Cu_3.431_S_3_
*M*_r_	576.4
crystal system, space group	tetragonal, *P*4_2_/*nmc* ^a^
superspace group	*P*4_2_/*nmc*(a,0,0)0000(0,a,0)00s0
temperature (K)	100
wave vectors	**q**_1_ = 0.2582(5)a*; **q**_2_ = 0.2582(5)b*
*a*, *c* (Å)	5.69216(15), 22.4854(6)
*V* (Å^3^)	728.54(3)
*Z*	4
radiation type	Mo *K*α
μ (mm^–1^)	26.82
crystal size (mm)	0.17 × 0.16 × 0.03
Data collection	
diffractometer	XtaLAB Synergy R, DW system, HyPix-Arc 150
absorption correction	multiscan
	CrysAlis PRO 1.171.43.130a (Rigaku Oxford Diffraction, 2024) Empirical absorption correction using spherical harmonics, implemented in SCALE3 ABSPACK scaling algorithm.
*T*_min_, *T*_max_	0.182, 1
no. of measured, independent and observed [*I* > 3σ(*I*)] reflections	110066, 2029, 1301
*R*_int_	0.154
(sin θ/λ)_max_ (Å^–1^)	0.651
Refinement	
*R*[*F* ^2^ > 2σ(*F* ^2^)], *wR*(*F* ^2^), *S* (all)	0.045, 0.104, 1.62
*R*[*F* ^2^ > 2σ(*F* ^2^)], *wR*(*F* ^2^) (main)	0.040, 0.105
*R*[*F* ^2^ > 2σ(*F* ^2^)], *wR*(*F* ^2^) (satellites)	0.056, 0.089
no. of reflections	2029
no. of parameters	175
Δρ_max_, Δρ_min_ (e Å^–3^)	3.60, −1.82

a Symmetry operations: (1) *x*_1_, *x*_2_, *x*_3_, *x*_4_, *x*_5_; (2) −*x*_1_, −*x*_2_, *x*_3_, −*x*_4_, −*x*_5_; (3) – *x*_2_ + 1/2, *x*_1_ + 1/2, *x*_3_ + 1/2, −*x*_5_, *x*_4_; (4) *x*_2_ + 1/2, −*x*_1_ + 1/2, *x*_3_ + 1/2, *x*_5_, −*x*_4_; (5) −*x*_1_ + 1/2, *x*_2_ + 1/2, −*x*_3_ + 1/2, −*x*_4_ + 1/2, *x*_5_+ 1/2; (6) *x*_1_+1/2, −*x*_2_+1/2, −*x*_3_ + 1/2, *x*_4_ + 1/2, −*x*_5_ + 1/2; (7) *x*_2_, *x*_1_, −*x*_3_, *x*_5_ + 1/2, *x*_4_ + 1/2; (8) −*x*_2_, −*x*_1_, −*x*_3_, −*x*_5_+1/2, −*x*_4_ + 1/2; (9) −*x*_1_ + 1/2, −*x*_2_ + 1/2, −*x*_3_ + 1/2, −*x*_4_, −*x*_5_; (10) *x*_1_ + 1/2, *x*_2_ + 1/2, −*x*_3_+1/2, *x*_4_, *x*_5_; (11) *x*_2_, −*x*_1_, −*x*_3_, *x*_5_, −*x*_4_; (12) −*x*_2_, *x*_1_, −*x*_3_, −*x*_5_, *x*_4_; (13) *x*_1_, −*x*_2_, *x*_3_, *x*_4_ + 1/2, −*x*_5_ + 1/2; (14) −*x*_1_, *x*_2_, *x*_3_, −*x*_4_ + 1/2, *x*_5_ + 1/2; (15) −*x*_2_ + 1/2, −*x*_1_ + 1/2, *x*_3_ + 1/2, −*x*_5_ + 1/2, −*x*_4_ + 1/2; (16) *x*_2_ + 1/2, *x*_1_ + 1/2, *x*_3_ + 1/2, *x*_5_ + 1/2, *x*_4_ + 1/2.

The internal coordinates, *x̅*_4_ and *x̅*_5_, defining the magnitude and direction of the modulation function, *u̅*(*x̅*_4_,*x̅*_5_), are defined as

$$x_{4}=t+q_{1}\cdot x\;\text{and}\;x_{5}=u+q_{2}\cdot x,\left(q_{1}=0.2582\left(5\right)a^{*};\;q_{2}=0.2582\left(5\right)b^{*}\right)$$

Where *t* and *u* are real numbers in the interval 0 ≤ *t*(*u*) ≤ 1 describing the phase of the modulation function and x̅ is the value of the specific structural parameter of the atom in the basic cell.^45^ Spanning *t* or *u* produces all possible values of the structural parameter in superspace. Thus, it is practical to describe the modulation of a specific structural parameter as a function of *t*(*u*) in *t*(*u*)-plots or as a function of both parameters in so-called *t*–*u* plots. Each point on these plots represents the value of the parameter in a single unit cell in real space. Some structural parameters that can be plotted include magnitudes of displacement, distances between atoms and site occupancy factors.

As expected from the anisotropic displacement ellipsoids refined in the 3D average structure, the S positions show displacements within the *ab* plane in the modulated structure resulting in subtle displacements of the Sr atoms to minimize under-bonding of the sulfide ions (Figures S10–S12). This is likely coupled to the significant modulation of the site occupancy factors of the copper atoms. The harmonic functions for Cu(2) and Cu(3) gave a negative occupancy of about 5% in the *t*–*u*-plots, indicating that a discontinuous function may be more suitable to describe the site occupancy modulation, however, step functions are not yet implemented in (3 + 2)­D space using the software available.^27^ Improvement in the reliability factors was observed when the occupancies, *o*_Cu_, of all Cu atoms were constrained to the interval 0 ≤ *o*_Cu_ ≤ 1.

In general, the *t*–*u* plots calculated for atoms within the copper sulfide layer centered at *z* = 0.25 show pronounced sinusoidal variation of the observed electron density as a function of *t* with little amplitude as a function of *u*, corresponding to modulation of the occupancy along the *a* direction in physical space (Figure S13 & S14). Atoms which are related by the 4_2_ screw-axis show the opposite behavior, i.e., the electron density of the copper atoms within the copper-sulfide layer centered at *z* = 0.75 (see Figure 5a) exhibit significant variations as a function of *u* (but not *t*) and therefore exhibit modulation along the *b* direction (Figure S15). For the copper sulfide layer centered at *z* = 0.25, we show the *t*-plots of the site occupancy factors of the Cu atoms in Figure 9b. The *t*-plots indicate that the maximum occupancy of the Cu(2) site occurs at values of *t* when the occupancy of Cu(3) is minimized. Since each point on the *t*-plot represents a unit cell in physical 3D space, this is consistent with the chemically reasonable alternation of the occupied triangular face within the copper sulfide slab centered at *z* = 0.25 as represented schematically in Figure 10. The sulfur atoms exhibit significant displacive modulations as a function of *t* (Figure S11) which results in the decrease of both S–S separations in the range 0 ≤ *t* ≤ 0.5 with a more marked contraction of the S(1)–S(3) distance relative to the shortening of the S(1)–S(2) distance (Figure 9a). Comparison of the *t*-plots of the copper site occupancies to those of the S–S distances allows the property to be derived, that the maximum occupancy of the copper site at the center of the tetrahedra (Cu(1)) occurs when the volume of the CuS_4_ tetrahedra is minimized by the displacements of the sulfur atoms (Figure S16). This behavior is consistent with the trend reported for the copper sulfide layers in the series of compounds Sr_2_MnO_2_Cu_2*m*–δ_S_*m*+1_ (*m* = 1 – 3)^13^.

The modulated model derived from single-crystal XRD analysis is found to account for all the additional reflections observed in the NPD and synchrotron XRPD data at low temperatures. A combined refinement was performed against data collected for sample 1 using the ID22 beamline and the GEM instrument. The broad superstructure peaks which appear below *T*_CO_ do not coincide with the sharper superlattice peaks which are indexed using the propagation vector (1/2 1/2 1/2), indicating that the overall superstructure is incommensurate (Figure S17 shows the attempted refinements without the modulation included). These broad satellite peaks could be accounted for satisfactorily using the modulation vectors determined from the SCXRD data (Figure 11). This behavior is reproducible from sample to sample, and the additional reflections in synchrotron PXRD measurements of sample 3 can also be accounted for using the superspace model described above (Figure S18).

**Figure 11:**
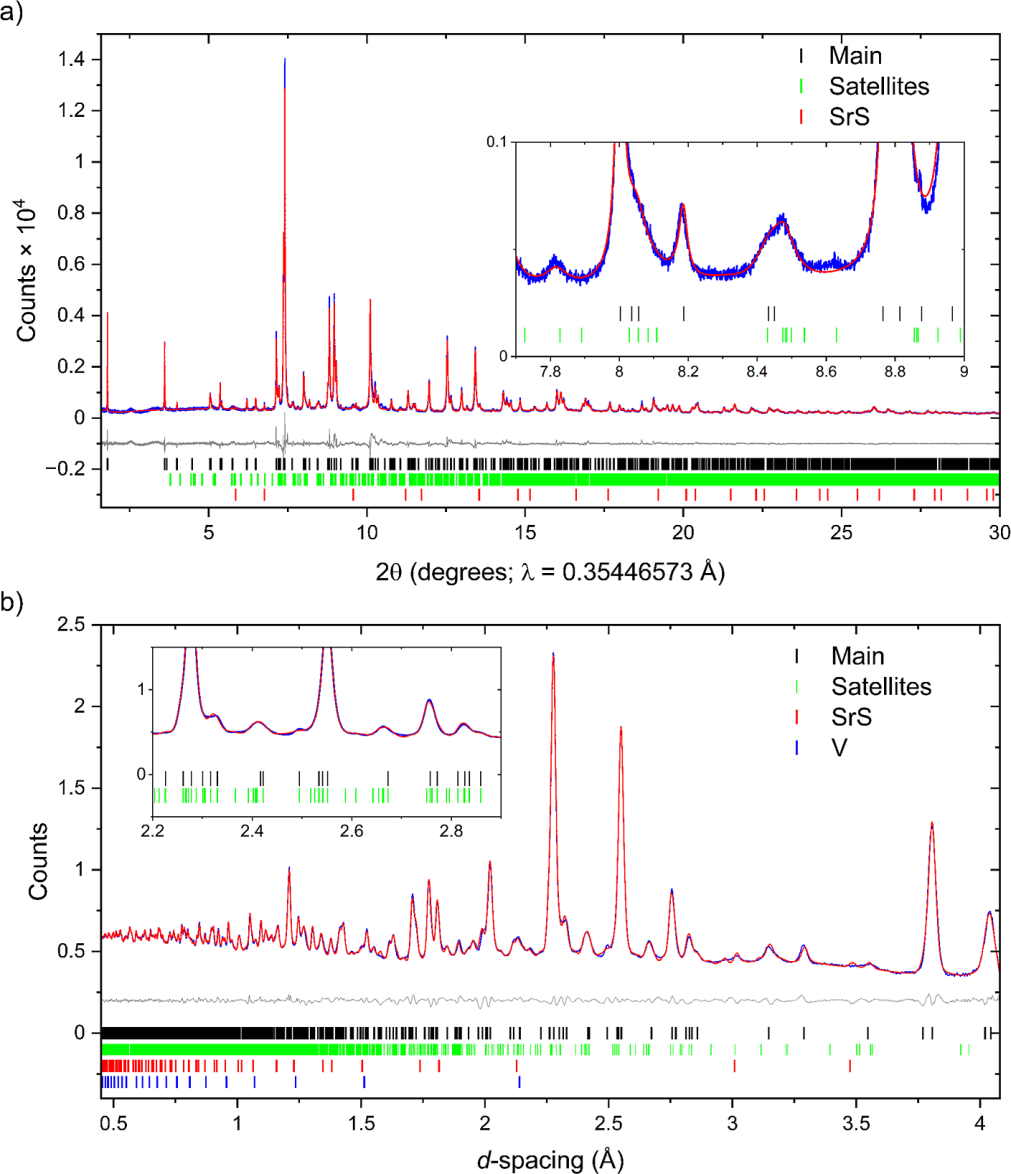
Rietveld refinements of the crystal structure of Sr_2_MnO_2_Cu_3.5_S_3_ (sample 1) against (a) synchrotron XRPD data (ID22) and (b) NPD data of bank 4 (63.62°) of the GEM instrument collected at 100 K from refinements using the modulated structure derived from SCXRD analysis. The insets show the regions of highest satellite intensity. In each case the reflections from the average structure are indicated by the black tick marks and the satellite reflections with green tick marks. The refinements without the modulation applied are unsatisfactory (Figure S17).

### Local Structure

We probed the short-range structural features of Sr_2_MnO_2_Cu_3.5_S_3_ using X-ray and neutron total scattering measurements at temperatures above and below the long-range structural transition. The temperature dependent variation in the neutron and X-ray *D*(*r*) functions are shown in Figure 12. The high real-space resolution of the neutron PDF data, along with the negative neutron scattering length of Mn, give exquisite detail on the nearest Mn–O distances. The distances in the range 1.8–2.2 Å are dominated by nearest neighbor Mn–O distances. The divergence of the Mn–O distances which is characteristic of the CO state, appears to persist locally throughout the measured temperature range, on the basis of the peak asymmetry of the Mn–O distance at 2 Å. The correlation length of this local distortion decreases on warming to room temperature as the two distinct Mn–O distances begin to coalesce in the 293 K neutron PDF. It is expected that the local distortion disappears entirely in the high-temperature charge disordered state and subtle differences which are observed in the X-ray PDFs between 293 and 400 K may indicate this. The X-ray PDFs show poor sensitivity to pair correlations between Mn and O due to the weak scattering power of O atoms, but there are clear discontinuities in both the X-ray and neutron *D*(*r*) functions between 200 and 100 K consistent with the long-range structural transition which occurs at 190 K signaled by the appearance of superstructure reflections in the Bragg diffraction data. The scenario is not dissimilar to conventional order/disorder transitions observed, for example, in Cu_3_Au where the distortions present in the ordered state persist locally in the disordered state. The result is that the low-*r* features of the PDF are unchanged through the long-range structural transition.^46,47^

**Figure 12:**
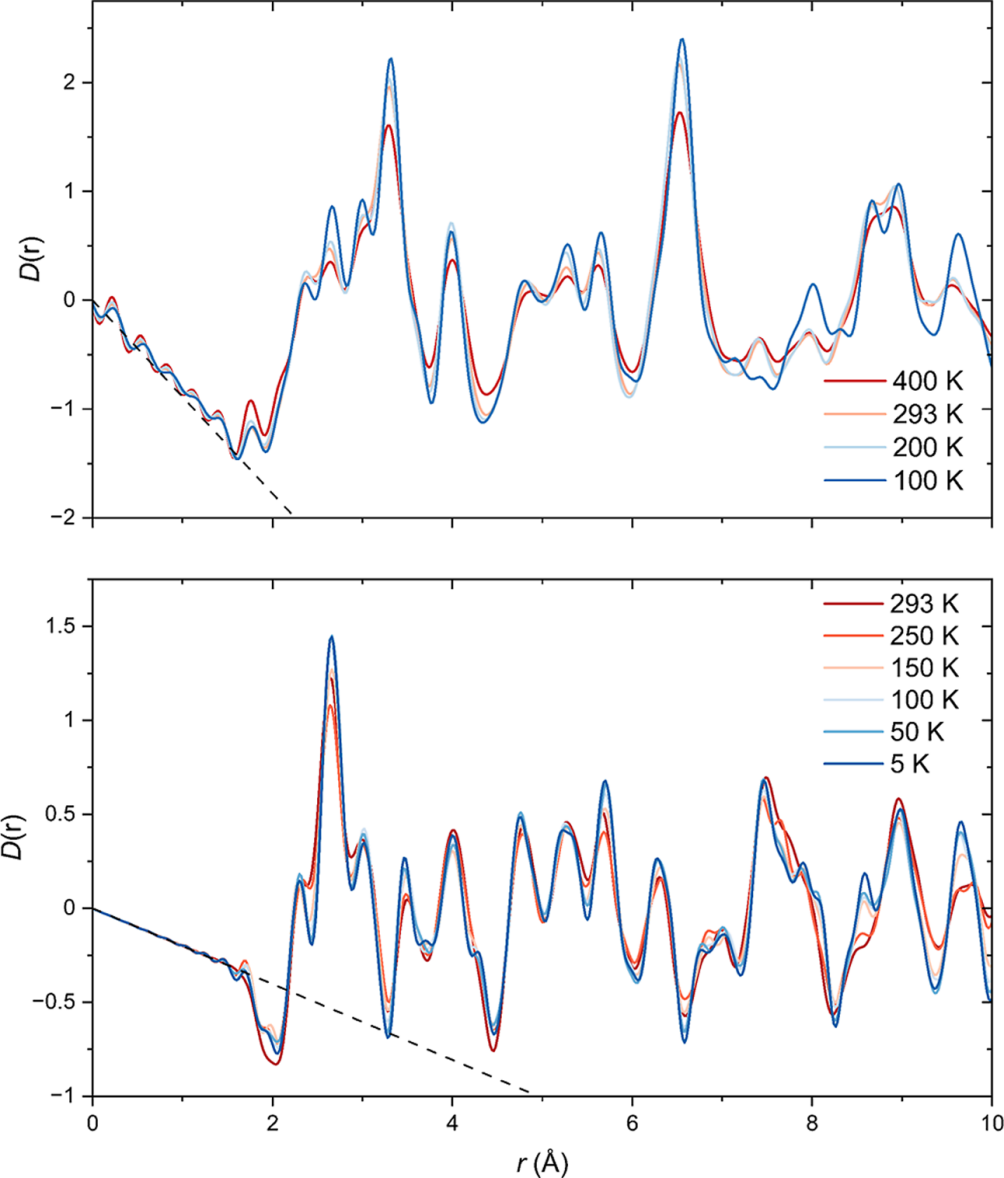
Temperature varation of (top) X-ray and (bottom) neutron PDFs. In both plots the baseline is indicated by the black dashed line.

At the highest temperature (400 K), the X-ray PDF data can be satisfactorily fit using the disordered room temperature model in space group *P*4/*mmm* at all values of *r* indicating no deviations from the average structure (Figure 13). Selected bond lengths are provided in Table 5.

**Figure 13:**
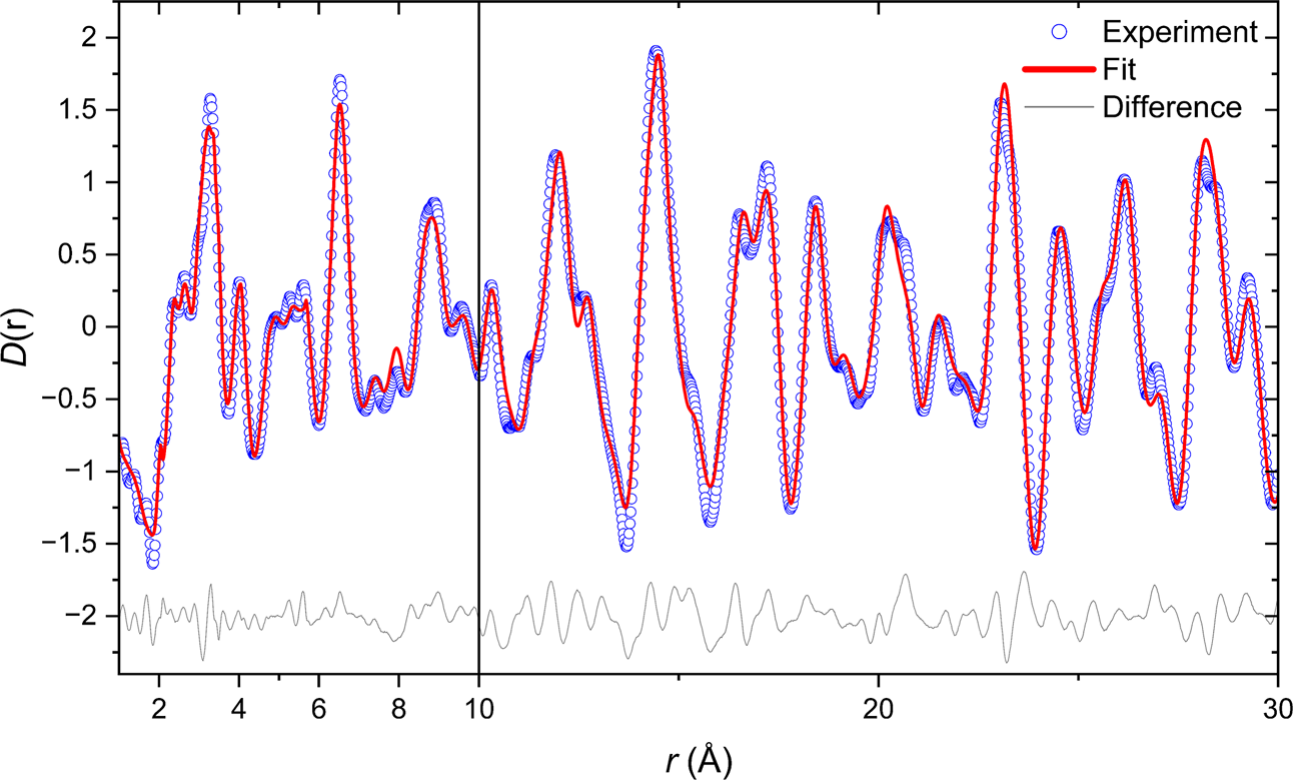
X-ray PDF fit for data collected at 400 K using the room temperature disordered model in space group *P*4/*mmm*.

**Table 5:** Metal-Anion Bond Distances in Å Obtained from PDF Fitting at Various Temperatures

	temperature (K)			
atoms	400^b^	250	100^c^	50
Mn(1)–O(1) [4]^a^	2.0142(3)	2.081(7)	2.117(7)	2.097(2)
Mn(2)–O(1) [4]	-	1.932(7)	1.905(7)	1.931(2)
Mn(1)–S(2) [2]	2.876(9)	2.72(4)	2.66(1)	2.765(9)
Mn(2)–S(1) [2]	-	2.84(1)	2.98(1)	2.88(2)
<Cu(1)-S> [4]	2.464(5)	2.51(2)	2.50(1)	2.481(6)

a The numbers in square brackets indicate the number of bonds of a particular type.

b The PDF at 400 K is adequately fitted using the room temperature disordered model in space group *P*4/*mmm* in which only a single Mn site is present.

c These bond distances are obtained from fitting of the X-ray PDF collected at 100 K.

To understand the nature of the short-range correlations within the charge ordered state in Sr_2_MnO_2_Cu_3.5_S_3_, we begin by modeling the neutron PDF data obtained at 50 K and the X-ray PDF data obtained at 100 K using the charge-ordered structure in space group *P*4_2_/*nmc* at distances up to 30 Å corresponding to the distance across 5–6 MnO_4_ square planes, allowing both local and medium range correlations to be taken into consideration. Below 190 K, the charge ordered model accounts for both the short-range and medium range features of the neutron *D*(*r*) in agreement with the structure refined from Bragg diffraction (Figure 14). The bond distances agree quantitatively with the CO model (Tables 3 and 5).

**Figure 14:**
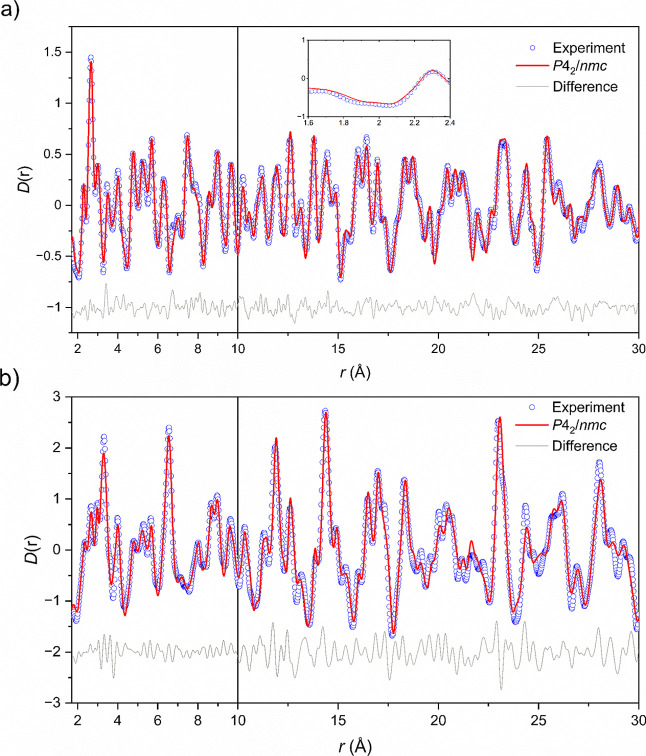
(a) Neutron PDF fit for data collected at 50 K. The inset details the region between 1.8 and 2.2 Å which is dominated by the Mn–O separations and (b) X-ray PDF fit for data collected at 100 K.

Small-box fitting of the PDF collected at 250 K using the room temperature structural model in space group *P*4/*mmm* provided a satisfactory fit at long-range but failed to account for the asymmetry of the nearest neighbor Mn–O correlations and the distances at 3 Å which corresponds to Mn–S and Sr–S correlations. Distinct Mn–O distances are precluded by the room temperature *P*4/*mmm* structure and PDFs calculated using this model produce a single peak for nearest neighbor Mn–O distances rather than the split or broadened peaks observed in the neutron PDFs at all measured temperatures (i.e., up to 250 K) indicating short-range distortions within the MnO_2_ square planes, which resemble the low temperature charge-ordered structure, are present at 250 K. Furthermore, the divergence of Sr–S distances is expected in the CO state due to the relaxation of the Mn–S distances along the *c* axis. Short-to-long-range fitting was necessary to fully account for the PDF at 250 K. Here, contributions from the long-range average structure are damped at short-range, while an ordered structural model is introduced to account for any local correlations at low *r*.^48,49^ Such short-to-long-range refinements incorporating the charge-ordered structural model at short *r* correctly capture the Mn–O and Sr–S correlations at shorter distances 1 ≤ *r* ≤ 6 Å producing an improved visual fit with *R*_wp_ = 2.90% (Figure 15). The refined domain size of the charge-ordered state is ∼12 Å at 250 K, corresponding to approximately two unit cells along the basal directions. Selected bond lengths are provided in Table 5 and show distinct Mn–O distances. We note here that since the modulation described above mainly affects the Cu occupancies, we use the basic √2*a* × √2*a* × 2*c* expansion of the ambient temperature cell in space group *P*4_2_/*nmc*, without the modulation, to model the PDF data satisfactorily at low *r*.

**Figure 15:**
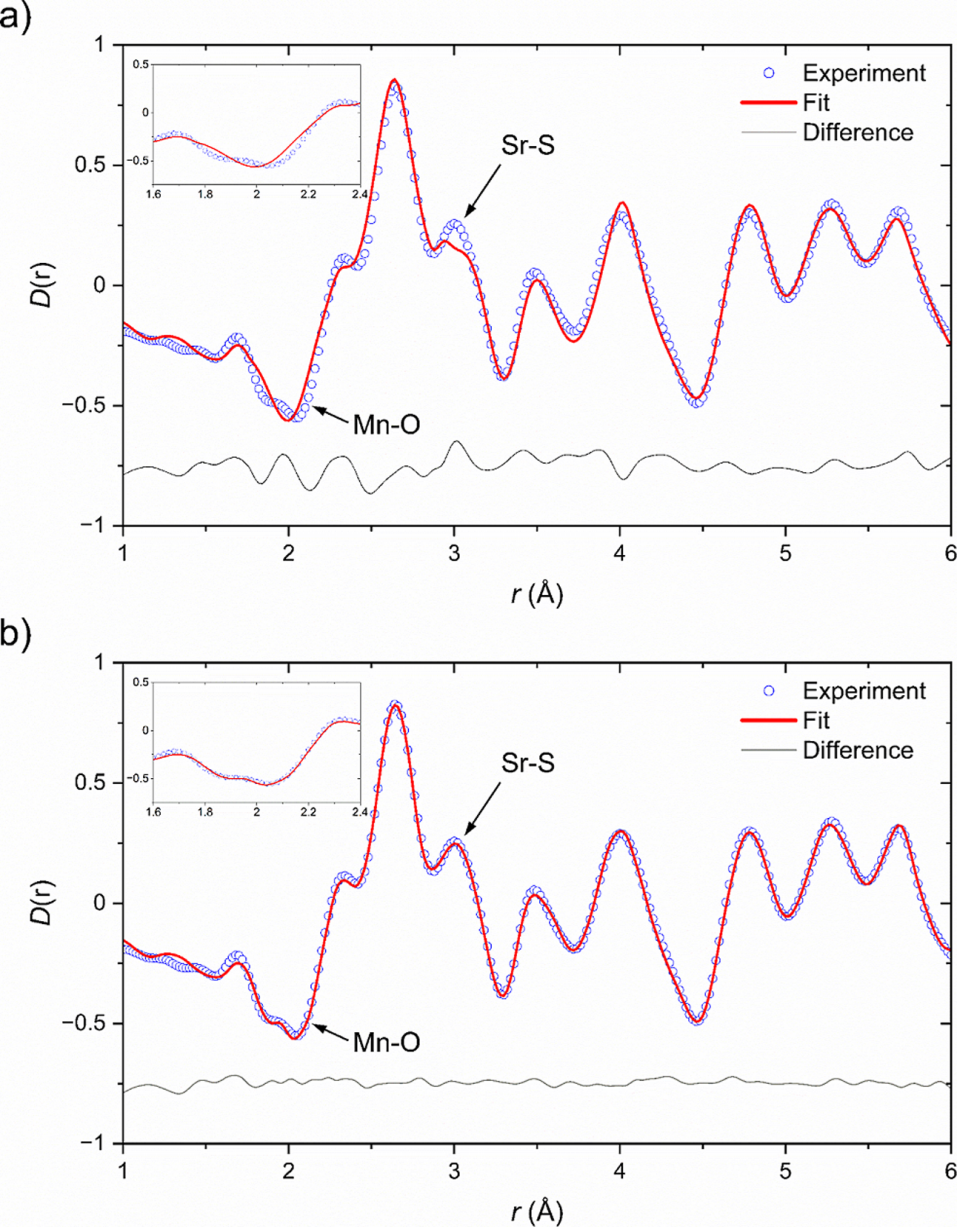
Neutron PDF fits for data collected at 250 K against the (a) room temperature disordered structural model in space group *P*4/*mmm* and (b) short-to-long-range fitting incorporating the CO structural model in the basic √2*a* × √2*a* × 2*c* expansion of the ambient temperature cell in space group *P*4_2_/*nmc* at low *r*.

### Magnetic Susceptibility Measurements

Figure 16 shows the magnetic susceptibility behavior for Sr_2_MnO_2_Cu_3.5_S_3_. The sharp cusp at 27 K is consistent with long-range antiferromagnetic ordering as reported previously and is identified as the Néel temperature (*T*_N_).^13^ Fitting of the inverse susceptibilities using the Curie–Weiss law (χ_mol_ = *C*/*T*-θ) where *C* is the Curie constant and θ is the Weiss constant, at temperatures between 200 and 300 K yielded an effective moment of 5.50(3) μ_B_, consistent with the spin-only expected value for a mean Mn-oxidation state of +2.5. The Weiss temperature obtained by this fitting is positive (θ = 21.2(1) K), suggesting that the dominant interactions between moments are ferromagnetic (Figure S22). A kink in the susceptibility close to *T*_CO_ (190 K) is observed and appears to be coupled to the structural and conductivity changes discussed. This observation can be rationalized by considering that below *T*_CO_, the only superexchange interactions between Mn^2/3+^ sites are ferromagnetic. Above *T*_CO_, antiferromagnetic Mn–O–Mn superexchange interactions between isoelectronic Mn sites are also present. A metamagnetic transition appears at approximately 1.1 T in magnetization isotherms measured at 20 K and below (i.e., below the transition to 3D long-range magnetic ordering), indicating ferromagnetic ordering. This feature persists to the lowest measured temperatures and at 1.8 K and 7 T, the compound is ferromagnetic with an apparent saturated moment of 3.7 μ_B_ per manganese ion from the magnetometry measurements.

**Figure 16:**
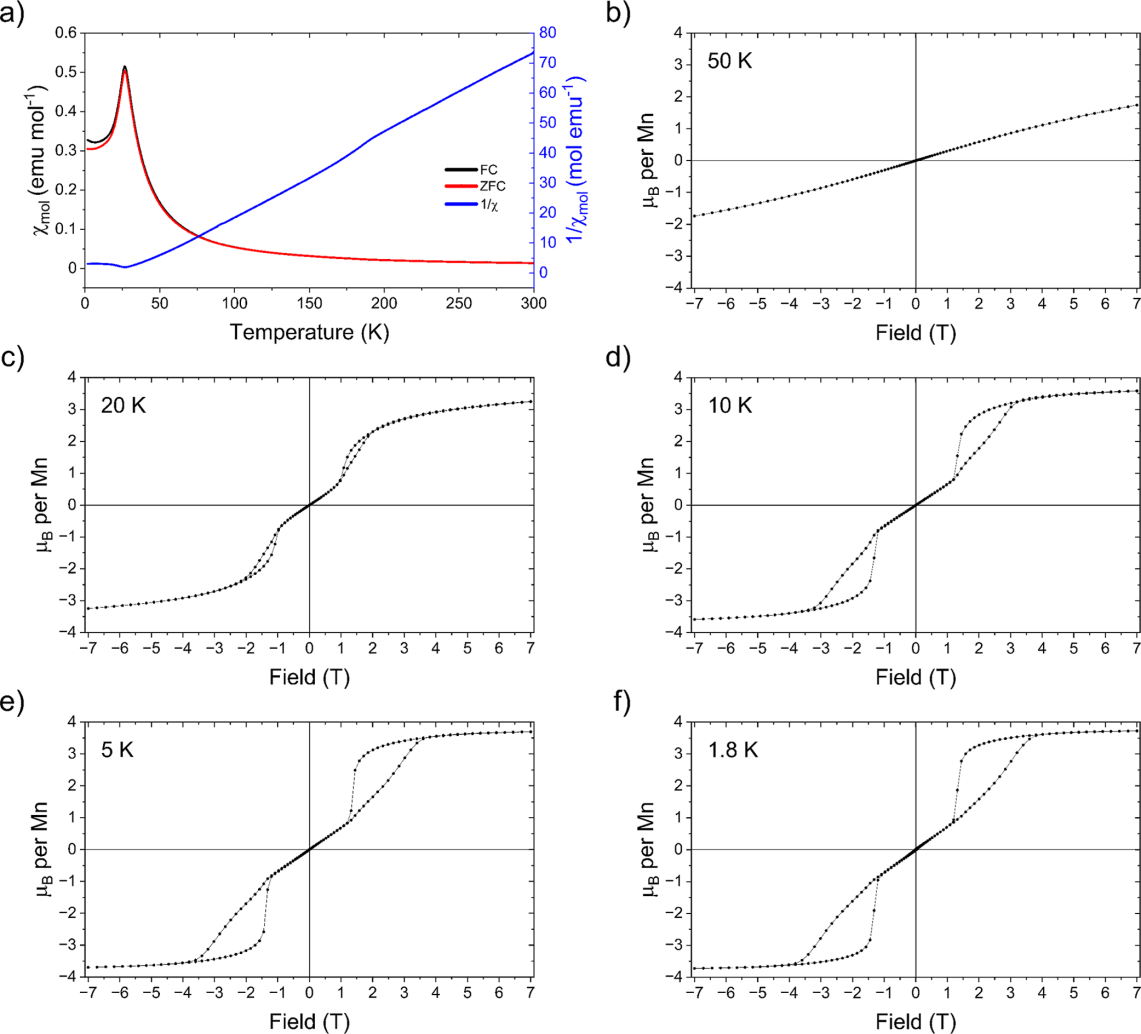
(a) Zero-field cooled and field-cooled temperature dependence of the magnetic susceptibility of Sr_2_MnO_2_Cu_3.5_S_3_ measured in a 1000 Oe field (fit shown in Figure S22). (b–f) Magnetisation isotherms measured on cooling from 298 K in a 5 T field to the measurement temperature and sweeping the field to −5 T and back to +5 T.

### Long Range Magnetic Ordering

Variable temperature NPD measurements of sample 2 were made on the GEM instrument and revealed the appearance of intense magnetic Bragg peaks at temperatures below 30 K, concentrated in the high d-spacing region, consistent with long-range magnetic ordering (Figure 17). It was possible to index the magnetic Bragg peaks with the propagation vector of **k** = (0 0 0) with respect to the superstructure (i.e., the √2*a* × √2*a* × 2*c* expansion of the ambient temperature cell in space group *P*4_2_/*nmc*), placing the k vector at the gamma point, and ISODISTORT^50,51^ was used to explore the possible symmetry adapted magnetic order modes consistent with the nuclear unit cell symmetry and the observed magnetic propagation vector.

**Figure 17:**
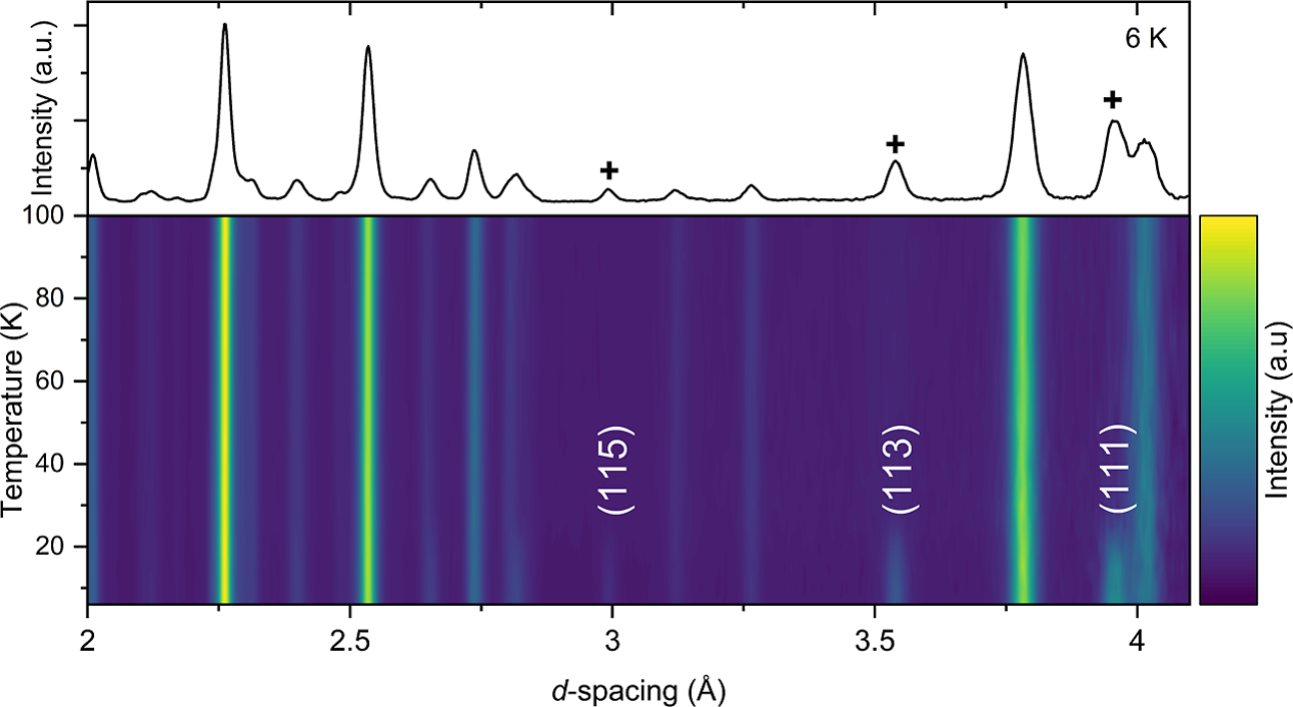
Plot of the NPD data collected for Sr_2_MnO_2_Cu_3.5_S_3_ (sample 2) obtained from 6 to 100 K on the GEM instrument. The black crosses (+) in the upper plot indicate magnetic Bragg reflections in the 6 K pattern which appear below 30 K. The respective *hkl* labels are denoted in the lower plot.

The activation of the mΓ_2_^-^ mode, corresponding to the magnetic space group *P*4_2_′/*n*′*m*′*c* (137.512) (BNS notation),^52,53^ was found to account for the additional intensity. The magnetic model is shown in Figure 18 and can be described as an *A*-type antiferromagnetic structure, with ferromagnetic moments of different magnitudes aligned parallel to the *c*-axis within the MnO_2_ planes which exhibit antiferromagnetic order between adjacent MnO_2_ layers. This model provided a fit with high statistical and visual quality and refinement of independent moments on the two Mn sites at 6 K produced values of 4.83(4) and 3.11(4) μ_B_ for Mn(1) and Mn(2) respectively. The refined moments are reduced from the spin-only values 5 and 4 μ_B_ expected for high-spin Mn^2+^ (d^5^) and Mn^3+^ (d^4^) ions, which is more pronounced for the Mn(2) site, presumably reflecting greater covalency of the Mn^3+^ ions. The refined parameters are given in Table 6. The thermal evolution of the magnetic moments on each of the Mn sites refined using this model is plotted in Figure 19. The refined moments are consistent with the CO state suggested by the bond valence sums derived from the nuclear structure and suggest significantly different σ-antibonding electron counts at the two Mn sites. The ferromagnetic ordering between Mn sites within the MnO_2_ planes is the likely origin of the positive Weiss temperature revealed by the magnetometry and can be understood by considering the dominant superexchange interactions between Mn centers with localized moments in a Mn^2+/3+^ charge-ordered checkerboard pattern due to the differing occupancies of the *d*_*x* ^2^–*y* ^2^_ orbitals.

**Figure 18:**
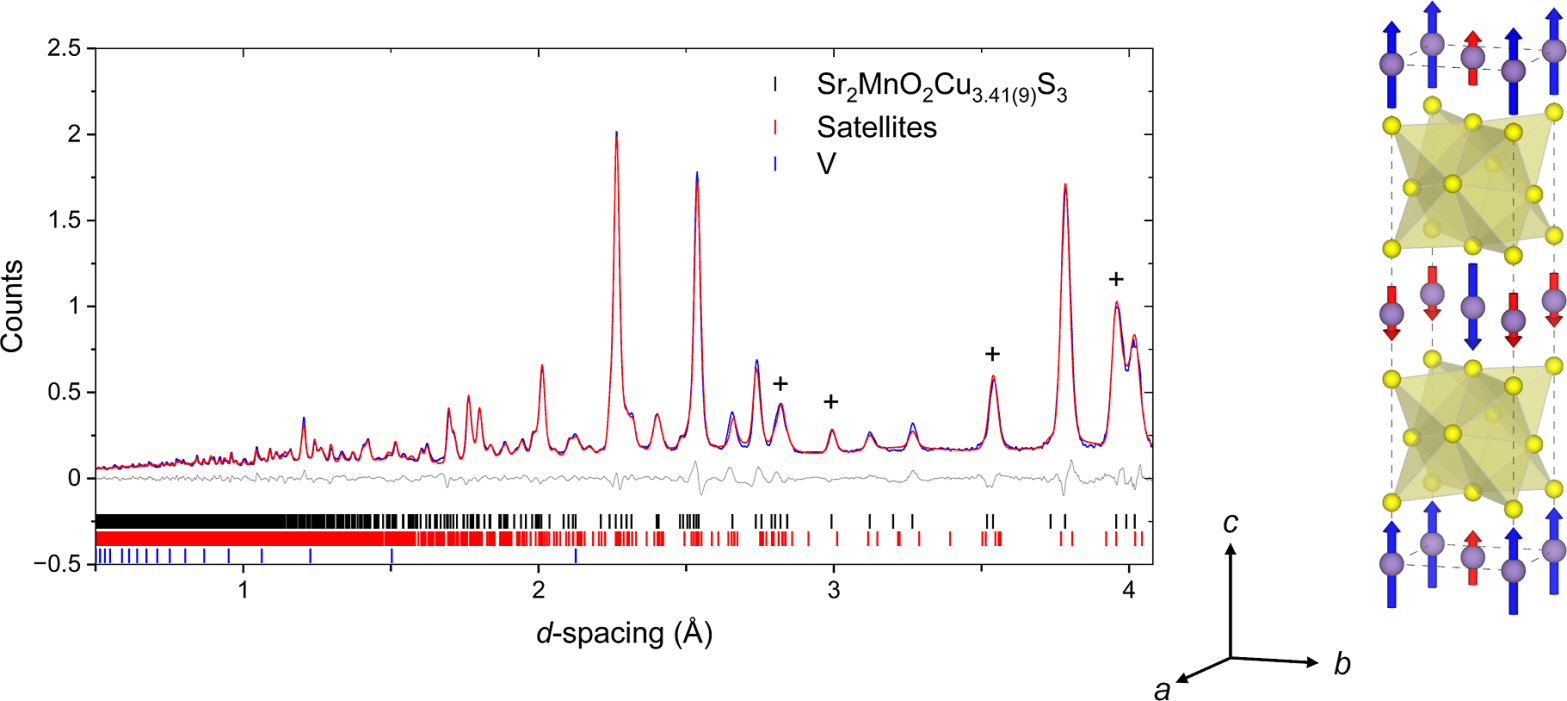
(left) Rietveld refinement of Sr_2_MnO_2_Cu_3.5_S_3_ (sample 2) against NPD data measured using bank 4 of GEM at 6 K. The most prominent magnetic peaks due to the ordering of Mn moments are labeled with a cross (+); (right) Refined magnetic structure at 6 K; for clarity, only the Mn atoms (lilac), and S atoms (yellow) are shown. The moment on the Mn(1) sites are indicated by blue arrows and those on the Mn(2) sites by red arrows. *R*_wp_ = 5.55%. The incommensurately modulated model for the structure is used in this refinement. We note that negligible magnetic intensity is found at the positions of the satellite reflections which, it is concluded, result only from the modulation of the Cu ions discussed above.

**Table 6:** Refined Parameters for the Magnetic Ordering in Sr_2_MnO_2_Cu_3.5_S_3_ (Sample 2) at 6 K

radiation	neutron, TOF
diffractometer	GEM
temperature (K)	6
magnetic space group	*P*4_2_′/*n*′*m*′*c* (137.512)
ordered Mn(1) moment (μ_B_)	4.83(4)
ordered Mn(2) moment (μ_B_)	3.11(4)
*R*_wp_ (%)	6.877

**Figure 19:**
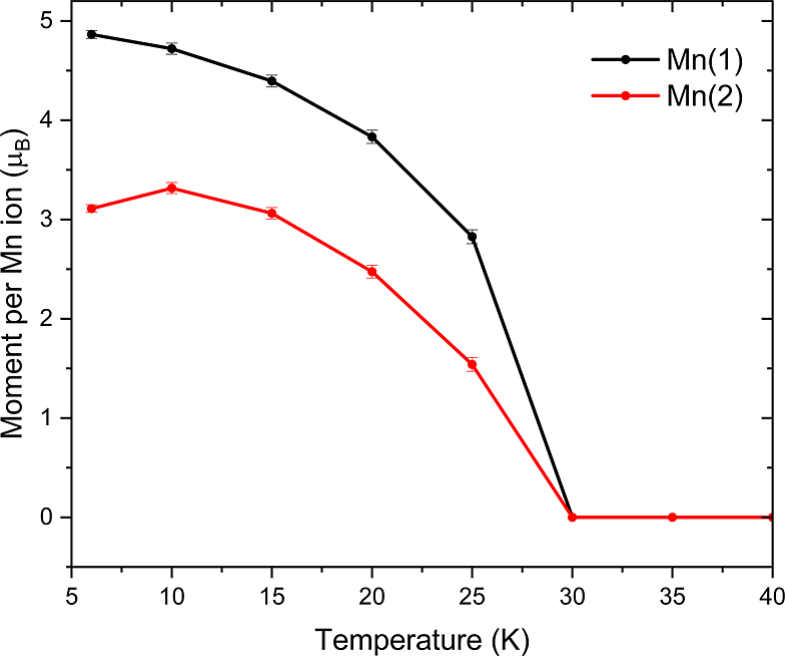
Ordered magnetic moments of the Mn(1) and Mn(2) sites versus temperature from Rietveld refinement of the A-type antiferromagnetic model described in the text, against NPD data collected on the GEM instrument.

To investigate the field dependence of the magnetic ordering, refinements were carried out against NPD measurements collected at 6 K in applied magnetic fields sweeping from 0 to 5 T and back again. Evident from the plot in Figure 20a, the intensities of the magnetic Bragg peaks associated with antiferromagnetic ordering decrease in intensity and eventually disappear in measuring fields exceeding 2.5 T. The decrease in intensity of these peaks is concomitant with additional intensity on top of the nuclear Bragg reflections suggesting that the compound enters the ferromagnetic regime (Figure 20b and d).

**Figure 20:**
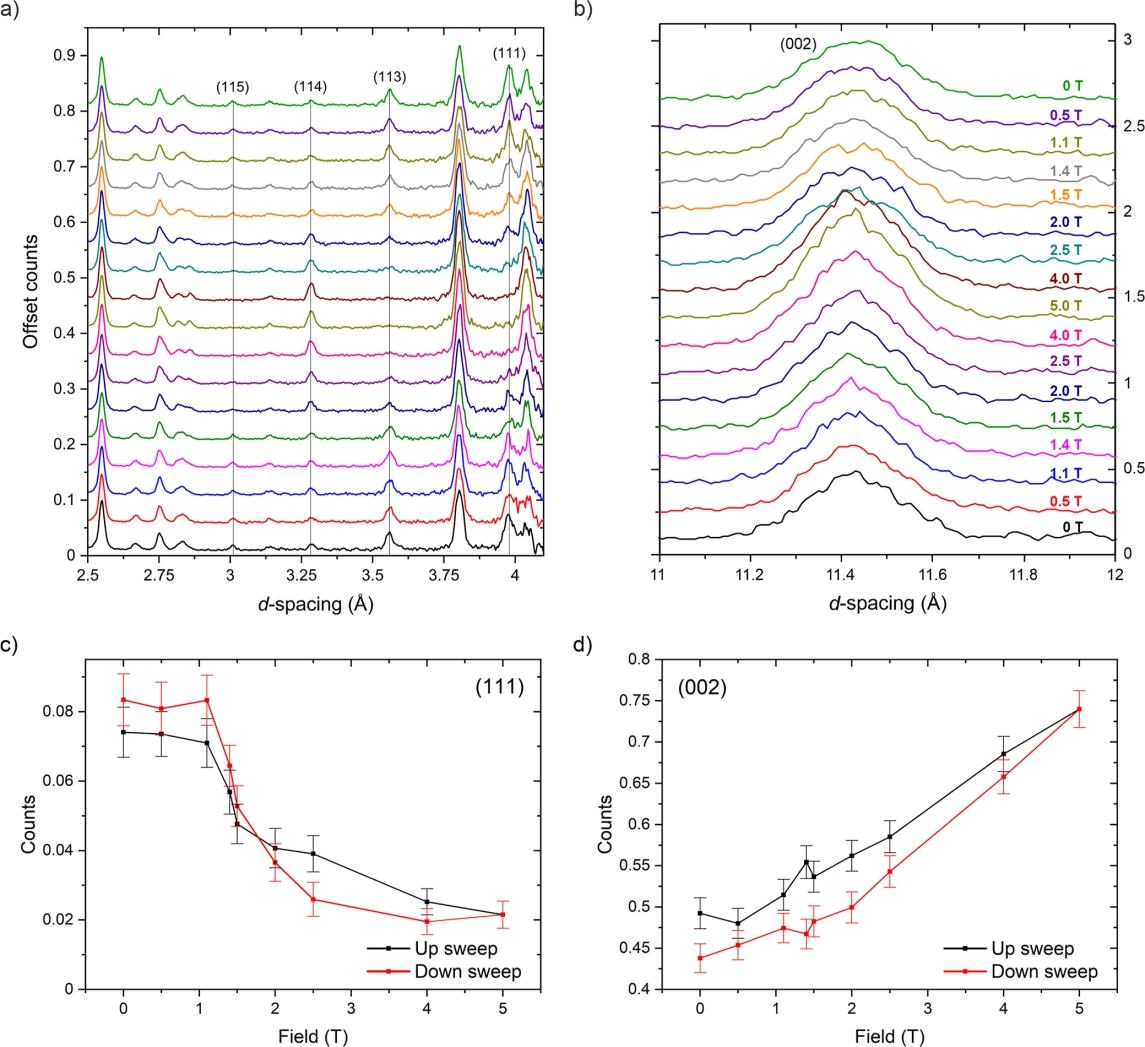
(a) Evolution of the neutron powder diffraction patterns of Sr_2_MnO_2_Cu_3.5_S_3_ in the range 2.5 ≤ *d* ≤ 4.1 Å and (b) 11 ≤ *d* ≤ 12 Å as a function of field. Data from bank 4 and 2 of the GEM instrument are shown collected from bottom to top. The intensity of the most prominent antiferromagnetic (111) peak (c) and the most intense nuclear reflection (002) (d) exhibit hysteresis on varying the field strength. The additional magnetic intensity at the position of the (002) nuclear reflection is greatest on the “up sweep”.

Here we note that for a powder sample for a tetragonal system it is only generally possible to define the orientation of the moment relative to the tetragonal axis.^54^ When a magnetic field is applied to a powder sample the moment direction relative to the crystallographic axes is even less well-defined, and it is, in general, challenging to define a unique magnetic model. Understanding the hysteresis observed in the magnetometry and the intensity variation with field of the magnetic Bragg peaks will likely require neutron diffraction experiments on single crystals aligned with the applied magnetic field.

The Rietveld refinement against NPD data collected at 5 K and 5 T is shown in Figure 21 using a charge-ordered, fully ferromagnetic model. The refined moments for the Mn(1) and Mn(2) sites are 4.2(2) and 3.6(2) μ_B_, respectively.

**Figure 21:**
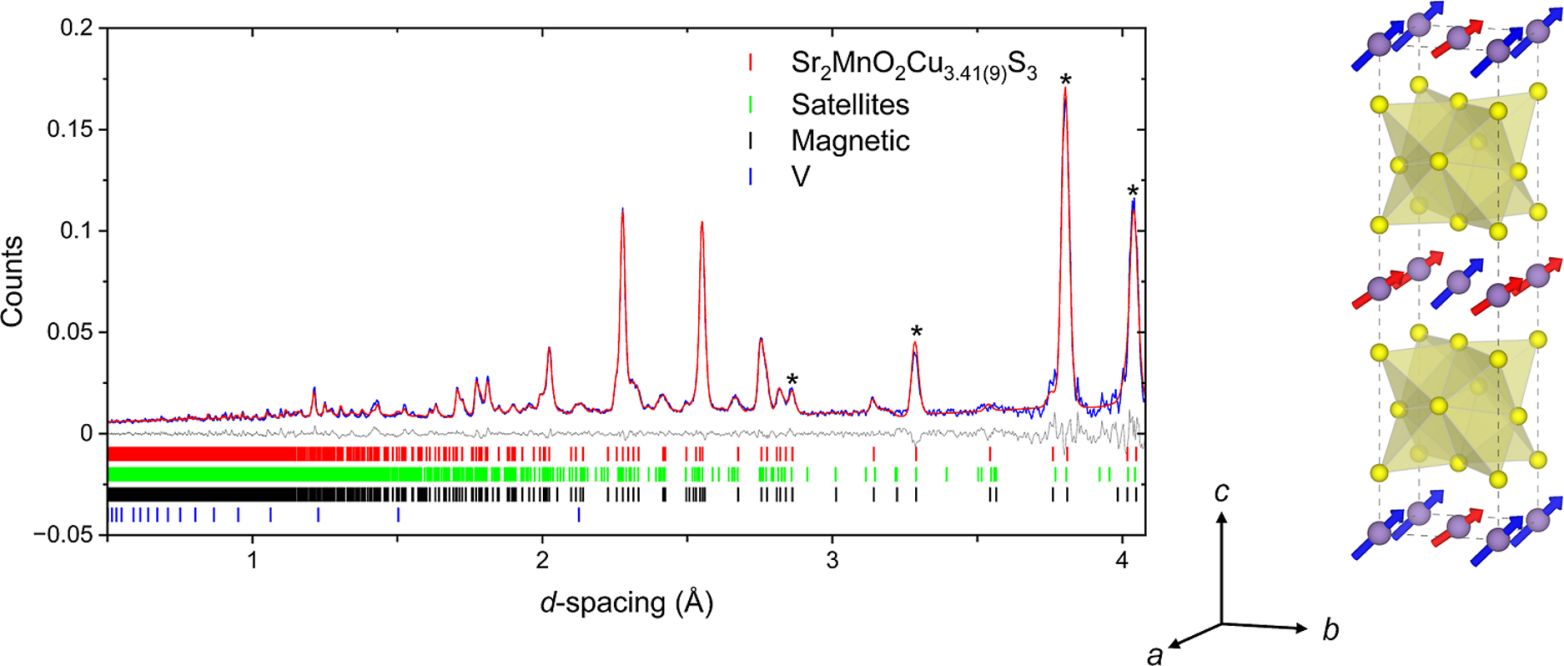
(left) Rietveld refinement of Sr_2_MnO_2_Cu_3.41(9)_S_3_ (sample 2) against NPD data measured using bank 4 of GEM at 5 K and 5 T. The most prominent magnetic peaks due to the ordering of Mn moments are labeled with an asterisk (*); (right) Refined magnetic structure at 5 K and 5 T; for clarity, only the Mn atoms (lilac), and S atoms (yellow) are shown. The moment on the Mn(1) sites are indicated by blue arrows and those on the Mn(2) sites by red arrows. *R*_wp_ = 6.94%. The incommensurately modulated model for the structure is used in this refinement. We note that negligible magnetic intensity is found at the positions of the satellite reflections which, it is concluded, result only from the modulation of the Cu ions discussed above.

At measuring fields below 1.4 T, all magnetic Bragg peaks could be accounted for using the A-type antiferromagnetic model observed at 0 T described solely by the magnetic mode mΓ_2_^-^. By 1.4 T, there is evidently a field-induced reorientation of the moments by the applied field and refined models require the incorporation of a ferromagnetic component to the magnetic structure and the eventual loss of the antiferromagnetic alignment of the moments in neighboring layers (Figures S19 and S20). In these refinements only the magnetic moments and the oxygen position parameters were allowed to freely refine. It was assumed that the other structural parameters would be largely unaffected by the field. Unconstrained refinements resulted in significant parameter correlation and worsened fits.

The total refined moment on each of the Mn sites decrease slightly with the increase in applied field until 1.1 T where a sharp increase in the moment localized at the Mn(2) site and a corresponding reduction of the moment on Mn(1) is observed. On further increase of the applied field, the magnetic moments at the Mn(1) and Mn(2) sites converge such that by 2.5 T the difference between the refined ordered moments is approximately 0.6 μ_B_, significantly reduced from the value of 1.72 μ_B_ at 0 T (Figure 22). The convergence suggests a decrease in the degree of charge separation between the two sites and a melting of the CO state on increasing the applied field. Similar behavior has been observed in Mn^3+/4+^ containing perovskite-type manganites.^55,56^ The field dependence of the magnetic structure of Sr_2_MnO_2_Cu_3.5_S_3_ mirrors the behavior of the charge ordered perovskite manganites, though field dependence of the resistivity at 5 K (ideally on large single crystals when available) would be required to fully characterize the electronic properties which are beyond the scope of the measurements described here.

**Figure 22:**
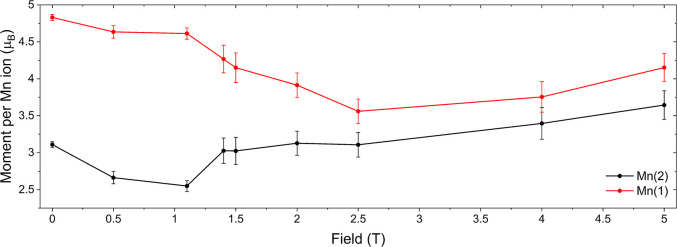
Magnitude of the long-range ordered magnetic moments on the two manganese sublattices in Sr_2_MnO_2_Cu_3.5_S_3_ as a function of field.

The NPD measurements are sensitive to the Mn–O distances and we show the evolution of the Mn–O distances over the range of fields measured which exhibit a similar convergence of the distinct Mn–O bond lengths, supporting a slight decrease in the degree of charge separation between the two Mn sublattices (Figure S21).

## Conclusions

The copper-deficient oxysulfide Sr_2_MnO_2_Cu_3.5_S_3_ presents a mixed-valent Mn^2+/3+^ compound to complement the well-known Mn^3+/4+^ oxides. A corollary of the mixed valency is the observation of a charge ordering transition below 190 K with checkerboard ordering of Mn^2+^ and Mn^3+^ ions which is similar to that adopted in some perovskite and Ruddlesden–Popper manganites with ratios of Mn^3+^:Mn^4+^ close to 1:1. Our detailed measurements using Bragg diffraction and PDF measurements reveal long-range charge order in Sr_2_MnO_2_Cu_3.5_S_3_ in contrast to the single-layer analogue, Sr_2_MnO_2_Cu_1.5_S_2,_ in which the low temperature average superstructure description does not feature long-range order of distinct Mn sublattices, although there was some evidence for charge separation in that case, as discussed in ref 8. A 9% difference between Mn–O bond distances for the nominally Mn^2+^ and Mn^3+^ sites is observed in this work on Sr_2_MnO_2_Cu_3.5_S_3_, which significantly exceeds that observed in the layered oxide manganites. This merits further investigation into the origin of the charge order in this compound and whether the frameworks developed for the oxides can be applied to Sr_2_MnO_2_Cu_3.5_S_3_ and other mixed-valent mixed-anion compounds. Incommensurate modulations of the copper site occupancy factors in the cation-deficient Cu sulfide layers are observed and this additional complexity correlates with our previous observations in related oxysulfides. Analysis of the local structure in this compound reveals behavior consistent with conventional order/disorder transitions and suggest the presence of locally charge-ordered regions at temperatures above the long-range structural transition. The A-type antiferromagnetic structure adopted with different magnetic moments on each of the Mn sublattices reflects the checkerboard ordering of Mn^2+^/Mn^3+^ cations within the ferromagnetic MnO_2_ planes. Application of magnetic fields in excess of 1.1 T triggers a metamagnetic transition with spin reorientation of the Mn moments resulting in a bulk ferromagnetic structure and a decrease in the extent of charge ordering as determined by Mn–O bond lengths.

## Supplementary Material



## Notes

Further tables of refinement parameters, figures showing SEM–EDX measurements, powder diffraction data, modulation parameters, additional de Wolff plots and discussion (PDF)
