# Supplementary material for: Charge Ordering and Incommensurate Modulations in the Metamagnetic Layered Manganese Oxysulfide Sr2MnO2Cu3.5S3
Source: J Am Chem Soc. 2026 Jan 16;148(3):3830–51. doi: 10.1021/jacs.5c21494 (PMC12856914; doi:10.1021/jacs.5c21494)
Supplement: Supplementary file 1 [file ja5c21494_si_001.pdf]

Charge ordering and incommensurate modulations in the metamagnetic layered manganese oxysulfide $Sr_2MnO_2Cu_{3.5}S_3$

Lemuel E. Crentsil[1,*], Oliver J. Rutt[1], David G. Free[1], Murray J. David[1], Robert D. Smyth[1], Catherine F. Smura[1], David A. Keen[2], Andrew N. Fitch[3], Joke Hadermann[4], and Simon J. Clarke[1,*]

Supporting information

[1] *Department of Chemistry, University of Oxford, Inorganic Chemistry Laboratory, South Parks Road, Oxford, OX1 3QR, UK*

[2] *ISIS Facility, Rutherford Appleton Laboratory, Harwell Campus, Didcot, Oxfordshire, OX11 0QX, UK*

[3] *European Synchrotron Radiation Facility - 71, avenue des Martyrs, CS 40220, 38043 Grenoble Cedex 9, France*

[4] *Electron Microscopy for Materials Science (EMAT), University of Antwerp, Groenenborgerlaan 171, B-2020 Antwerp, Belgium.*

*Corresponding authors

email: simon.clarke@chem.ox.ac.uk

Supplementary Figures

Figure S1. Experimental values of the *M*-S/*M*-O bond distance ratios of some Sr-containing oxysulfides
Figure S2. Semi-quantitative SEM-EDX images and analysis of $Sr_2MnO_2Cu_{3.5}S_3$ (sample 1).
Figure S3. Rietveld refinement of $Sr_2MnO_2Cu_{3.5}S_3$ (sample 1) against NPD data collected at ambient temperature from banks 1, 2, 3, 5 & 6 of the GEM instrument
Figure S4. Rietveld refinement of $Sr_2MnO_2Cu_{3.5}S_3$ (sample 2) against NPD data collected at ambient temperature from banks 2, 3, 4, 5 & 6 of the GEM instrument
Figure S5. Evolution of superstructure reflections of $Sr_2MnO_2Cu_{3.5}S_3$ (sample 1) with temperature measured on beamline ID22.
Figure S6. Reconstructed precession image in the 0*kl* layer from single crystal X-ray diffraction frames collected at 100 K.
Figure S7. Reconstructed precession image in the *hk*1 layer from single crystal X-ray diffraction frames collected at 100 K.
Figure S8. Evolution of Mn-O bond distances and copper occupancies with temperature in $Sr_2MnO_2Cu_{3.5}S_3$ (sample 2) from refinement against GEM data
Figure S9. Temperature dependence of resistivity for $Sr_2MnO_2Cu_{3.5}S_3$ on successive heating and cooling cycles
Figure S10. x_2-x_4 de Wolff section showing the observed electron density in superspace, centred on the S(1) and the Sr(1) atoms
Figure S11. Variation of the displacement of the sulfur atoms within the *ab* plane in the superstructure of $Sr_2MnO_2Cu_{3.5}S_3$ as a function of t
Figure S12. Variation of the Sr-S distances in the superstructure of $Sr_2MnO_2Cu_{3.5}S_3$ as a function of t and u
Figure S13. Variation of the site occupancy factors of the copper atoms in the copper sulfide slab centred at $z = 0.25$ in the superstructure $Sr_2MnO_2Cu_{3.5}S_3$ as a function of t and u.
Figure S14. Fourier map of the observed electron density in the vicinity of the copper sites as function of x_4
Figure S15. Variation of the site occupancy factors of the Cu(2) atom and its equivalent atom generated by the 4_2 screw axis
Figure S16. Plot of the site occupancy factor of Cu(1) against volume of the CuS_4 tetrahedra.
Figure S17. Rietveld refinement of $Sr_2MnO_2Cu_{3.5}S_3$ (sample 1) using the average structural model of the superstructure in space group $P4_2/nmc$
Figure S18. Rietveld refinement of $Sr_2MnO_2Cu_{3.5}S_3$ (sample 3) against data collected on beamline ID22 at 10 K using the superspace model including the modulation
Figure S19. Rietveld refinement of the crystal and magnetic structures of $Sr_2MnO_2Cu_{3.5}S_3$ (sample 2) against NPD data collected using bank 4 of the GEM instrument on increasing the field from 0 to 5 T
Figure S20. Evolution of the magnetic unit cell of $Sr_2MnO_2Cu_{3.5}S_3$ in the magnetic field range 0-5 T
Figure S21. Variation of Mn-O bond distances in $Sr_2MnO_2Cu_{3.5}S_3$ with applied field from 0 to 5 T
Figure S22. Curie-Weiss fitting to the inverse magnetic susceptibility of $Sr_2MnO_2Cu_{3.5}S_3$ and plot of $\chi_{mol}T$ against temperature

Supplementary Tables

Table S1. Comparison of lattice parameters and selected bond lengths of some Sr-containing oxysulfides

Table S2. Selected bond lengths for $Sr_2MnO_2Cu_{3.5}S_3$ (sample 1) at 293 K (GEM and ID22 data)

Table S3. Single crystal refinement details of the average structure at 100 K in space group $P4_2/nmc$

Table S4. Single crystal refinement details of the average structure at 100 K in space group *Pmmn*

Table S5. Bond valence sums of $Sr_2MnO_2Cu_{3.5}S_3$ (sample 2) derived from structural refinements against NPD data collected on the GEM instrument

Table S6. Modulation parameters of the atomic positions of $Sr_2MnO_2Cu_{3.5}S_3$ (sample 1) derived from single crystal XRD

Table S7. Modulation parameters of the anisotropic displacement parameters of $Sr_2MnO_2Cu_{3.5}S_3$ (sample 1) derived from single crystal XRD.

Table S8. Modulation parameters of the site occupancy factors for the Cu atoms in $Sr_2MnO_2Cu_{3.5}S_3$.

Comparison with related layered oxysulfides

As the e_g orbital degeneracy in an octahedral high-spin Mn^{3+} (d^4) system drives Jahn-Teller distortions in molecules and compounds, it is worth making comparison with the crystal structures of other Sr-containing members of this structural series (Table S1 and Figure S1). The ratios of the *M*-S/*M*-O bond distances are considerably smaller in both Mn-containing compounds than in the series of 'single-layer' compounds $Sr_2MO_2Cu_2S_2$ with *M* = Mn–Zn[1-5]. The *a* lattice parameter is twice the *M*-O bond distance and decreases across the series, in line with the contraction of the ionic radii and appears to be coupled to elongation of the axial *M*-S distance. Clearly, the electron count and resulting occupancies of the t_{2g} and e_g orbitals of the *M* ion in the $Sr_2MO_2Cu_2S_2$ series influence the anisotropic ligand field of the MO_4S_2. Additional electronic effects are also apparent, particularly in $Sr_2CuO_2Cu_2S_2$. The e_g orbital degeneracy in an octahedral d^9 Cu^{2+} system drives a strong Jahn–Teller distortion and thus the ratio of axial/equatorial distances is greater than in the Ni-analogue[4]. The increase of the axial/equatorial distances for the Mn(2) site is entirely consistent with a strong Jahn-Teller distortion.

Table S1. Comparison of lattice parameters and selected bond lengths of some oxysulfides**.**

		$Sr_2MO_2Cu_2S_2$				
Compound	$Sr_2MnO_2Cu_{3.5}S_3$	Mn[a]	Co	Ni	Cu	Zn
reference	This work	1	2	3	4	5
radiation	NPD, SCXRD	NPD	NPD	NPD	NPD	NPD
a (Å)	4.016345(1)	4.01216(3)	3.99129(2)	3.92159(2)	3.92016(6)	4.0079(7)
r(*M*) (Å)	0.83	0.83	0.745	0.69	0.73	0.74
M-S	2.8769(6)	2.9300(9)	3.0327(5)	3.1054(9)	3.1085(15)	3.021(4)
M-O	2.008173(1)	2.00609(1)	1.99565(1)	1.96080(1)	1.96008(3)	2.0040(4)
M-S/*M*-O	1.4326(6)[b] 1.320(2)[c] 1.502(2)[d]	1.4605(4)	1.5197(3)	1.58374(5)	1.5859(8)	1.507(2)

[a]$Sr_2MnO_2Cu_2S_2$ contains a deficiency of 0.5 Cu(I) ions per Mn ion and is formulated as $Sr_2MnO_2Cu_{1.5}S_2$
[b]Room temperature; [c]Mn(1) at 100 K; [d]Mn(2) at 100 K

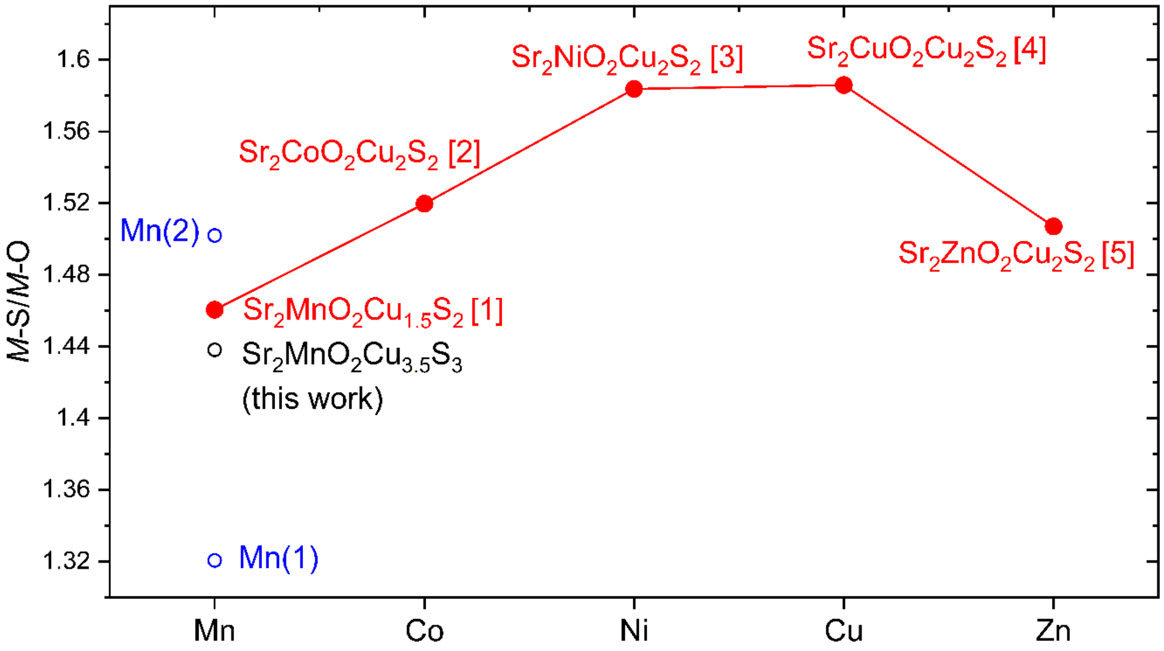

Figure S1. Experimental values of the *M*-S/*M*-O bond distance ratios for $Sr_2MnO_2Cu_{3.5}S_3$ at room temperature (black open circle), the distinct Mn sites observed at 100 K (blue open circles) and the series $Sr_2MO_2Cu_2S_2$ (*M* = Mn – Zn) (red closed circles).

The closest oxide structural analogue of $Sr_2MnO_2Cu_{3.5}S_3$ is the layered phase $La_{0.5}Sr_{1.5}MnO_4$ containing vertex-linked axially elongated MnO_6 octahedra. Several reports have indicated a charge ordered state appears below 220 K resulting in a large change in the resistivity and suppression of the magnetic susceptibility, the coincident orbital order has also been directly observed[6-8]. In $La_{0.5}Sr_{1.5}MnO_4$ in which the axial/equatorial ratio at room temperature is 1.04, below T_{CO} the appearance of *hhl* superstructure peaks is associated with a modest breathing mode distortion indicating a fractional change in the Mn-O bond lengths of 1%[8]. Studies of the lattice effects on the charge ordering transition in the series $Ln_{0.5}Sr_{1.5}MnO_4$ (*Ln* = La, Nd, Sm) show that T_{CO} is very sensitive to the static Jahn-Teller effect inherent to the layered structure[9]. T_{CO} is suppressed and eventually disappears as the axial/equatorial ratio is increased from 1.04 to 1.05 in the Nd compound as the in-plane Mn-O bonds contract with the introduction of smaller lanthanides cations. In comparison, the $Sr_2MnO_2Cu_{2m-0.5}S_{m+1}$ compounds exhibit much larger axial/equatorial ratios due to the substitution of S for O and yet our Rietveld analysis indicates a difference between the Mn-O bond distances ≃ 9 % in the CO state of $Sr_2MnO_2Cu_{3.5}S_3$. This appears to be a similarity between the two series and may explain why charge

order is observed in $Sr_2MnO_2Cu_{3.5}$ with an axial/equatorial ratio of 1.4326(9) but is not evident in the average structure of $Sr_2MnO_2Cu_{1.5}S_2$ where the axial/equatorial ratio increases to 1.4605(4)[1].

Supplementary Figures

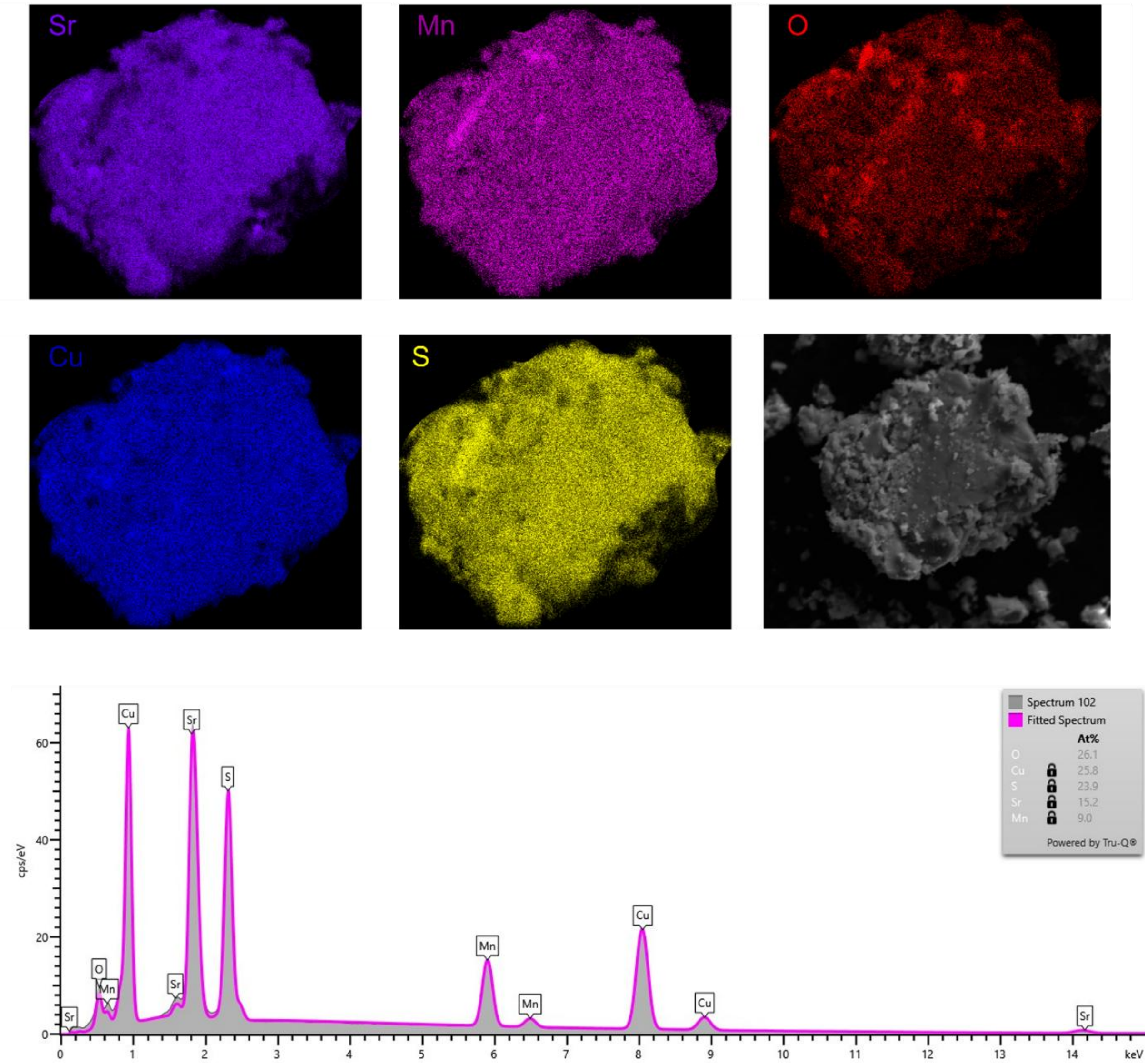

Figure S2 (Top) SEM-EDX imaging and (bottom) elemental mapping of a $Sr_2MnO_2Cu_{3.5}S_3$ crystallite.

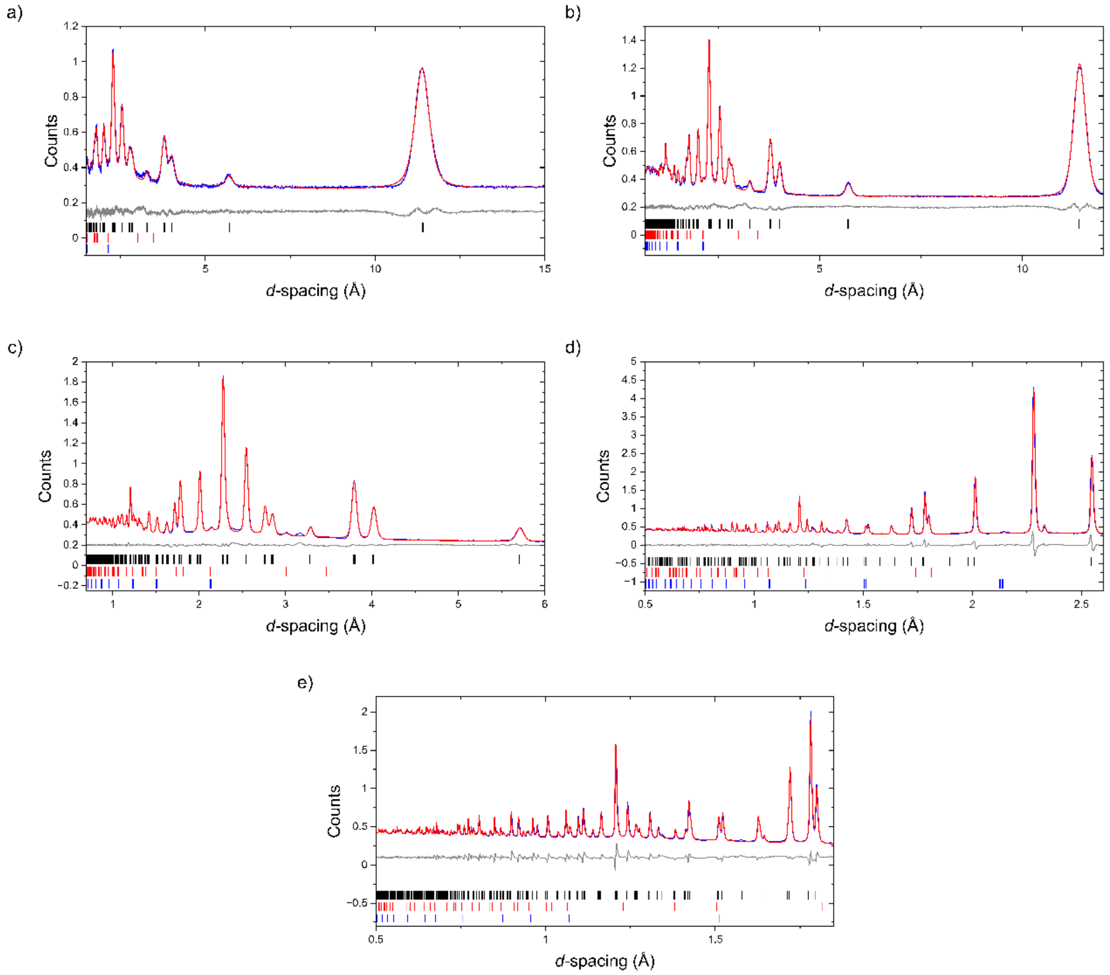

Figure S3. Rietveld refinement of $Sr_2MnO_2Cu_{3.5}S_3$ (sample 1) at ambient temperature from bank (a) 1 (9.39°), (b) 2 (17.98°), (c) 3 (34.96°), (d) 5 (91.37°) & (e) 6 (154.46°) of the GEM instrument. The refinement against data from bank 4 (63.62°) is shown in the main text.

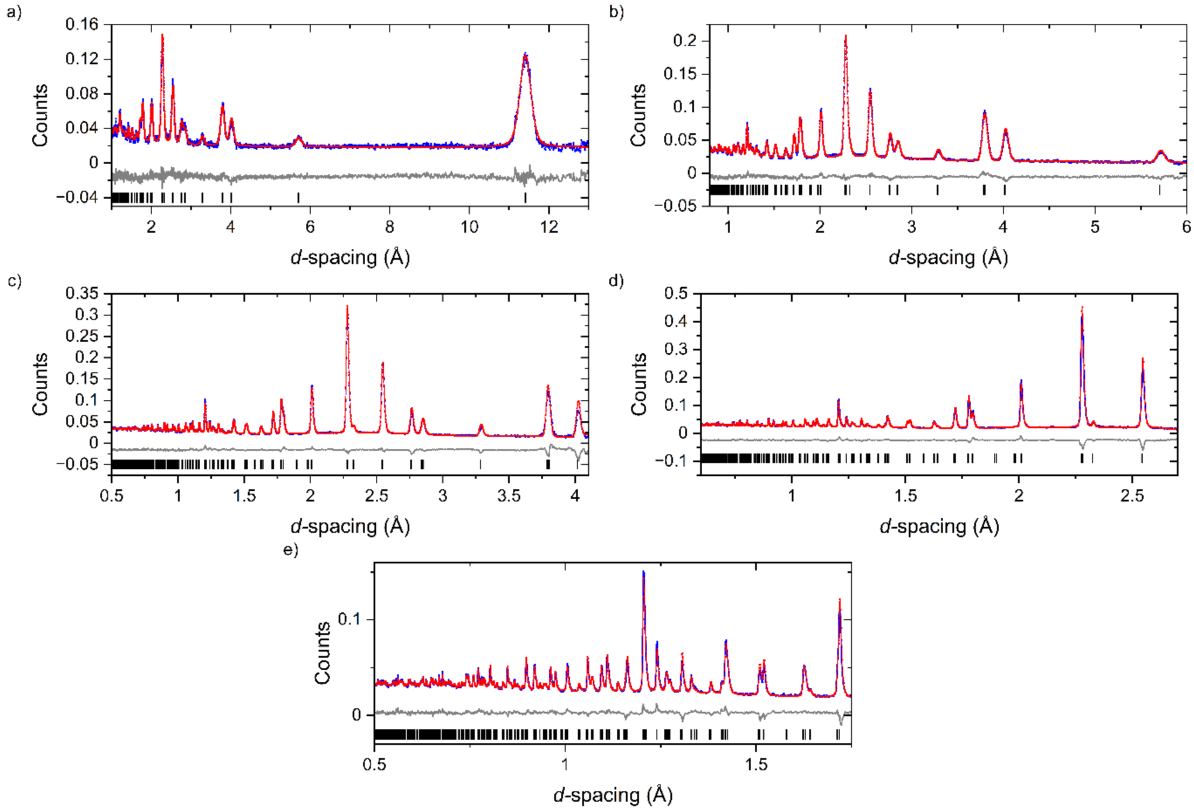

Figure S4. Rietveld refinement of $Sr_2MnO_2Cu_{3.5}S_3$ (sample 2) at ambient temperature from bank (a) 2 (17.98°), (b) 3 (34.96°), (c) 4 (63.62°), (d) 5 (91.37°) & (e) 6 (154.46°) of the GEM instrument.

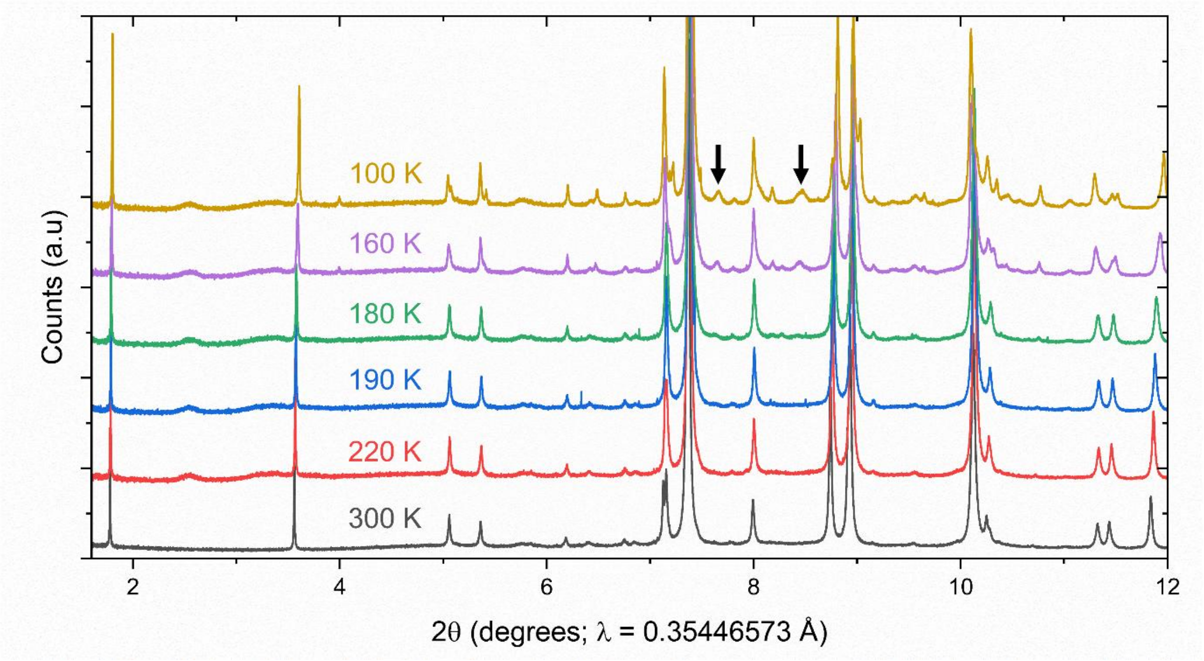

Figure S5. Evolution of superstructure reflections of $Sr_2MnO_2Cu_{3.5}S_3$ (sample 1) with temperature measured on ID22. The presence of the satellite peaks is indicated by the arrows

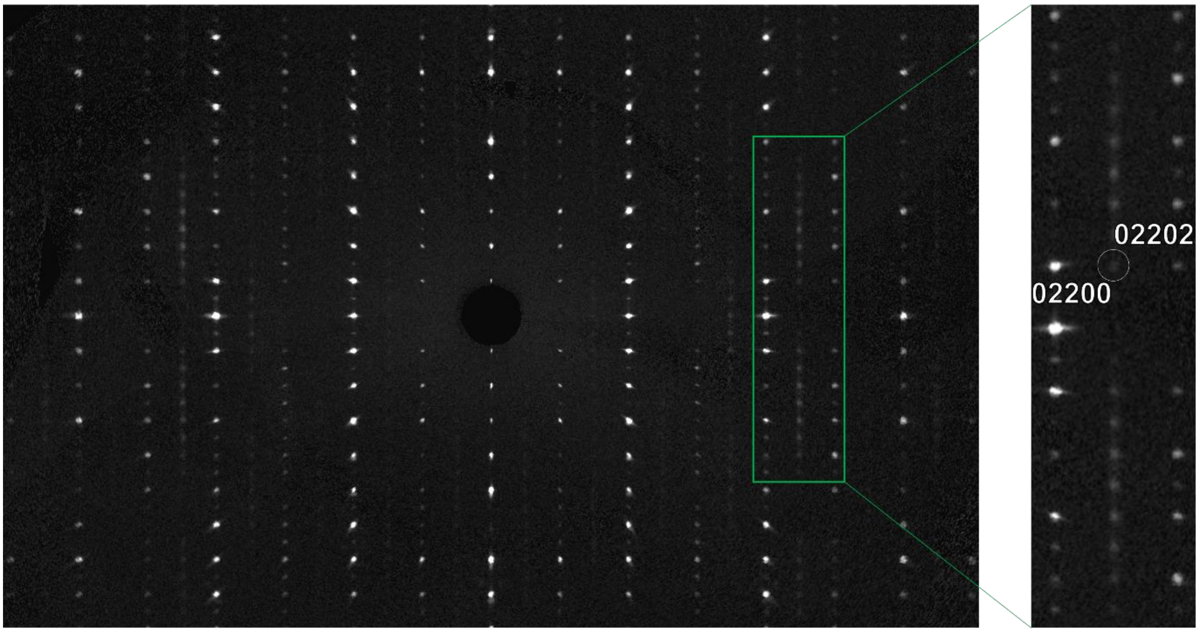

Figure S6. Reconstructed precession image in the *0kl* layer from single crystal X-ray diffraction frames collected at 100 K. Only second order satellite reflections are observed, consistent with the reflection condition 0*kl*0*n*: *n* = 2n.

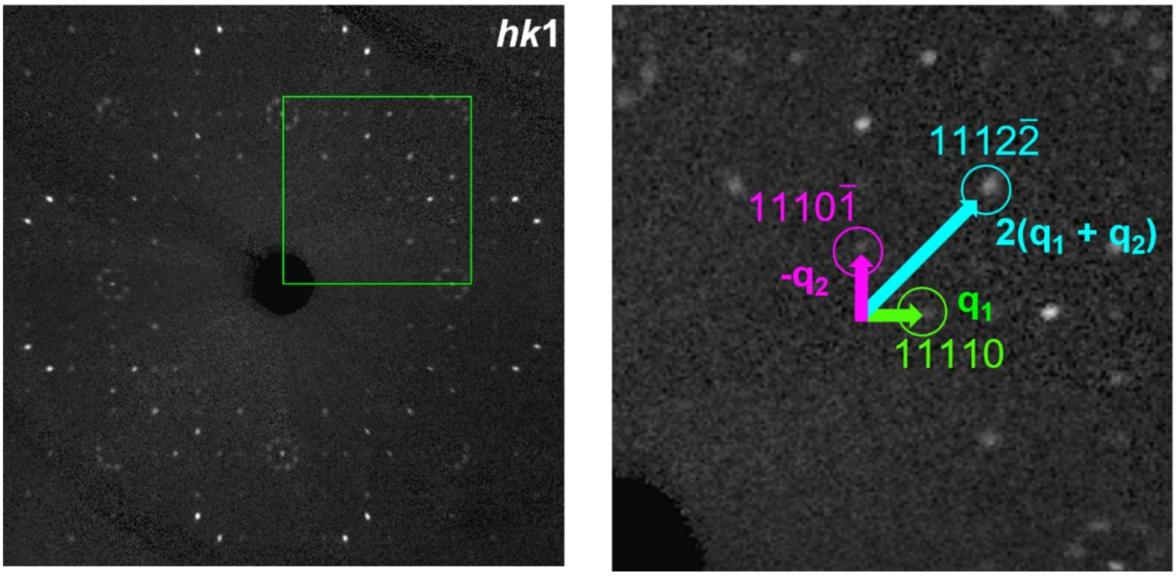

Figure S7. Reconstructed precession image in the *hk*1 layer from single crystal X-ray diffraction frames collected at 100 K. ‘Mixed’ satellite reflections (*h*, *k*, 1, ±2, ±2) are observed, consistent with the reflection condition *hhlmn*: *m*, *n* = 2*p*.

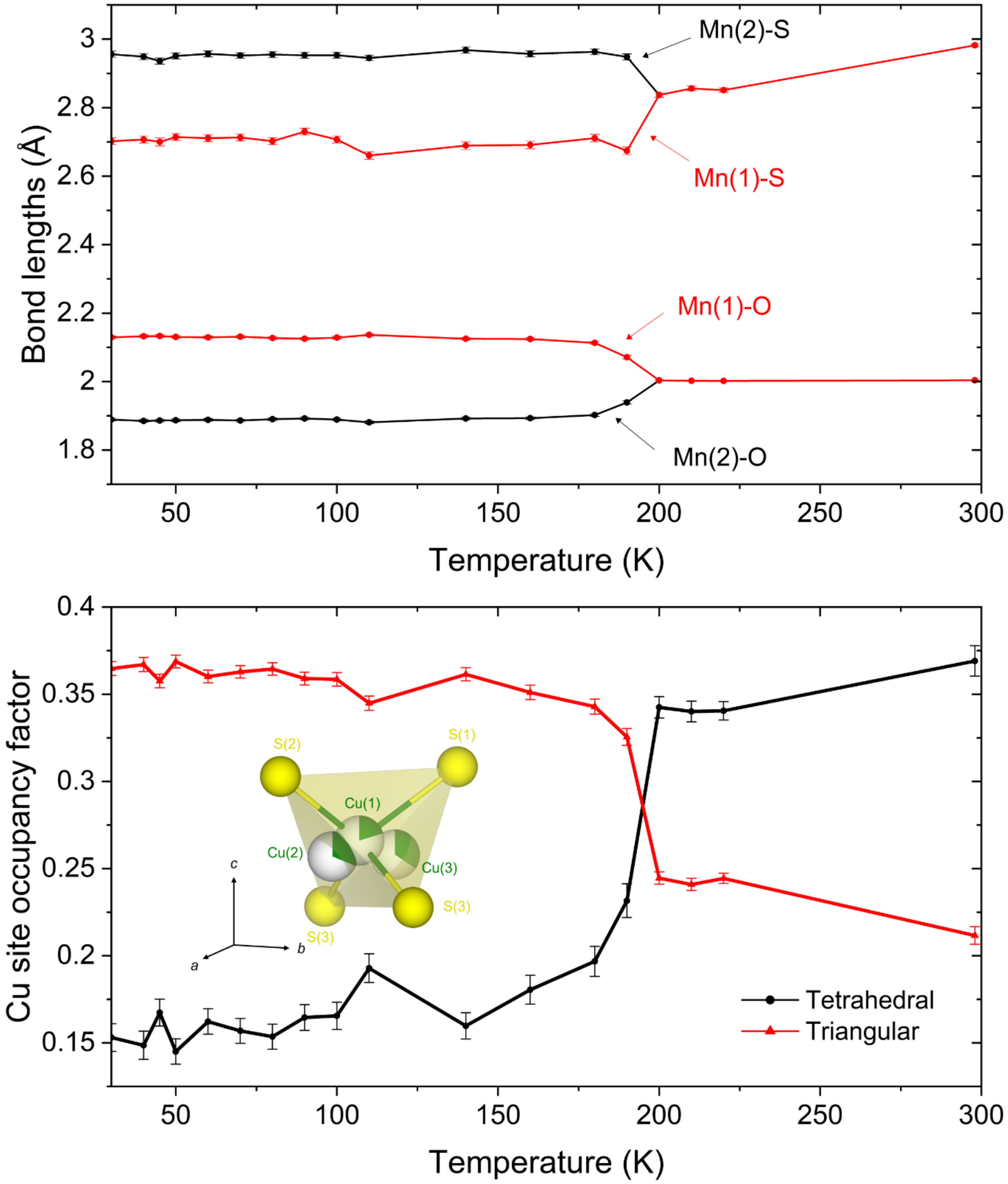

Figure S8. Thermal evolution of (a) Mn bond distances and (b) fractional occupancy of the tetrahedral and trigonal copper sites in $Sr_2MnO_2Cu_{3.5}S_3$ (sample 2) from refinement against GEM data using the CO model in space group $P4_2/nmc$ below 200 K and the disordered $P4/mmm$ model above 200 K.

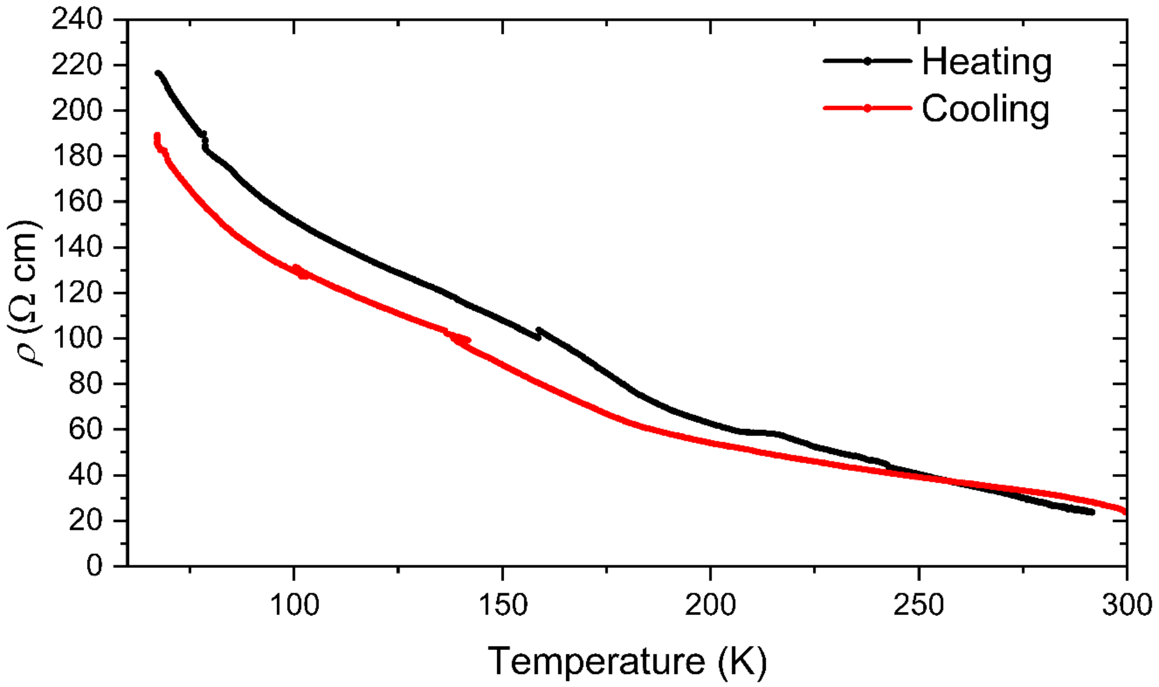

Figure S9. Temperature dependence of resistivity for $Sr_2MnO_2Cu_{3.5}S_3$ on successive heating and cooling cycles

Structural Modulations.

In incommensurately modulated structures, the deviations of the structural parameters of an atom (displacement, site occupancy factor, anisotropic displacement parameter etc.) from their values in the basic unit cell in 3D physical space are determined as a function of the periodic modulation function $\bar{u}(\bar{x}_4,\bar{x}_5)$. The generalised electron density in the vicinity of a particular atom can be plotted in a 2D section of superspace. In this formalism developed by de Wolff the variation of the electron density centred on a specific atom in real space is illustrated as a function of x_4 or x_5[10]. In these plots, the three-dimensional coordinates x, y, and z are termed x_1, x_2, and x_3 whilst x_4 and x_5 are the internal coordinates determining the magnitude and direction of the modulation function. Periodicity in superspace is ensured by the property $\bar{x}(x_4) = \bar{x}(x_4 + 1)$. Where $\bar{x}$ is the structural parameter, usually x_1, x_2 or x_3.

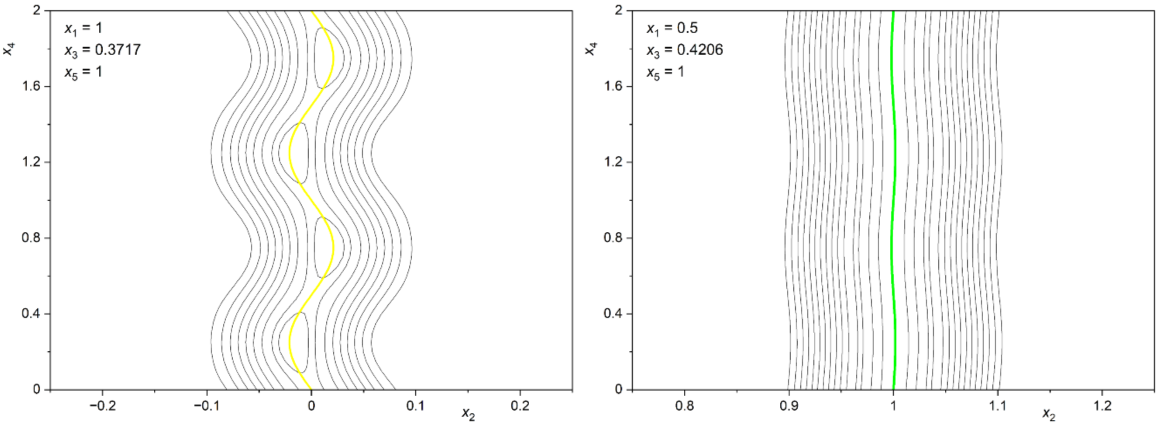

Figure S10. x_2-x_4 de Wolff section showing the observed electron density in superspace, centred on the S(1) (left) atom and the Sr(1) atom (right) . The superspace x_2 coordinate is equivalent to the *b* axis in three-dimensional space. The superspace x_4 coordinate and is defined by t + $\mathbf{q_1}\cdot\boldsymbol{x}$ where t is the phase of the modulation and **q₁ =** 0.2582(5)**a***. Therefore, these plots indicate transverse displacive modulations as superspace is traversed along the x_1(*a*) direction, i.e. the x_2(y) coordinates of S(1) and Sr(1) atoms are modulated. The refined position of each atom in the section is indicated by the coloured line

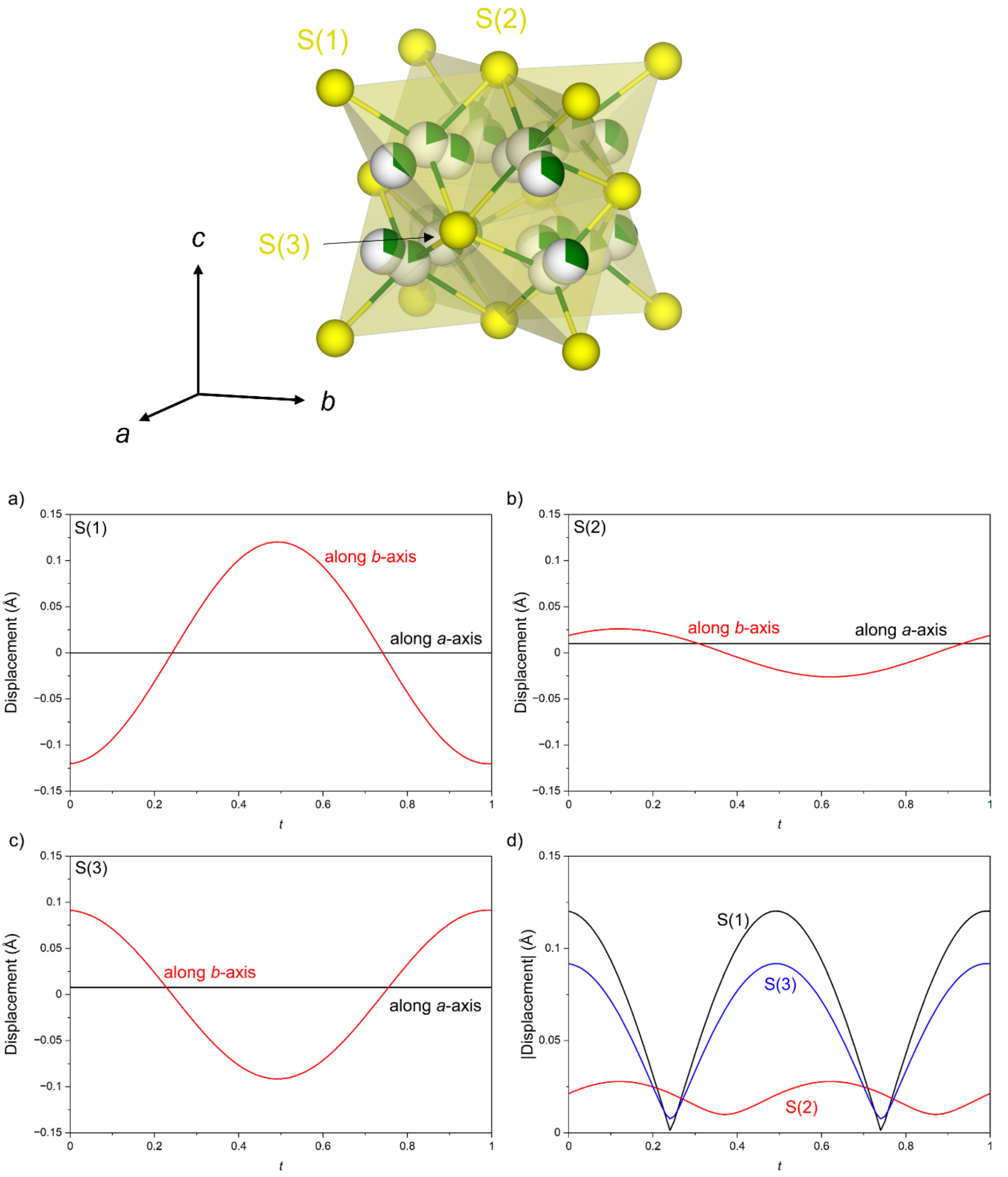

Figure S11. *t*-plots of the atom displacement in the *ab* plane for each of the atoms (a) S(1), (b) S(2) and (c) S(3). The modulus of the displacement of each of the sulfur atoms is shown in (d). The maximum values of the displacement of S(1) and S(3) occur at $t = 0.5$ with the S(1) atom displaced in the +*b* direction whilst the displacement of the S(3) is almost equal and opposite. This implies a minimum volume of the CuS_4 tetrahedra at $t = 0.5$ which is where the maximum occupancy of the Cu(1) site is observed in Figure 7 in the main text. We note that the displacement along the *c* direction is zero.

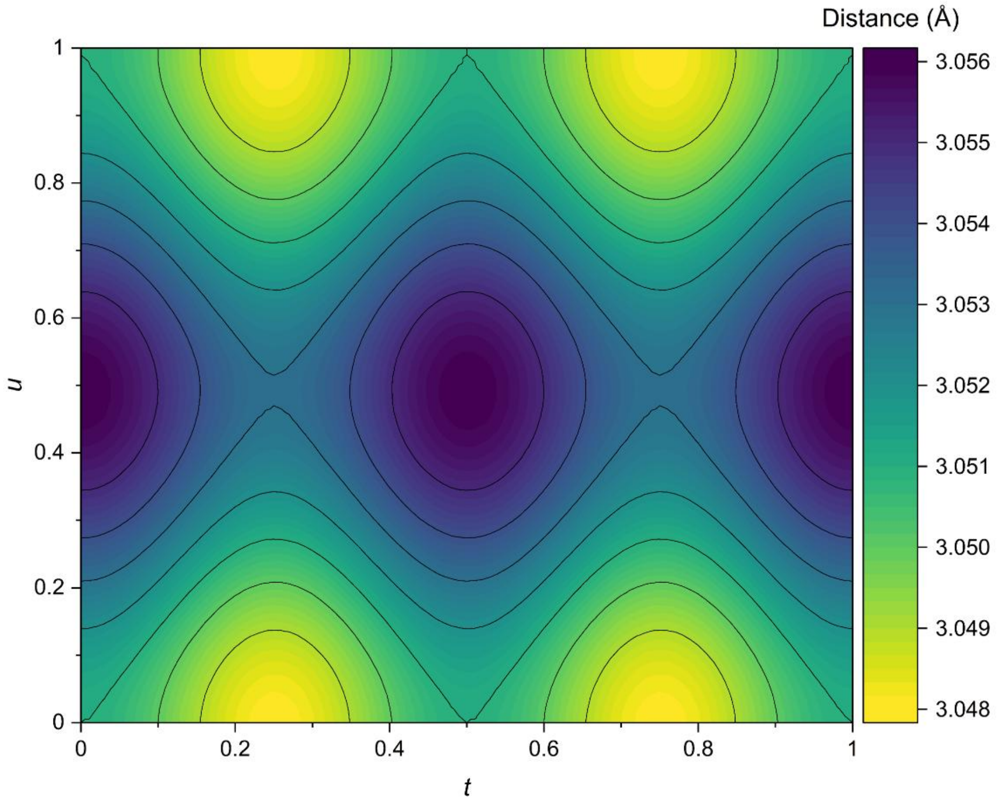

Figure S12. Variation of the Sr-S bond distances against *t* and *u*. Purple regions indicate long bonds and yellow regions indicate short bonds between Sr(1) and S(1). The minimum distance is 3.048 Å and the maximum is 3.055 Å.

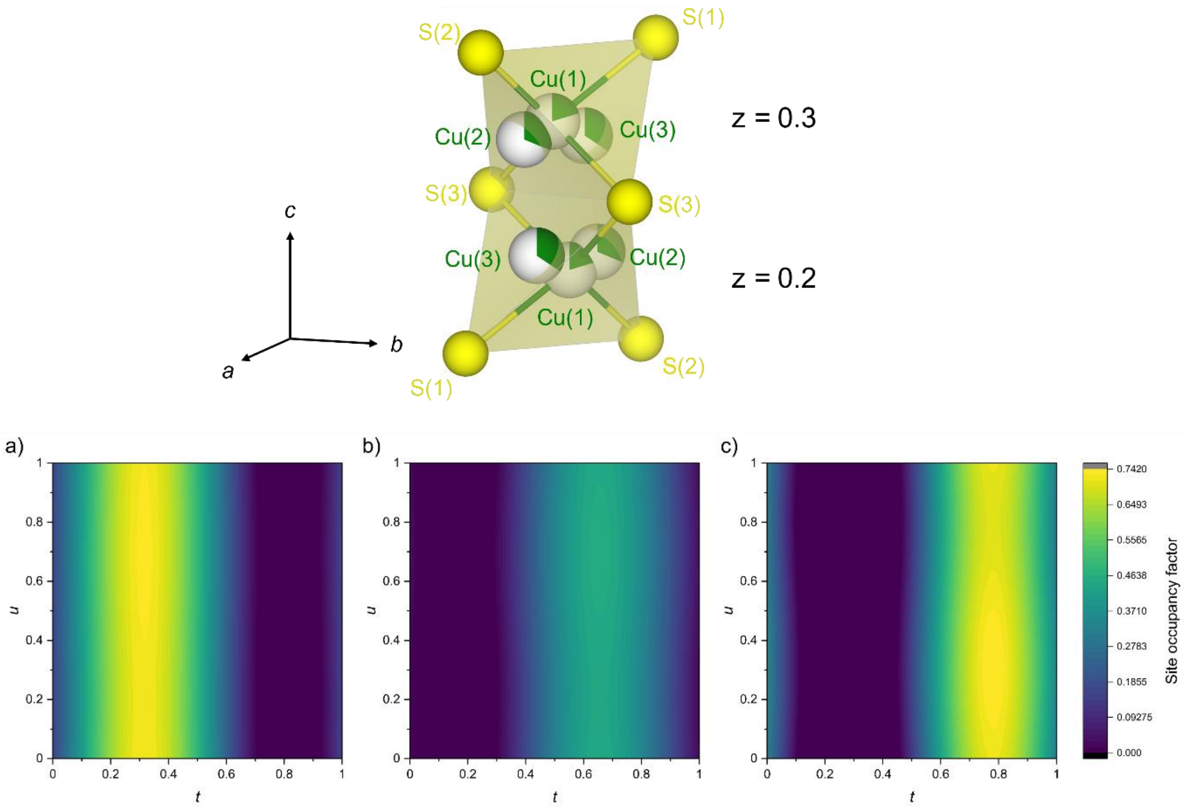

Figure S13. *t*-*u*-plots of the site occupancy factors of (a) Cu(2), (b) Cu(1), and (c) Cu(3) within the copper sulfide layer centred at *z* = 0.25 shown in Figure 10 in the main text. The top figure shows a CuS_4 tetrahedron with atomic labels indicated These *t*-*u* sections represent the variation of the copper site occupancy as a function of *t* and *u.* The pronounced variation with *t* is consistent with modulation of the occupancy of the Cu sites along the *a* direction. Conversely, the minor variations with *u* indicate minimal modulation of the occupancies along the *b* direction The Cu atoms within the copper sulfide layer centred at *z* = 0.75, which are related by the 4_2 screw axis, show the opposite behaviour, i.e. they exhibit strong modulation of Cu occupancy along *b* and little modulation along *a*.

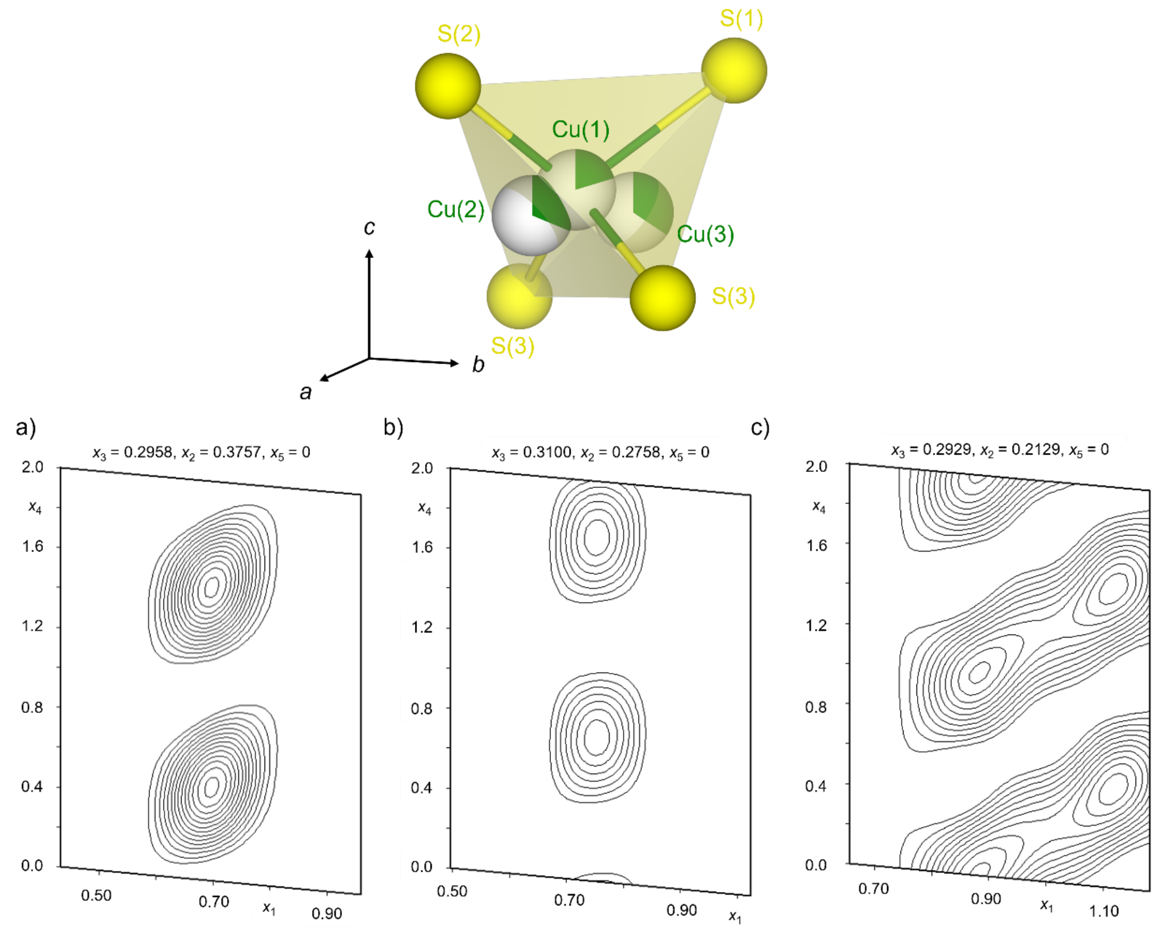

Figure S14. x_1-x_4 de Wolff section showing the observed electron density in superspace centred on the (a) Cu(2), (b) Cu(1) and (c) Cu(3) sites as function of x_4. There is significant modulation of the electron density as a function of x_4 which is determined by the $\mathbf{q_1}$ = 0.2582(5)**a*** vector. High electron density is observed in the Cu(2) map for values of x_4 at which the electron density of the Cu(3) is minimised, supporting the alternation of the occupied triangular face within the CuS_4 tetrahedra.

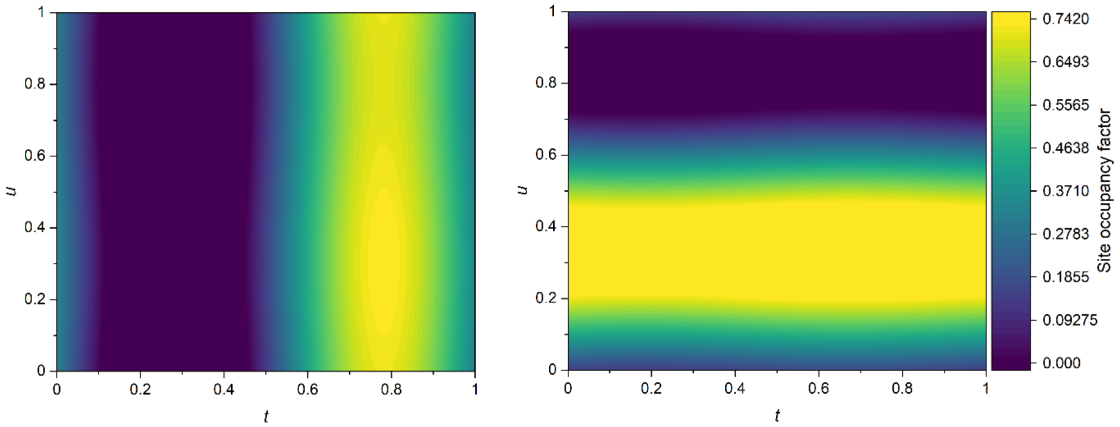

Figure S15. *t*-*u* plots of the site occupancy factor of (left) Cu(2) (0.301, 0.630, 0.2967) and (right) the equivalent atom generated by the 4_2 screw axis Cu(2)#s4t0,1,0 (0.130, 0.199, 0.7967). The Cu(2) atom within the copper sulfide layer centred at *z* = 0.25 shown in Figure 9 in the main text shows significant variation with *t* but not minor changes with *u*. The Cu(2) atom generated by the 4_2 screw axis which lies in the copper sulfide layer centred at *z* = 0.75, shows the opposite behaviour, i.e., significant variation with *u* and minor variations with *t*.

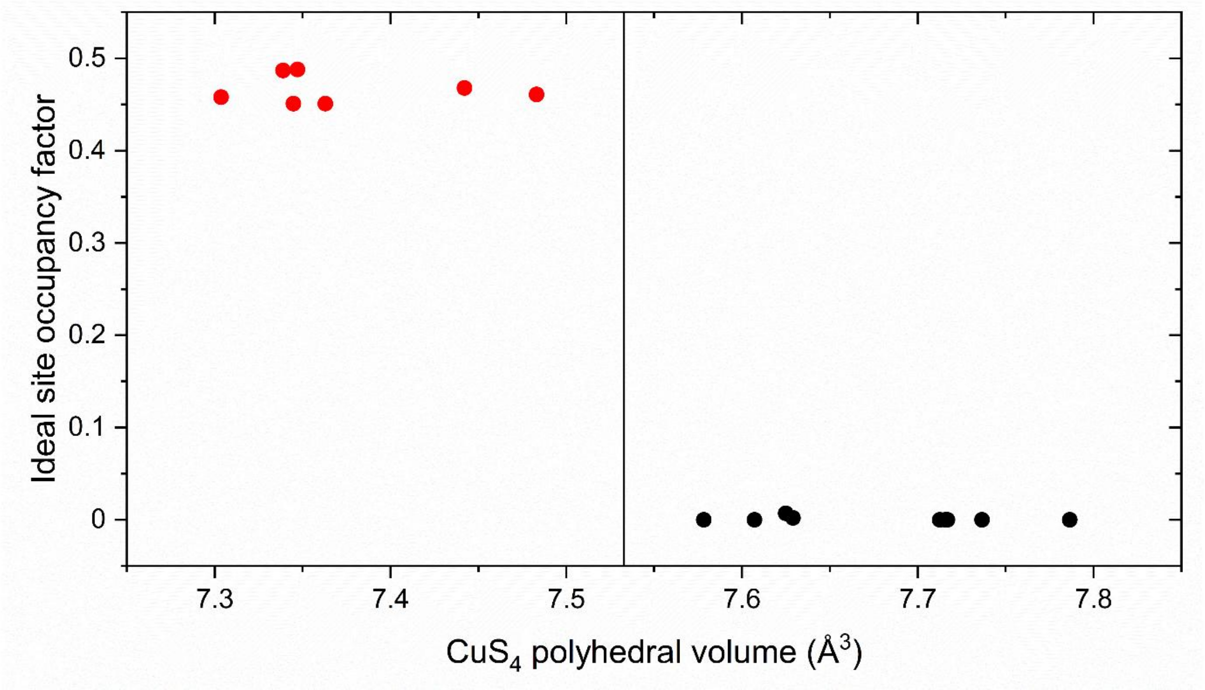

Figure S16. Plot of the site occupancy factor of Cu(1) against the volume of the CuS_4 tetrahedra. Error bars are within the plotted points.

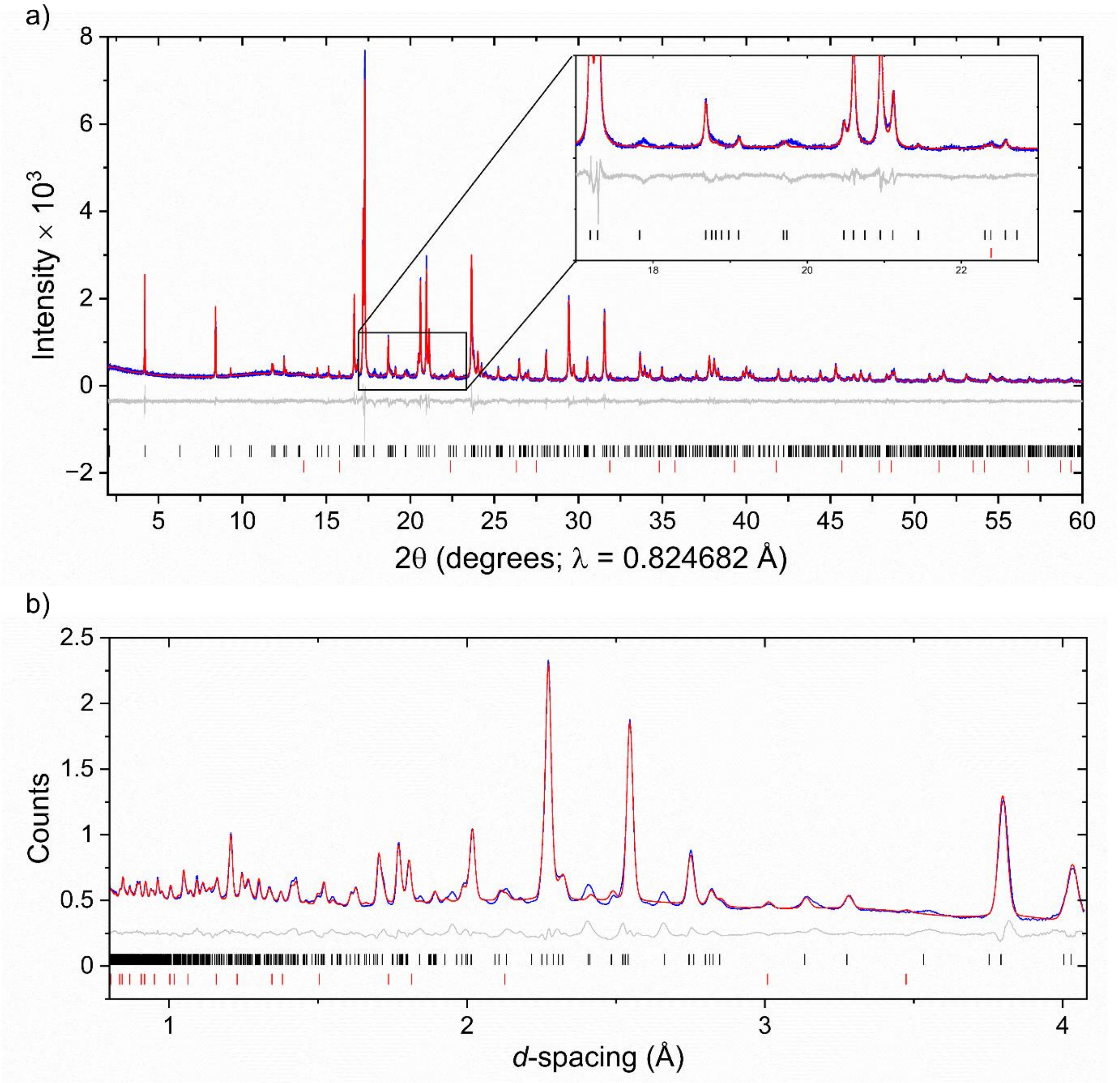

Figure S17. Rietveld refinement of $Sr_2MnO_2Cu_{3.5}S_3$ (sample 1) against (a) synchrotron I11 XRPD data (λ = 0.824682 Å) using the MAC detector and (b) NPD data collected on bank 4 (63.62°) of the GEM instrument at 100 K. The inset shows the region of greatest satellite intensity which are unaccounted for using the average CO model in space group $P4_2/nmc$. Black tick marks indicate the reflections of $Sr_2MnO_2Cu_{3.5}S_3$ (99%) and red tick marks indicate SrS reflections (1%). The corresponding fits using the incommensurately modulated model are shown in the main text (Figure 11).

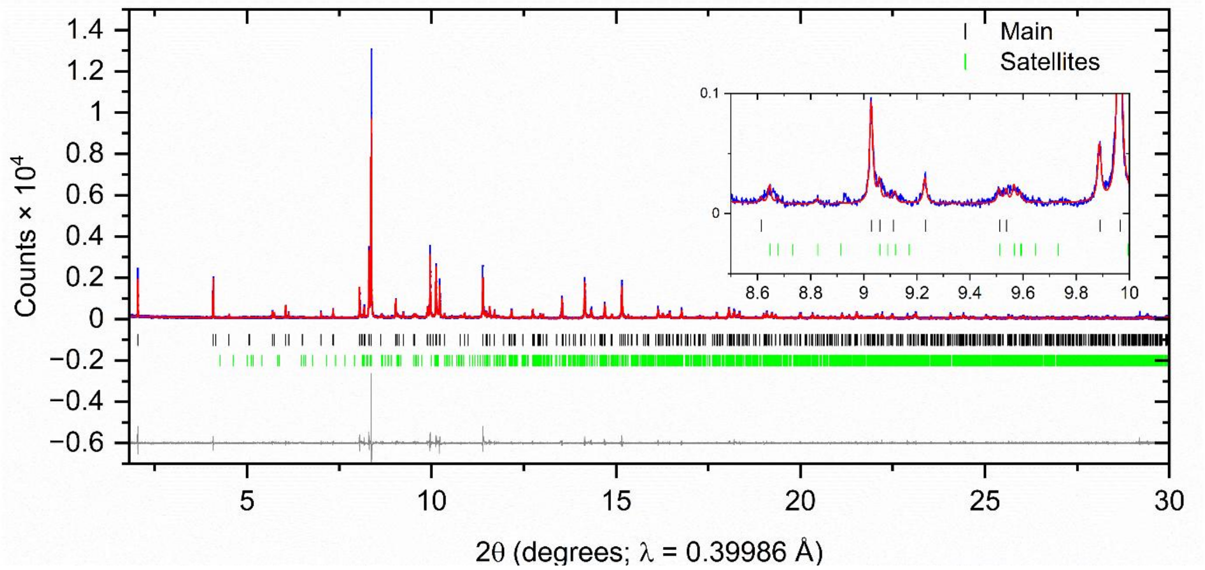

Figure S18. Rietveld refinement of $Sr_2MnO_2Cu_{3.5}S_3$ (sample 3) against synchrotron XRPD data (ID22) collected at 10 K using the superspace model derived from SCXRD analysis. The inset shows the region of greatest satellite intensity. The result is similar to that for sample 1.

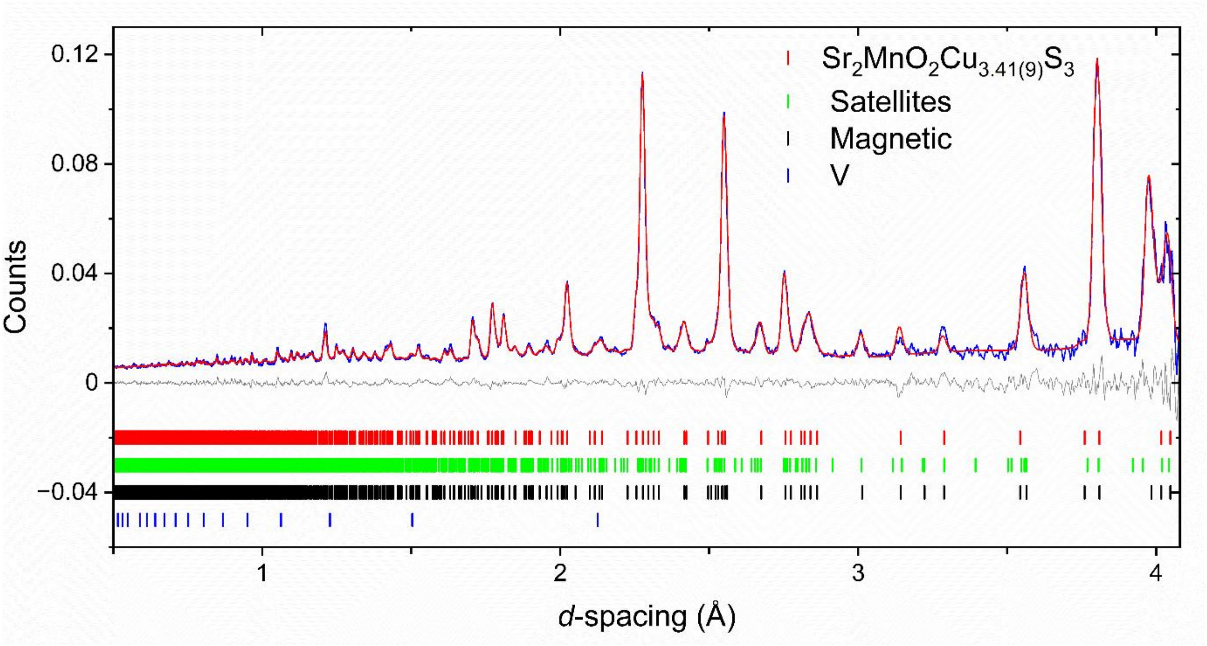

Figure S19a. Rietveld refinement of the crystal and magnetic structures of $Sr_2MnO_2Cu_{3.5}S_3$ against NPD data collected on bank 4 (63.62°) of the GEM instrument at 5 K and 0 T. R_{wp} = 7.255%

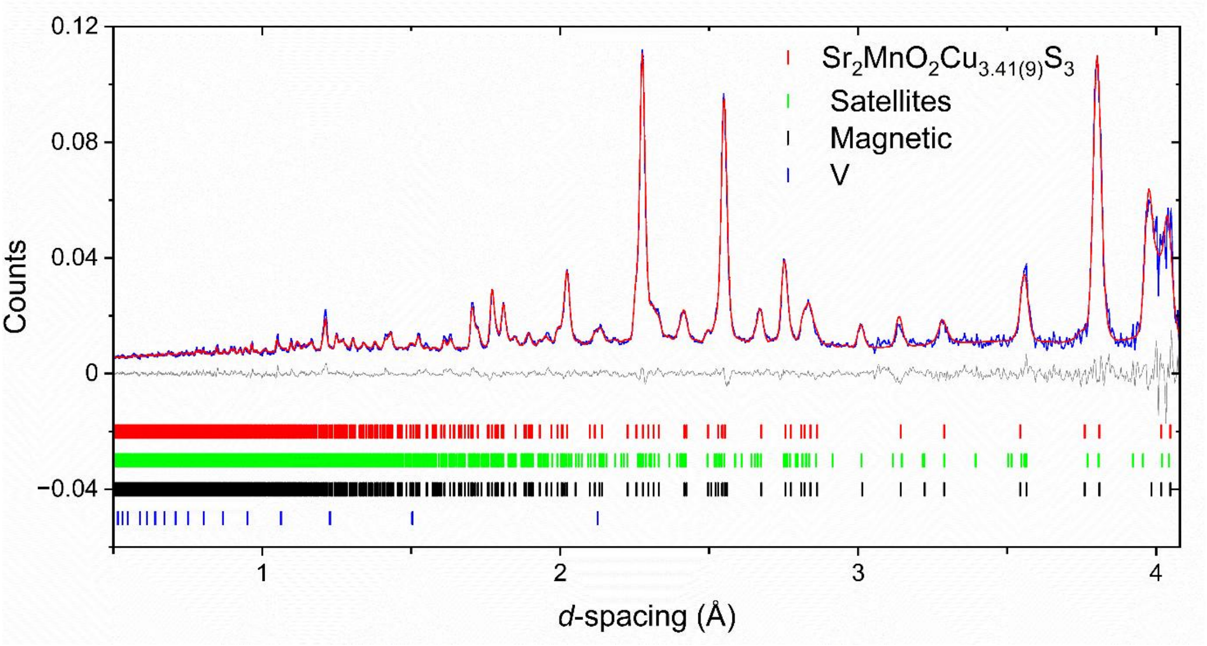

Figure S19b. Rietveld refinement of the crystal and magnetic structures of $Sr_2MnO_2Cu_{3.5}S_3$ against NPD data collected on bank 4 (63.62°) of the GEMinstrument at 5 K and 0.5 T. R_{wp} = 7.029%

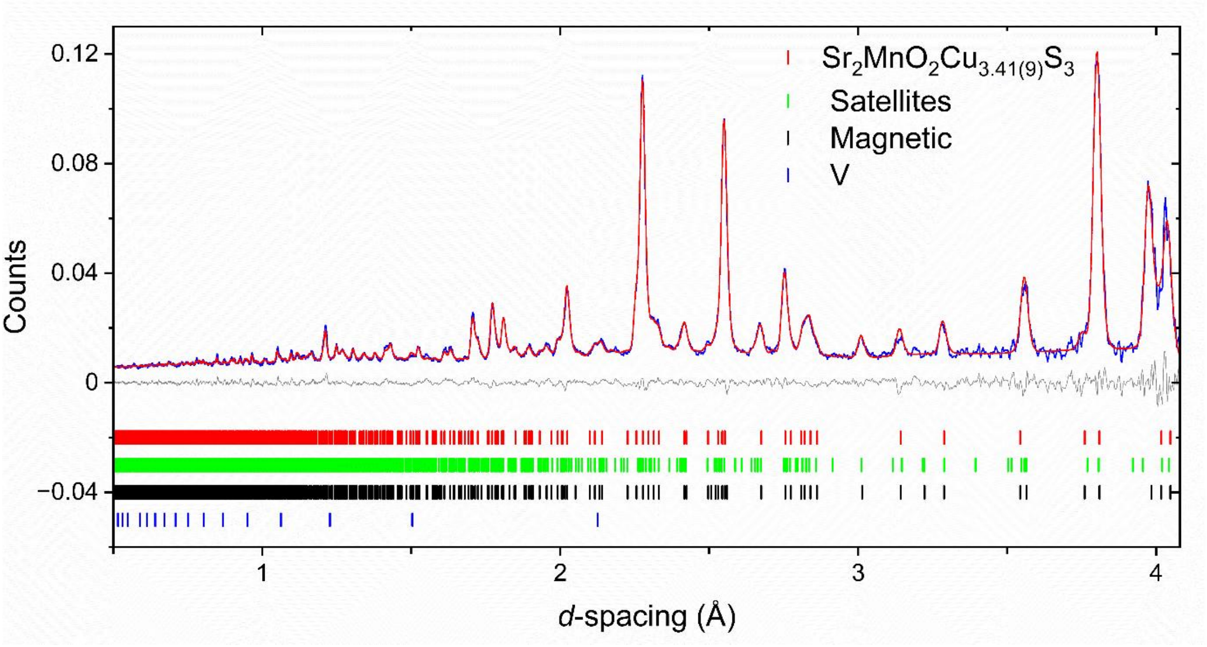

Figure S19c. Rietveld refinement of the crystal and magnetic structures of $Sr_2MnO_2Cu_{3.5}S_3$ against NPD data collected on bank 4 (63.62°) of the GEM instrument at 5 K and 1.1 T. R_{wp} = 6.231%

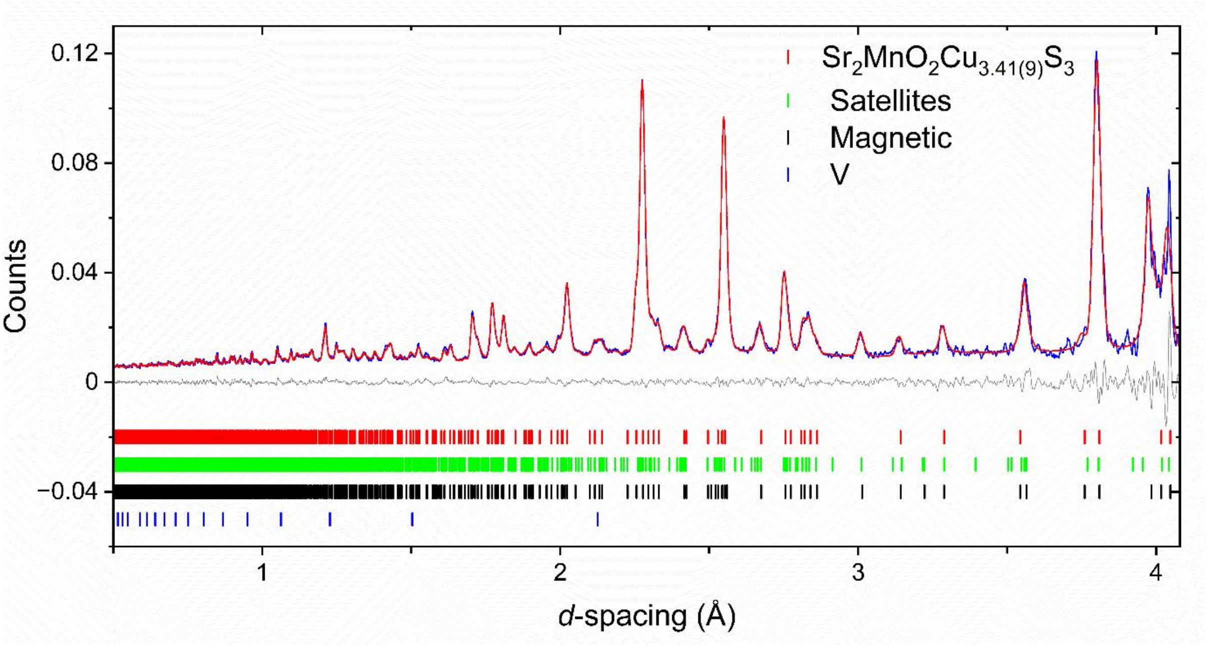

Figure S19d. Rietveld refinement of the crystal and magnetic structures of $Sr_2MnO_2Cu_{3.5}S_3$ against NPD data collected on bank 4 (63.62°) of the GEM instrument at 5 K and 1.4 T. R_{wp} = 7.752%

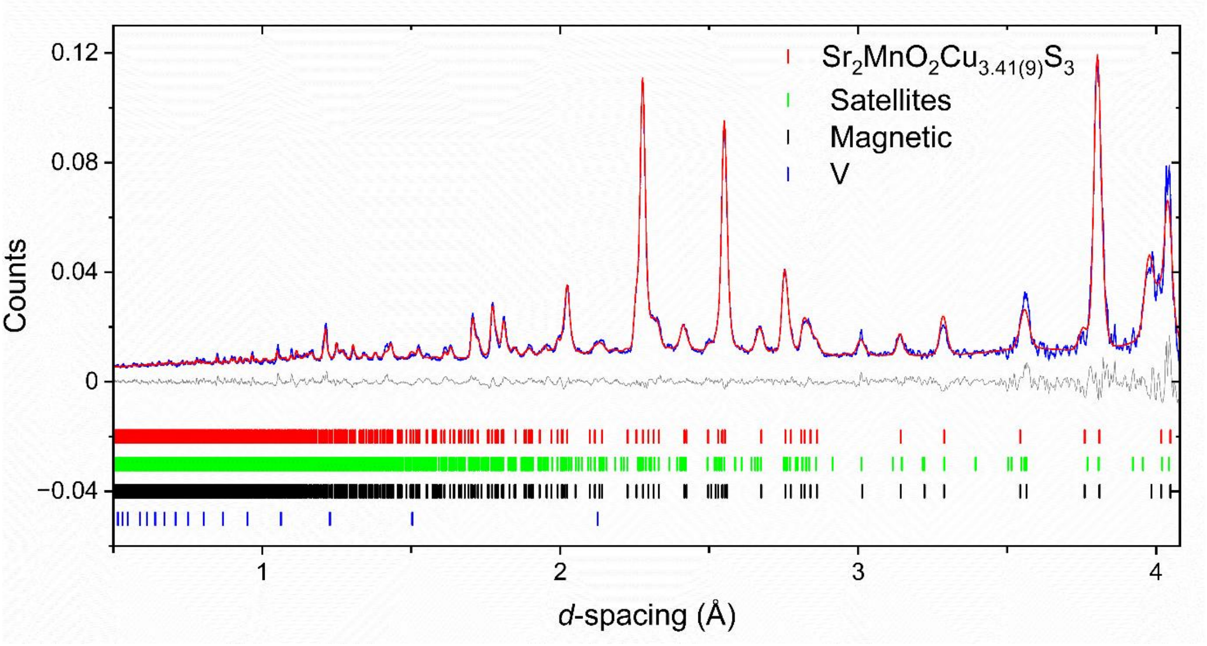

Figure S19e. Rietveld refinement of the crystal and magnetic structures of $Sr_2MnO_2Cu_{3.5}S_3$ against NPD data collected on bank 4 (63.62°) of the GEM instrument at 5 K and 1.5 T. R_{wp} = 7.682%

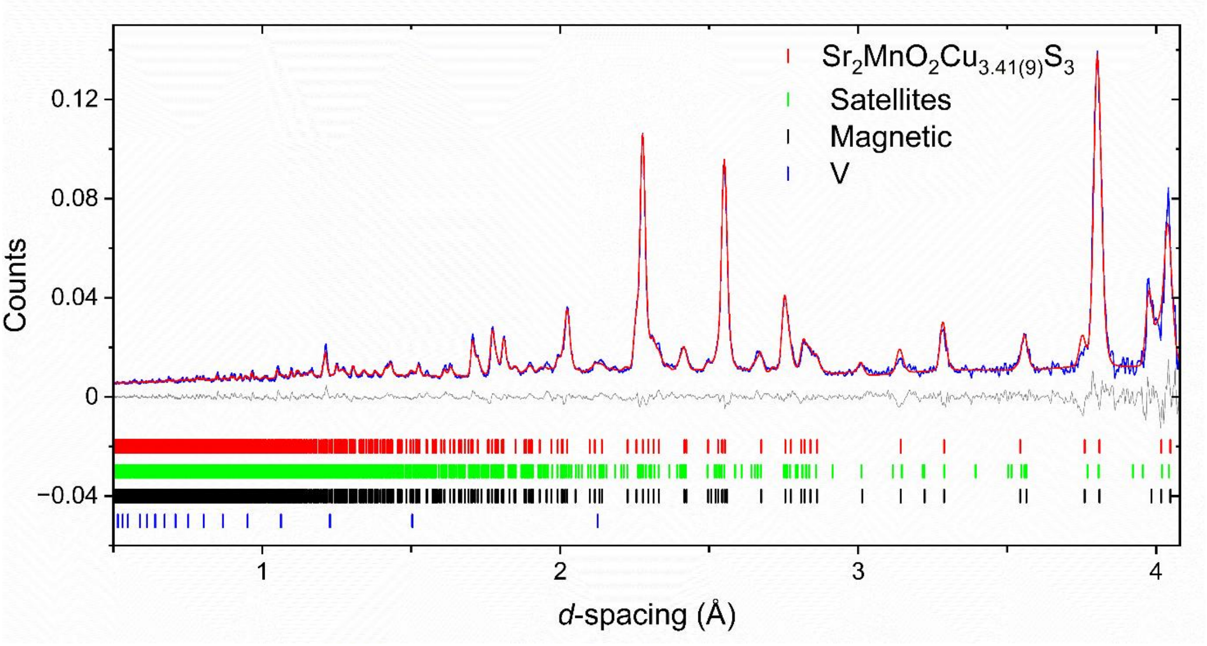

Figure S19f. Rietveld refinement of the crystal and magnetic structures of $Sr_2MnO_2Cu_{3.5}S_3$ against NPD data collected on bank 4 (63.62°) of the GEM instrument at 5 K and 2 T. R_{wp} = 7.819%

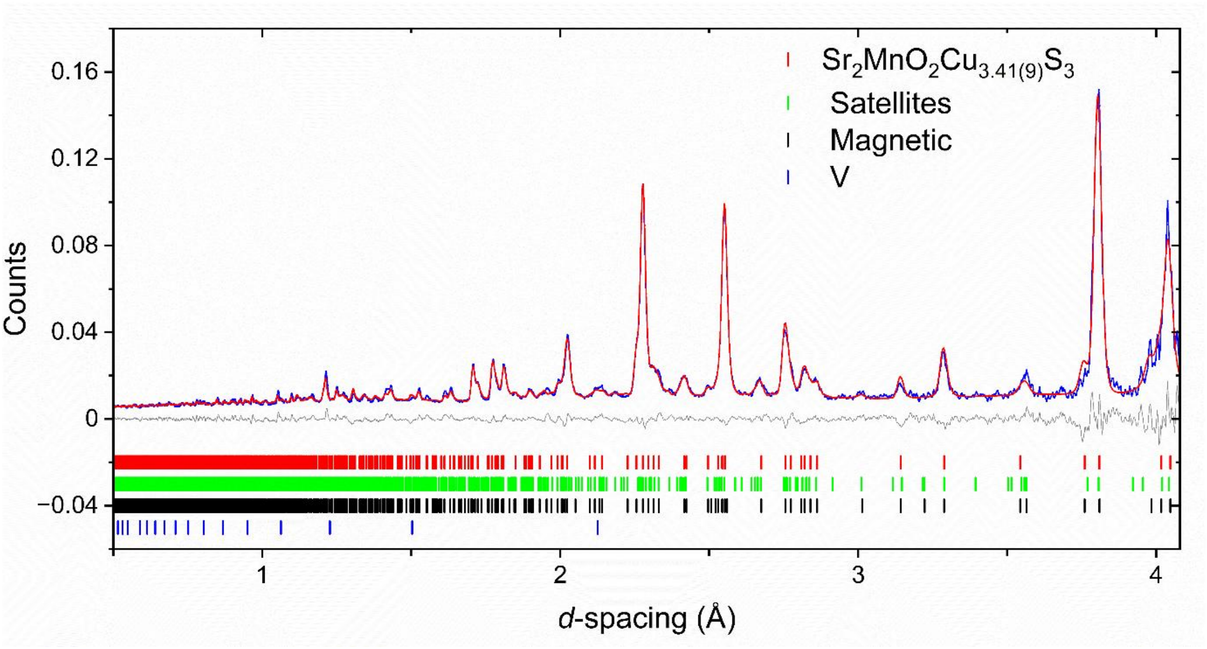

Figure S19g. Rietveld refinement of the crystal and magnetic structures of $Sr_2MnO_2Cu_{3.5}S_3$ against NPD data collected on bank 4 (63.62°) of the GEM instrument at 5 K and 2.5 T. R_{wp} = 8.510%

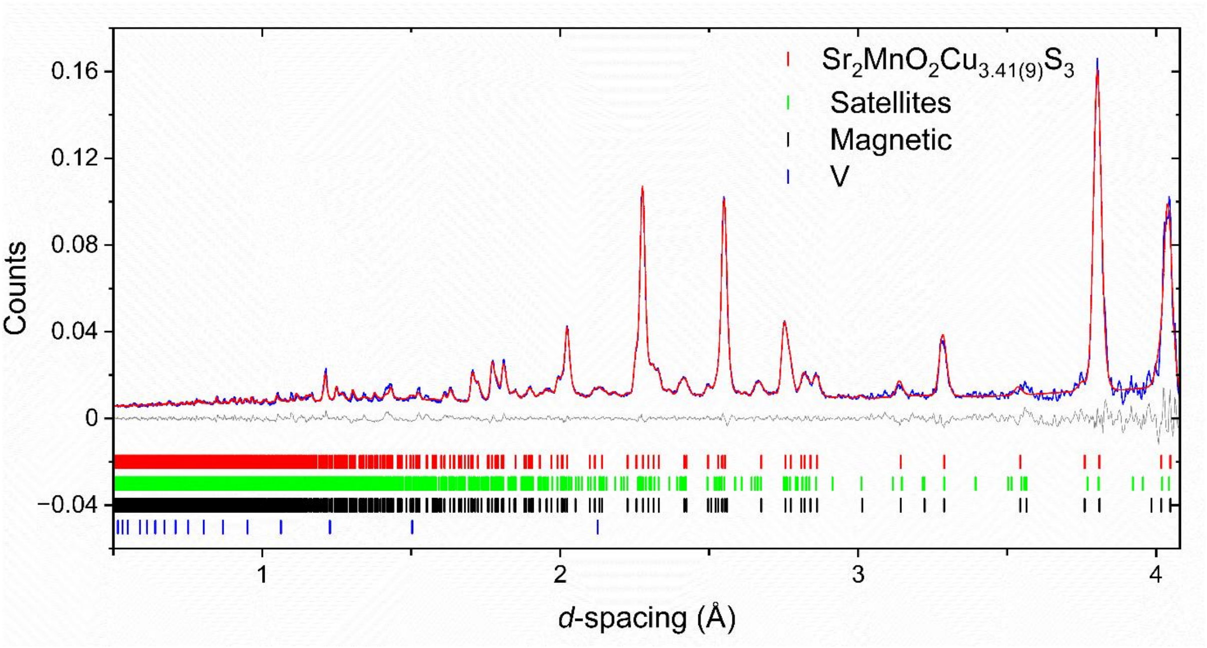

Figure S19h. Rietveld refinement of the crystal and magnetic structures of $Sr_2MnO_2Cu_{3.5}S_3$ against NPD data collected on bank 4 (63.62°) of the GEM instrument at 5 K and 4 T. R_{wp} = 6.710%

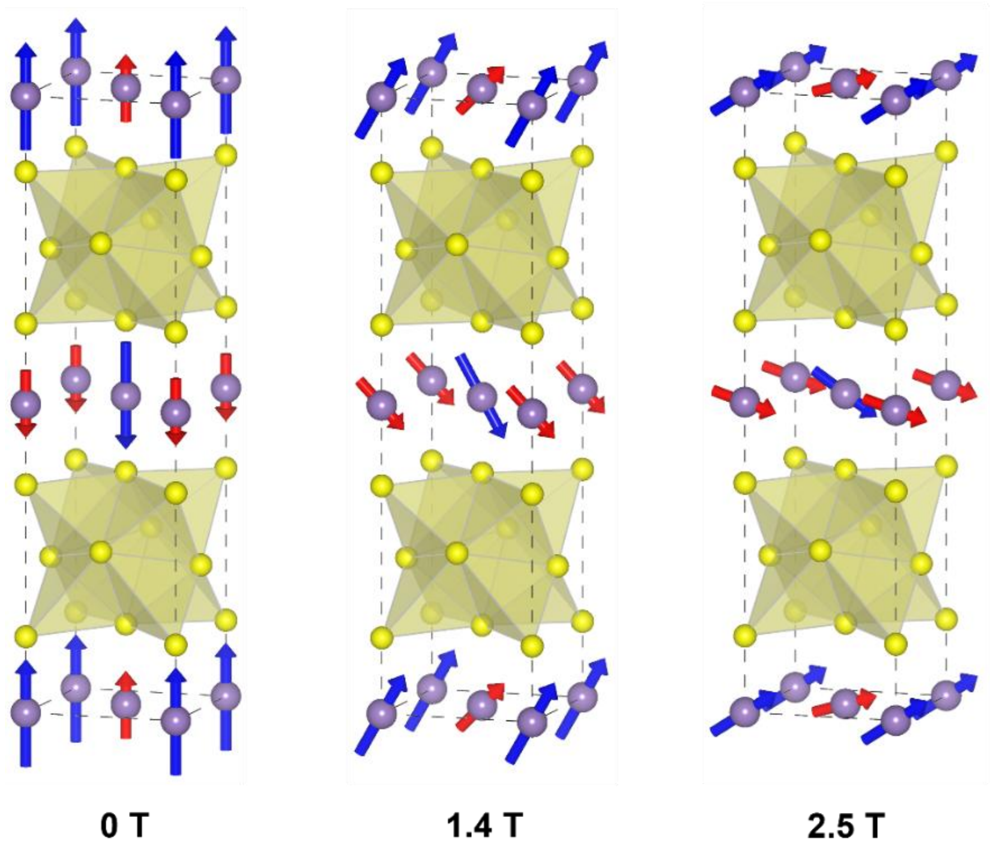

Figure S20. Magnetic unit cell of $Sr_2MnO_2Cu_{3.5}S_3$ over the magnetic field range 0-2.5 T showing the magnitude and direction of magnetic moments for the two manganese sublattices. The external field induces a ferromagnetic component of the magnetism that eventually replaces the antiferromagnetic component entirely by 5 T as indicated by Figure 21 in the main text. Mn (lilac) and S (yellow) atoms are shown for clarity.

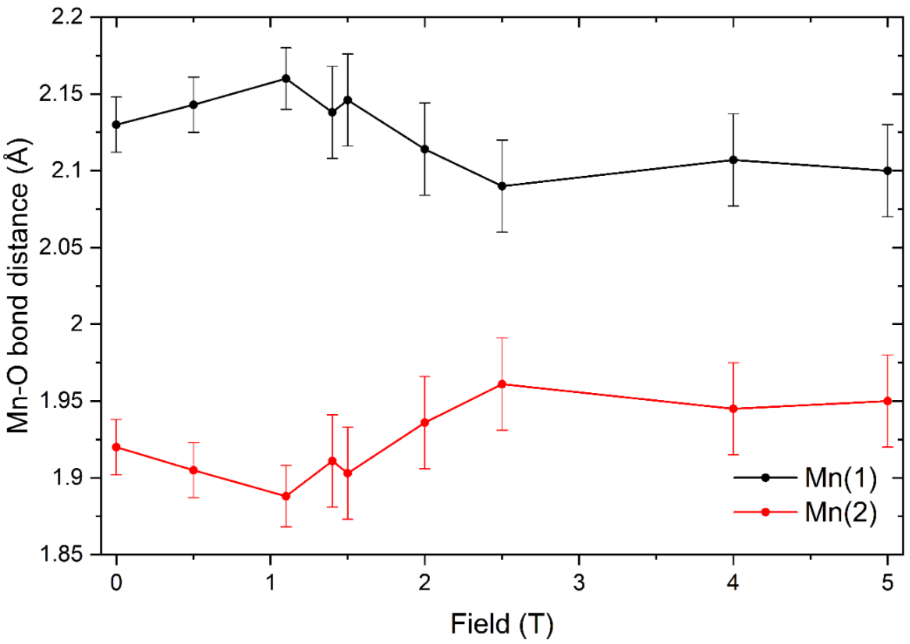

Figure S21. Field-dependent variation of Mn-O bond distances in $Sr_2MnO_2Cu_{3.5}S_3$ from 0 to 5 T as determined by Rietveld analysis of NPD patterns collected on the GEM instrument

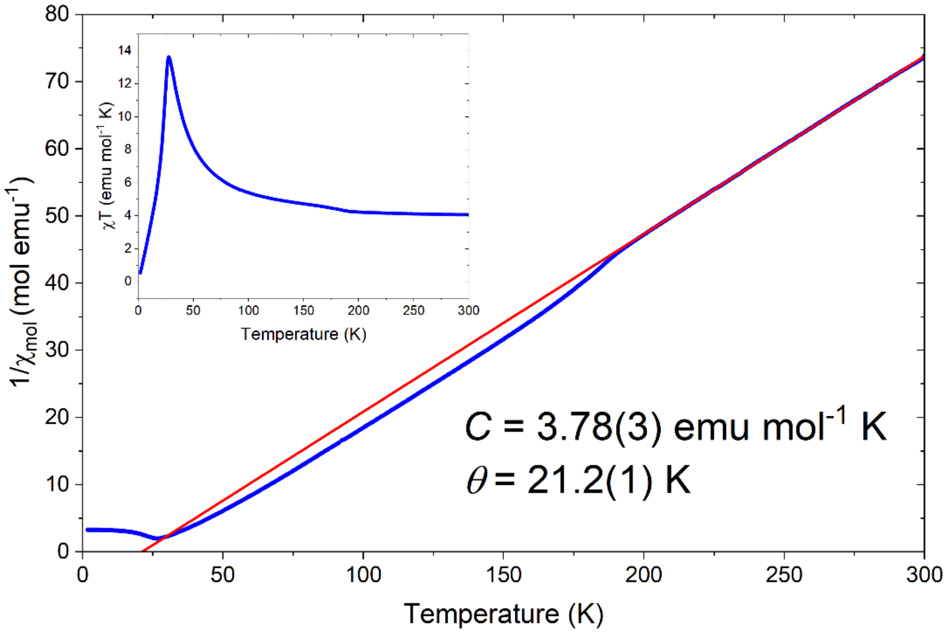

Figure S22. Plot of the inverse magnetic susceptibility against temperature. The red line is an extrapolation of the Curie-Weiss fitting to the linear region between 200 - 300 K which produces the parameters given in the text. The fit was calculated using the Curie (χ_{mol} = C/T-θ) where C is the Curie constant and θ is the Weiss constant. The inset shows a plot of $\chi_{mol}T$ against temperature.

Supplementary Tables

Table S2. Selected bond lengths for $Sr_2MnO_2Cu_{3.5}S_3$ at 293 K (GEM and ID22 data)

Atoms	Distance (Å)	Atoms	Angle (°)
Mn(1)-O(1) [4]	2.008170(1)	S(1)-Cu(1)-S(1)	107.62(8)
Mn(1)-S(1) [2]	2.8769(6)	S(2)-Cu(1)-S(2)	111.89(8)
		S(1)-Cu(1)-S(2)	109.309(6)
Cu(1)-S(1) [2]	2.4883(11)		
Cu(1)-S(2) [2]	2.4239(10)	S(1)-Cu(2)-S(2)	121.56(5)
Cu(2)-S(1) [1]	2.229(2)		
Cu(2)-S(2) [2]	2.3618(11)		
S(1)-S(2)	4.0069(5)		

Table S3. Single crystal refinement details of the average structure at 100 K in space group $P4_2/nmc$

**Crystal data**	
Chemical formula	$Sr_2MnO_2Cu_{3.431}S_3$
M_r	576.4
Crystal system, space group	Tetragonal, $P4_2/nmc$
Temperature (K)	100
a, c (Å)	5.69216 (15), 22.4854 (6)
V (Å^3)	728.54 (3)
Z	4
Radiation type	Mo $K\alpha$
μ (mm^{-1})	26.82
Crystal size (mm)	0.17 × 0.16 × 0.03
Data collection	
Diffractometer	Oxford Diffraction CCD
Absorption correction	Multi-scan
No. of measured, independent and observed [$I > 3\sigma(I)$] reflections	22996, 514, 414
R_{int}	0.119
$(\sin \theta/\lambda)_{max}$ (Å^{-1})	0.650
**Refinement**	
$R[F^2 > 2\sigma(F^2)]$, $wR(F^2)$, S	0.043, 0.129, 2.78
No. of reflections	514
No. of parameters	60
$\Delta\rho_{max}$, $\Delta\rho_{min}$ (e Å^{-3})	1.13, −0.88

Table S4. Single crystal refinement details of the average structure at 100 K in space group *Pmmn*

**Crystal data**	
Chemical formula	$Sr_2MnO_2Cu_{3.431}S_3$
M_r	576.4
Crystal system, space group	Orthorhombic, *Pmmn*
Temperature (K)	100
a, *c* (Å)	5.69216 (15), 22.4854 (6)
V (Å^3)	728.54 (3)
Z	4
Radiation type	Mo *K*α
μ (mm^{-1})	26.82
Crystal size (mm)	0.17 × 0.16 × 0.03
Data collection	
Diffractometer	Oxford Diffraction CCD
Absorption correction	Multi-scan
No. of measured, independent and observed [$I > 3\sigma(I)$] reflections	24740, 1025, 731
R_{int}	0.118
$(\sin \theta/\lambda)_{max}$ (Å^{-1})	0.650
**Refinement**	
$R[F^2 > 2\sigma(F^2)]$, $wR(F^2)$, S	0.046, 0.137, 2.23
No. of reflections	1025
No. of parameters	112
$\Delta\rho_{max}$, $\Delta\rho_{min}$ (e Å^{-3})	3.02, -1.71

Table S5. Bond valence sums of $Sr_2MnO_2Cu_{3.5}S_3$ (sample 2) derived from structural refinements against NPD data collected on the GEM instrument. The values are calculated using the formula $v_i = \Sigma e^{-\left(\frac{R_0-R_i}{B}\right)}$, where R_0 is the literature reported bond length based on several compounds, R_i is the observed bond length, and *B* is a constant[10]. In these calculations we use *B* = 0.417 for bonds to O atoms and 0.37 for bonds to S atoms.

Temperature (K)	Mn(1)	Mn(2)
220	2.499	
190	2.395	2.762
180	2.166	2.981
160	2.153	3.044
100	2.114	3.074
50	2.096	3.089
30	2.116	3.071

Table S6. Modulation parameters of the atomic positions in $Sr_2MnO_2Cu_{3.5}S_3$

Displacive modulations were introduced to describe the displacement of all atoms according to the formula:

$$u_i = A_{i1}\sin(2\pi\bar{x}_4) + A_{i2}\sin(2\pi\bar{x}_5) + B_{i1}\cos(2\pi\bar{x}_4) + B_{i2}\cos(2\pi\bar{x}_5)$$

where *i* represents the *x*, *y*, *z* dimensions of real space and A_i, B_i, C_i, and D_i are the amplitudes of the Fourier terms

Atom	Harmonic	x	y	z
Sr(1)				
	A_{i1}	0	0.0019(1)	0
	A_{i2}	0.0031(1)	0	0
	B_{i1}	0	0	0
	B_{i2}	0	0	0
Sr(2)				
	A_{i1}	0	-0.0022(1)	0
	A_{i2}	0.0099(1)	0	0
	B_{i1}	0	0	0
	B_{i2}	0	0	0
Mn(1)				
	A_{i1}	0	-0.0011(2)	0
	A_{i2}	0.0011(2)	0	0
	B_{i1}	0	0	0
	B_{i2}	0	0	0
Mn(2)				
	A_{i1}	0	-0.0050(2)	0
	A_{i2}	0.0050(2)	0	0
	B_{i1}	0	0	0
	B_{i2}	0	0	0
S(1)				
	A_{i1}	0	-0.0211(7)	0
	A_{i2}	0.0027(3)	0	0
	B_{i1}	0	0	0
	B_{i2}	0	0	0
S(2)				
	A_{i1}	0	0.0046(3)	0
	A_{i2}	0.0024(3)	0	0
	B_{i1}	0	0	0
	B_{i2}	0	0	0
S(3)				
	A_{i1}	0	0.0161(3)	0
	A_{i2}	0.0018(3)	0	0
	B_{i1}	0	0	0
	B_{i2}	0	0	0
O(1)				
	A_{i1}	-0.0003(4)	-0.0038(3)	0.0001(1)

	A_{i2}	0.0038(3)	0.0003(4)	0.0001(1)
	B_{i1}	-0.0002(4)	0.0017(4)	0.0001(1)
	B_{i2}	0.0017(4)	-0.0002(4)	0.0001(1)
Cu(1)				
	A_{i1}	-0.025(1)	0.009(2)	-0.0028(4)
	A_{i2}	0.002(2)	0.002(2)	-0.0003(5)
	B_{i1}	-0.005(3)	0.006(3)	-0.0017(7)
	B_{i2}	0.000	0.000	0.0001(5)
Cu(2)				
	A_{i1}	0.0426(6)	0.0137(4)	0.0027(1)
	A_{i2}	0.0023(8)	-0.0013(4)	-0.00011(9)
	B_{i1}	-0.0629(13)	-0.0119(8)	-0.0003(2)
	B_{i2}	0.0026(7)	0.0011(4)	0.00016(9)
Cu(3)				
	A_{i1}	0.002(3)	-0.030(3)	0.0006(6)
	A_{i2}	0.002(2)	0.001(5)	-0.000
	B_{i1}	0.0034(9)	-0.011(1)	0.0002(2)
	B_{i2}	0.000(3)	-0.000(5)	0.0001(5)

Table S7. Modulation parameters of the anisotropic displacement parameters in $Sr_2MnO_2Cu_{3.5}S_3$

Atom	A_{U11}	A_{U22}	A_{U33}	A_{U12}	A_{U13}	A_{U23}
Sr(1)	0	0	0	0	0	-0.0002(3)
Sr(2)	0	0	0	0	0	-0.0001(3)
Mn(1)	0	0	0	0	0	0
Mn(2)	0	0	0	0	0	0
S(1)	0	0	0	0	0	0.0033(7)
S(2)	0	0	0	0	0	0.0012(7)
S(3)	0	0	0	0	0	-0.0016(7)
O(1)	0	0	0	0	0	0
Cu(1)	0.001(3)	-0.000(3)	0.000(2)	-0.001(3)	0.000(2)	-0.001(2)
Cu(2)	-0.000(2)	0.002(3)	-0.000(1)	-0.000(2)	0.0009(8)	0.002(2)
Cu(3)	-0.002(5)	-0.001(3)	-0.001(2)	-0.002(3)	-0.001(3)	-0.001(2)
Atom	B_{U11}	B_{U22}	B_{U33}	B_{U12}	B_{U13}	B_{U23}
Sr(1)	0	0	0	0.0000(3)	0	0
Sr(2)	0	0	0	-0.0001(3)	0	0
Mn(1)	0	0	0	0	0	0
Mn(2)	0	0	0	0	0	0
S(1)	0	0	0	0.0027(8)	0	0
S(2)	0	0	0	-0.0009(7)	0	0
S(3)	0	0	0	0.0044(8)	0	0
O(1)	0	0	0	0	0	0
Cu(1)	-0.002(3)	0.000(3)	-0.000(2)	0.000(3)	-0.000(2)	0.000(2)
Cu(2)	-0.000(1)	-0.011(3)	0.000(1)	-0.003(2)	0.0002(9)	0.000(2)
Cu(3)	-0.002(5)	-0.000(3)	-0.000(2)	0.000(3)	0.002(3)	0.000(2)

Table S8. Modulation parameters of the site occupancy factors for the Cu atoms in $Sr_2MnO_2Cu_{3.5}S_3$

Occupancy modulations were introduced for all Cu atoms

$$p_i = p_0 + A_{i1}\sin(2\pi\bar{x}_4) + A_{i2}\sin(2\pi\bar{x}_4) + A_{i3}\sin(2\pi\bar{x}_5) + A_{i4}\sin(2\pi\bar{x}_5) + B_{i1}\cos(2\pi\bar{x}_4) + B_{i2}\cos(2\pi\bar{x}_4) + B_{i3}\cos(2\pi\bar{x}_5) + B_{i4}\cos(2\pi\bar{x}_5)$$

where p_0 is the average occupancy, *i* represents the *x*, *y*, *z* dimensions of real space and A_i, B_i, C_i… are the amplitudes of the Fourier terms.

Atom	Avg.	A_{i1}	A_{i2}	A_{i3}	A_{i4}	B_{i1}	B_{i2}	B_{i3}	B_{i4}
Cu(1)	0.204(19)	-0.26(1)	0.01(3)	0(1)	0(1)	-0.044(7)	0.00(3)	0.013(7)	0.01(3)
Cu(2)	0.311(19)	0.12(1)	-0.01(2)	0(1)	0(1)	-0.477(7)	0.02(3)	0.041(7)	0.04(3)
Cu(3)	0.343(5)	0.134(2)	0.005(6)	0(1)	0(1)	0.383(5)	0.000(6)	0.010(5)	0.010(6)

References

(1) Adamson, P.; Hadermann, J.; Smura, C. F.; Rutt, O. J.; Hyett, G.; Free, D. G.; Clarke, S. J. Competing Magnetic Structures and the Evolution of Copper Ion/Vacancy Ordering with Composition in the Manganite Oxide Chalcogenides $Sr_2MnO_2Cu_{1.5}(S_{1-x}Se_x)_2$. *Chemistry of Materials* **2012**, *24* (14), 2802–2816. https://doi.org/10.1021/cm301486v.

(2) Smura, C. F.; Parker, D. R.; Zbiri, M.; Johnson, M. R.; Gál, Z. A.; Clarke, S. J. High-Spin Cobalt(II) Ions in Square Planar Coordination: Structures and Magnetism of the Oxysulfides $Sr_2CoO_2Cu_2S_2$ and $Ba_2CoO_2Cu_2S_2$ and Their Solid Solution. *J Am Chem Soc* **2011**, *133* (8), 2691–2705. https://doi.org/10.1021/ja109553u.

(3) Smyth, R. D.; Blandy, J. N.; Yu, Z.; Liu, S.; Topping, C. V.; Cassidy, S. J.; Smura, C. F.; Woodruff, D. N.; Manuel, P.; Bull, C. L.; Funnell, N. P.; Ridley, C. J.; McGrady, J. E.; Clarke, S. J. High-versus Low-Spin Ni^{2+} in Elongated Octahedral Environments: $Sr_2NiO_2Cu_2Se_2$, $Sr_2NiO_2Cu_2S_2$, and $Sr_2NiO_2Cu_2(Se_{1-x}S_x)_2$. *Chemistry of Materials* **2022**, *34* (21), 9503–9516. https://doi.org/10.1021/acs.chemmater.2c02002.

(4) Blandy, J. N.; Liu, S.; Smura, C. F.; Cassidy, S. J.; Woodruff, D. N.; McGrady, J. E.; Clarke, S. J. Synthesis, Structure, and Properties of the Layered Oxide Chalcogenides $Sr_2CuO_2Cu_2S_2$ and $Sr_2CuO_2Cu_2Se_2$. *Inorg Chem* **2018**, *57* (24), 15379–15388. https://doi.org/10.1021/acs.inorgchem.8b02698.

(5) Zhu, W. J.; Hor, P. H. Unusual Layered Transition-Metal Oxysulfides: $Sr_2Cu_2MO_2S_2$ (M=Mn, Zn). *J Solid State Chem* **1997**, *130* (2), 319–321. https://doi.org/10.1006/jssc.1997.7299.

(6) Dhesi, S. S.; Mirone, A.; De Nadaï, C.; Ohresser, P.; Bencok, P.; Brookes, N. B.; Reutler, P.; Revcolevschi, A.; Tagliaferri, A.; Toulemonde, O.; van der Laan, G. Unraveling Orbital Ordering in $La_{0.5}Sr_{1.5}MnO_4$. *Phys Rev Lett* **2004**, *92* (5), 056403. https://doi.org/10.1103/PhysRevLett.92.056403.

(7) Wilkins, S. B.; Spencer, P. D.; Hatton, P. D.; Collins, S. P.; Roper, M. D.; Prabhakaran, D.; Boothroyd, A. T. Direct Observation of Orbital Ordering in $La_{0.5}Sr_{1.5}MnO_4$ Using Soft X-Ray Diffraction. *Phys Rev Lett* **2003**, *91* (16), 167205. https://doi.org/10.1103/PhysRevLett.91.167205.

(8) Sternlieb, B. J.; Hill, J. P.; Wildgruber, U. C.; Luke, G. M.; Nachumi, B.; Moritomo, Y.; Tokura, Y. Charge and Magnetic Order in $La_{0.5}Sr_{1.5}MnO_4$. *Phys Rev Lett* **1996**, *76* (12), 2169–2172. https://doi.org/10.1103/PhysRevLett.76.2169.

(9) Moritomo, Y.; Nakamura, A.; Mori, S.; Yamamoto, N.; Ohoyama, K.; Ohashi, M. Lattice Effects on the Charge-Ordering Transition $R_{0.5}Sr_{1.5}MnO_4$. *Phys Rev B* **1997**, *56* (23), 14879–14882. https://doi.org/10.1103/PhysRevB.56.14879.

(10) de Wolff, P. M. The Pseudo-Symmetry of Modulated Crystal Structures. *Acta Crystallographica Section A* 1974, *30* (6), 777–785. https://doi.org/10.1107/S0567739474010710.

(11) Brese, N. E.; O'Keeffe, M. Bond-Valence Parameters for Solids. *Acta Crystallogr B* 1991, *47* (2), 192–197. https://doi.org/10.1107/S0108768190011041.
